# Evolutionary pattern of karyotypes and meiosis in pholcid spiders (Araneae: Pholcidae): implications for reconstructing chromosome evolution of araneomorph spiders

**DOI:** 10.1186/s12862-021-01750-8

**Published:** 2021-05-03

**Authors:** Ivalú M. Ávila Herrera, Jiří Král, Markéta Pastuchová, Martin Forman, Jana Musilová, Tereza Kořínková, František Šťáhlavský, Magda Zrzavá, Petr Nguyen, Pavel Just, Charles R. Haddad, Matyáš Hiřman, Martina Koubová, David Sadílek, Bernhard A. Huber

**Affiliations:** 1 Laboratory of Arachnid Cytogenetics, Department of Genetics and Microbiology, Faculty of Science, Charles University, Viničná 5, 128 44 Prague 2, Czech Republic; 2 Research Team of Plant Stress Biology and Biotechnology, Division of Crop Genetics and Breeding, Crop Research Institute, Drnovská 507/73, 161 00 Prague 6, Czech Republic; 3 Prague 1, Czech Republic; 4 Invertebrate Zoology Unit, Department of Zoology, Faculty of Science, Charles University, Viničná 7, 128 44 Prague 2, Czech Republic; 5 Laboratory of Molecular Cytogenetics, Department of Molecular Biology and Genetics, Faculty of Science, University of South Bohemia, Branišovská 31, 370 05 České Budějovice, Czech Republic; 6 Laboratory of Molecular Cytogenetics, Department of Molecular Biology and Genetics, Institute of Entomology, Biology Centre CAS, Branišovská 31, 370 05 České Budějovice, Czech Republic; 7 Research Group of Arachnid Systematics and Ecology, Department of Zoology and Entomology, Faculty of Natural and Agricultural Sciences, University of the Free State, P.O. Box 339, Bloemfontein, 9300 Republic of South Africa; 8 Arachnida Section, Alexander Koenig Zoological Research Museum, Adenauerallee 160, 53113 Bonn, Germany

**Keywords:** Achiasmatic pairing, Diffuse stage, Entelegyne, Haplogyne, Inactivation, rDNA, Rearrangement, Segregation, Y chromosome

## Abstract

**Background:**

Despite progress in genomic analysis of spiders, their chromosome evolution is not satisfactorily understood. Most information on spider chromosomes concerns the most diversified clade, entelegyne araneomorphs. Other clades are far less studied. Our study focused on haplogyne araneomorphs, which are remarkable for their unusual sex chromosome systems and for the co-evolution of sex chromosomes and nucleolus organizer regions (NORs); some haplogynes exhibit holokinetic chromosomes. To trace the karyotype evolution of haplogynes on the family level, we analysed the number and morphology of chromosomes, sex chromosomes, NORs, and meiosis in pholcids, which are among the most diverse haplogyne families. The evolution of spider NORs is largely unknown.

**Results:**

Our study is based on an extensive set of species representing all major pholcid clades. Pholcids exhibit a low 2n and predominance of biarmed chromosomes, which are typical haplogyne features. Sex chromosomes and NOR patterns of pholcids are diversified. We revealed six sex chromosome systems in pholcids (X0, XY, X_1_X_2_0, X_1_X_2_X_3_0, X_1_X_2_Y, and X_1_X_2_X_3_X_4_Y). The number of NOR loci ranges from one to nine. In some clades, NORs are also found on sex chromosomes.

**Conclusions:**

The evolution of cytogenetic characters was largely derived from character mapping on a recently published molecular phylogeny of the family. Based on an extensive set of species and mapping of their characters, numerous conclusions regarding the karyotype evolution of pholcids and spiders can be drawn. Our results suggest frequent autosome–autosome and autosome–sex chromosome rearrangements during pholcid evolution. Such events have previously been attributed to the reproductive isolation of species. The peculiar X_1_X_2_Y system is probably ancestral for haplogynes. Chromosomes of the X_1_X_2_Y system differ considerably in their pattern of evolution. In some pholcid clades, the X_1_X_2_Y system has transformed into the X_1_X_2_0 or XY systems, and subsequently into the X0 system. The X_1_X_2_X_3_0 system of *Smeringopus pallidus* probably arose from the X_1_X_2_0 system by an X chromosome fission. The X_1_X_2_X_3_X_4_Y system of *Kambiwa* probably evolved from the X_1_X_2_Y system by integration of a chromosome pair. Nucleolus organizer regions have frequently expanded on sex chromosomes, most probably by ectopic recombination. Our data suggest the involvement of sex chromosome-linked NORs in achiasmatic pairing.

**Supplementary Information:**

The online version contains supplementary material available at 10.1186/s12862-021-01750-8.

## Background

The structure of chromosomes changes during evolution, just like any other genomic character. Fine-scale changes of the genome can be easily determined by techniques of DNA sequencing and assembling of sequences. However, long assemblies have gaps containing unknown sequences, which makes it difficult to perform linkage mapping of entire chromosomes. An important tool to complete genome assembly and understand transmission of specific loci are cytogenetic data [1]. Cytogenetics also brings other data on the genome and its dynamics, which are not easy to get by sequencing (e.g. mapping of highly differentiated sex chromosomes) [2, 3] or cannot be obtained by this approach (e.g. chromosome behaviour during nuclear division, or large-scale heterochromatin pattern).

Cytogenetic information is particularly important in the analysis of complex genomes, for example in spiders. Genome evolution of spider ancestors included a polyploid event [4], which might have been the origin of the unusual and complex spider sex chromosome determination. The male sex chromosome complement of most spiders includes several chromosomes that do not recombine during meiosis and are presumably non-homologous. Furthermore, it probably also contains a chromosome pair formed by the chromosomes X and Y, which recombine and show a very low level of differentiation (further cryptic sex chromosome pair, CSCP) [5]. Some mygalomorph spiders even exhibit two CSCPs [6]. The single CSCP could represent the ancestral sex chromosomes of spiders [7].

Despite recent progress in genomic analysis of spiders [8], their cytogenetics is not satisfactorily understood. Most data concern entelegyne araneomorphs. Although entelegynes exihibit an enormous species diversity (currently nearly 38,700 described species), their karyotypes are usually conservative, comprising a low number (2n♂ = 10–49) of exclusively monoarmed (i.e. acrocentric and subtelocentric) chromosomes and containing two different X chromosomes (♂X_1_X_2_/♀X_1_X_1_X_2_X_2_, the so-called X_1_X_2_0 system) [9]. The origin of these sex chromosomes is unresolved. The cytogenetics of the other spider clades (mesotheles, mygalomorphs, haplogyne araneomorphs) is far less studied.

**Fig. 1:**
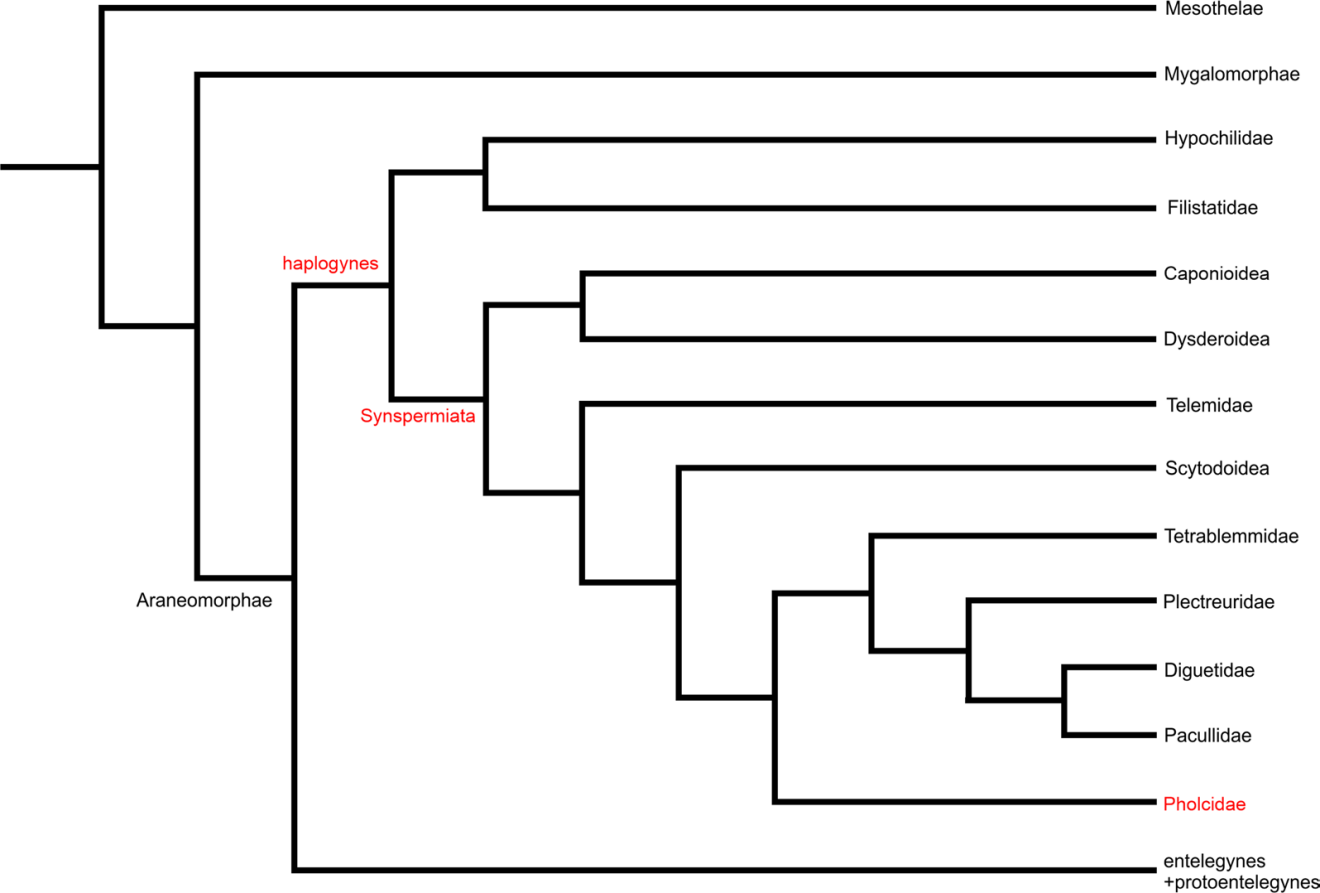
Phylogeny of spiders, with specific consideration on haplogyne spiders. The phylogenetic tree was constructed following [10] except for Telemidae [11]. Caponioidea: Caponiidae, Trogloraptoridae; Dysderoidea: Dysderidae, Oonopidae, Orsolobidae, Segestriidae; Scytodoidea: Drymusidae, Ochyroceratidae, Periegopidae, Psilodercidae, Scytodidae, Sicariidae

Recent phylogenomic analyses of spiders led to considerable changes in the taxonomic composition of haplogyne araneomorphs. Currently, haplogynes consist of the clade Synspermiata (18 families) and a clade formed by the families Hypochilidae and Filistatidae [10–12] (Fig. 1). These two clades currently include more than 6000 described species (based on the data of [13]). Although haplogynes have a much lower species diversity than entelegynes, their karyotypes are very diverse. The male diploid numbers of haplogynes vary from 5 to 152 [14]. According to chromosome structure, haplogynes show two principal patterns. A synapomorphy of the superfamily Dysderoidea is the holokinetic structure of chromosomes. Holokinetic chromosomes do not contain a localised centromere [14]. Karyotypes of all other studied clades are composed of standard (i.e. monokinetic) chromosomes, and are usually predominated by biarmed (i.e. metacentric and submetacentric) chromosomes [9]. Karyotypes of many haplogynes contain the peculiar X_1_X_2_Y system (♂X_1_X_2_Y/♀X_1_X_1_X_2_X_2_), a sex chromosome constitution that has been found in seven families [9, 14–17]. Chromosomes of the X_1_X_2_Y system usually exhibit a metacentric morphology. They pair without chiasmata and recombination (i.e. achiasmatically) during male meiosis. The phylogenetic distribution of the X_1_X_2_Y system suggests a considerable antiquity of this sex chromosome determination [9]. According to a recent hypothesis, this system could be ancestral for araneomorph spiders [16]. Despite this, the origin and subsequent evolutionary transformations of the X_1_X_2_Y system are not satisfactorily understood. Haplogynes also exhibit other unusual cytogenetic traits, namely a considerable decondensation of bivalents during a specific period of male prophase I (so-called diffuse stage) [9, 18] and the frequent occurrence of nucleolus organizer regions (NORs) on the sex chromosomes [9]. Nucleolus organizer regions are chromosome regions crucial for the formation of the nucleolus. They contain tandem copies of the genes coding 5.8S, 18S, and 28S ribosomal RNA [19].

Although fundamental trends of haplogyne karyotype evolution have been determined [9], karyotype evolution within haplogyne families remains unknown. To fill this gap, we have focused on the cytogenetics of the family Pholcidae (Fig. 1), which is an ideal model group for such a study. First, pholcids are the most diversified haplogyne family with a standard chromosome structure. They currently include 94 genera and 1812 species [13]. Second, pholcids are the spider family with the most comprehensive molecular phylogeny available (with 600 species representing 86% of the known genera [20, 21]). Third, they display a worldwide distribution, with the large majority of species in the tropics and subtropics. Numerous species are synanthropic and have been translocated by humans around the globe [22, 23].

Information on pholcid cytogenetics is very limited. There are only basic data available for 23 species representing nine genera (see database [24]). Here we present chromosome data of 47 species, which represent an extensive cross-section through all major pholcid clades. Since previous authors often failed in the proper determination of pholcid cytogenetic data, we have also revised previously published results. We further focused on the evolution of NORs, the evolution of which is largely unknown in spiders.

Our study represents one of the most comprehensive cytogenetic datasets of any spider family. Cytogenetic data of pholcids are diversified and have considerable potential to be used in the reconstruction of pholcid phylogeny. Based on our data, numerous conclusions and hypotheses regarding the karyotype evolution of pholcids, as well as haplogyne spiders, can be drawn. Finally, our results suggest promising subjects for future evolutionary studies on the karyotypes, sex chromosomes, and NORs of spiders.

## Results

### Arteminae

**Fig. 2:**
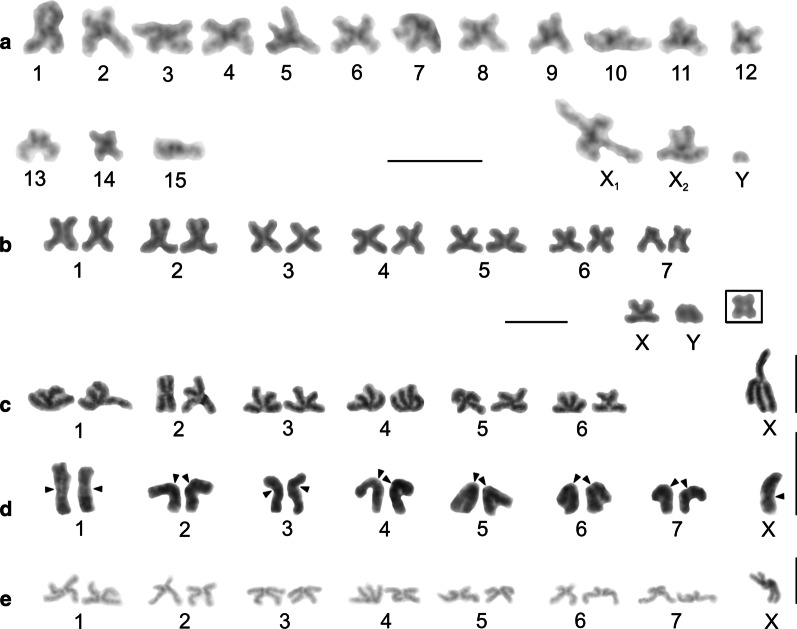
Arteminae, male karyotypes, stained by Giemsa. Based on two sister metaphases II except for *Artema nephilit* (single metaphase II) and *Holocneminus* (spermatogonial metaphase). **a** *Artema nephilit*, haploid karyotype. Chromosomes metacentric except for five submetacentric (nos. 2, 8, 10–12), one subtelocentric (no. 13), and one acrocentric element (no. 15). The Y chromosome is from another metaphase II. Morphology of the Y chromosome is unresolved; **b** *Wugigarra* sp. Karyotype metacentric except for one subtelocentric pair (no. 7). Inset: magnified Y chromosome (from another metaphase II). Note metacentric morphology of this element; **c** *Chisosa diluta*. Chromosomes exclusively metacentric. The positively heteropycnotic X chromosome is the longest chromosome; **d** *Holocneminus* sp. Chromosomes metacentric except for one submetacentric (no. 4) and one subtelocentric pair (no. 3). Centromeres marked by arrowheads; **e** *Physocyclus dugesi*. Chromosomes metacentric except for two submetacentric (nos 2, 5) and two subtelocentric pairs (nos 6, 7). The positively heteropycnotic X chromosome is the longest chromosome of the karyotype. Bar = 10 µm

Our study included representatives of the genera *Artema*, *Chisosa*, *Holocneminus*, *Physocyclus*, and *Wugigarra* (Additional file 1: Table S1, Additional file 2: Table S2). The diploid number in artemine males ranged from 2n = 13 (*Chisosa diluta*) to 2n = 33 (*Artema* spp.), with most species having 2n = 15. In all species, the karyotypes consisted predominantly of biarmed chromosomes (Additional file 2: Table S2, Fig. 2, Additional file 3: Fig. S1).

Representatives of *Artema* differed from the other artemines by a much higher diploid number and a X_1_X_2_Y system. The male karyotype of *Artema* contained 15 chromosome pairs (this term is used in a specific context in our study, see “Methods”, p. 31) decreasing gradually in size (Additional file 1: Table S1, Additional file 2: Table S2, Additional file 4: Fig. S2). Chromosome morphology was predominantly metacentric. The X_1_ chromosome was considerably longer than X_2_. The Y chromosome was the smallest element of the karyotype (Additional file 1: Table S1, Additional file 2: Table S2, Fig. 2a, Additional file 3: Fig. S1).

Karyotypes of the other artemines contained only six (*C. diluta*) or seven chromosome pairs (the other species). Chromosome pairs decreased gradually in size except for the prominent longest pair of *C. diluta* (Additional file 1: Table S1). The karyotype of *Wugigarra* (2n♂ = 16) was characterized by a XY system (Fig. 2b, Additional file 4: Fig. S2). The Y chromosome was the smallest element of the complement, a feature similar to *Artema* (Additional file 1: Table S1). The other taxa exhibited the X0 system (Fig. 2c–e, Additional file 5: Fig. S3). Their sex chromosome was the longest element of the karyotype except for *Holocneminus* sp. The size of the X chromosome varied considerably from 11.4% (*Holocneminus* sp.) to 21.2% of TCL (*C. diluta*). The length of the sex chromosome was determined from mitotic metaphase (*Holocneminus*) or metaphase II (the other X0 species). The small size of the X chromosome in *Holocneminus* compared to the other species could then reflect the fact that this element is more condensed in mitotic metaphase than in metaphase II*.*

**Fig. 3:**
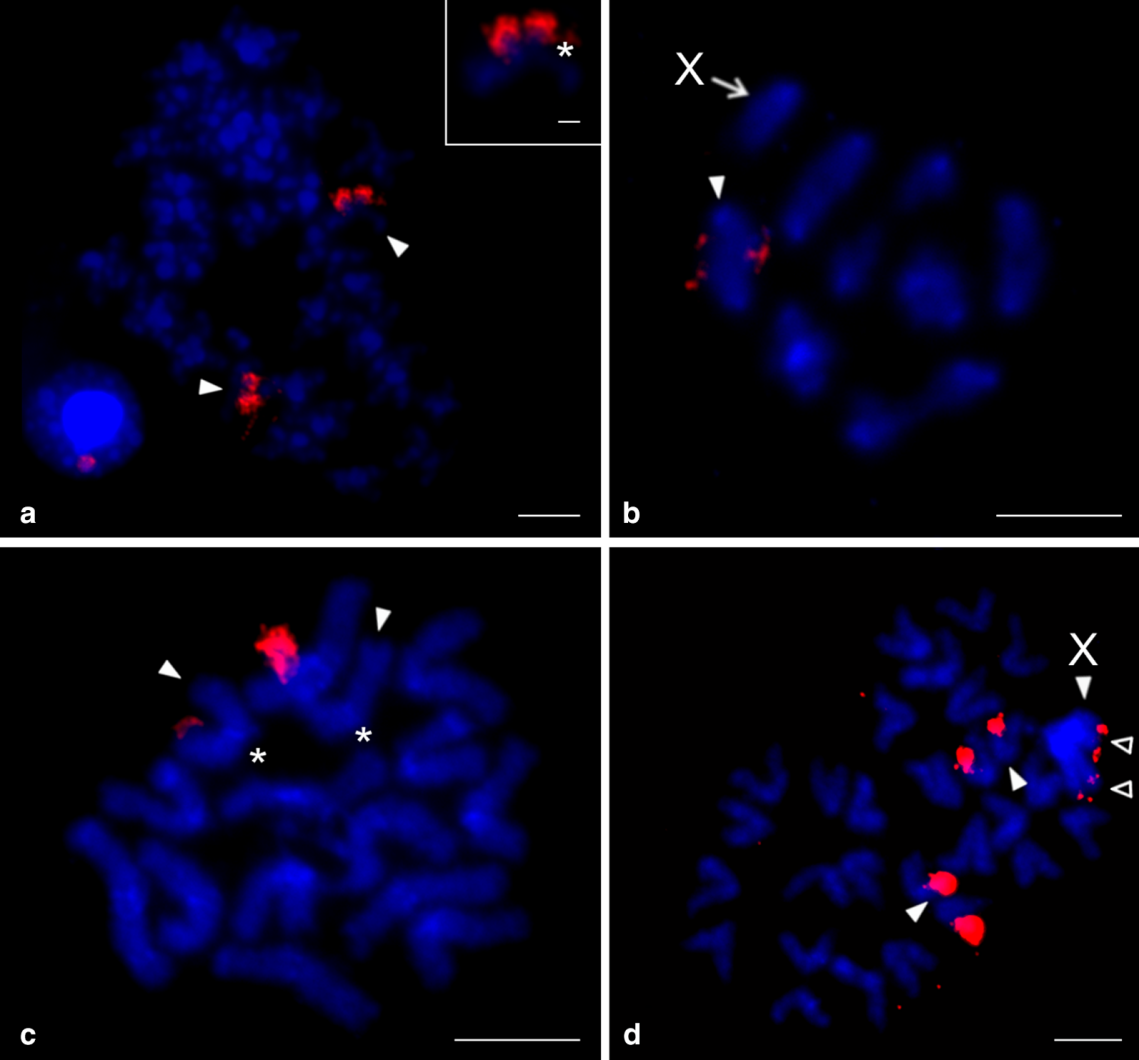
Arteminae, males, detection of NORs (FISH). *Arrowhead* = NOR-bearing chromosome (**a**, **c**, **d**) or bivalent (**b**), *asterisk* = centromere, *open arrowhead* = sex chrosomosome-linked NOR, X = X chromosome. **a** *Artema nephilit* (X_1_X_2_Y), plate formed by fused sister metaphases II. Pair of chromosomes bears a terminal NOR. Inset: submetacentric NOR-bearing chromosome. NOR is placed at the end of short arm; **b**, **c** *Holocneminus* sp. (X0). **b** Metaphase I, one bivalent contains NOR; **c** Mitotic metaphase. Chromosomes of one metacentric pair have terminal NOR; **d** *Physocyclus dugesi* (X0), two sister plates, late metaphase II. Chromosomes of one metacentric/submetacentric pair contain a terminal NOR. X chromosome is more condensed than the other chromosomes. It includes tiny NOR at both ends. Bar = 5 µm, inset 1 µm

The nucleolus organizer region was present at an end of one chromosome pair in all of the studied species (Additional file 2: Table S2, Fig. 3, Additional file 6: Fig. S4), and in *Physocyclus* also on both termini of the X chromosome (Fig. 3d). The morphology of the NOR-bearing pair was metacentric in *Holocneminus* (Fig. 3c), metacentric/submetacentric in *Physocyclus* (Fig. 3d), submetacentric in *Artema* (Fig. 3a, Additional file 6: Fig. S4a, b), and subtelocentric in *Wugigarra* (Additional file 6: Fig. S4c). Except for *Holocneminus*, the NOR was placed at the end of the short arm.

### Modisiminae

**Fig. 4:**
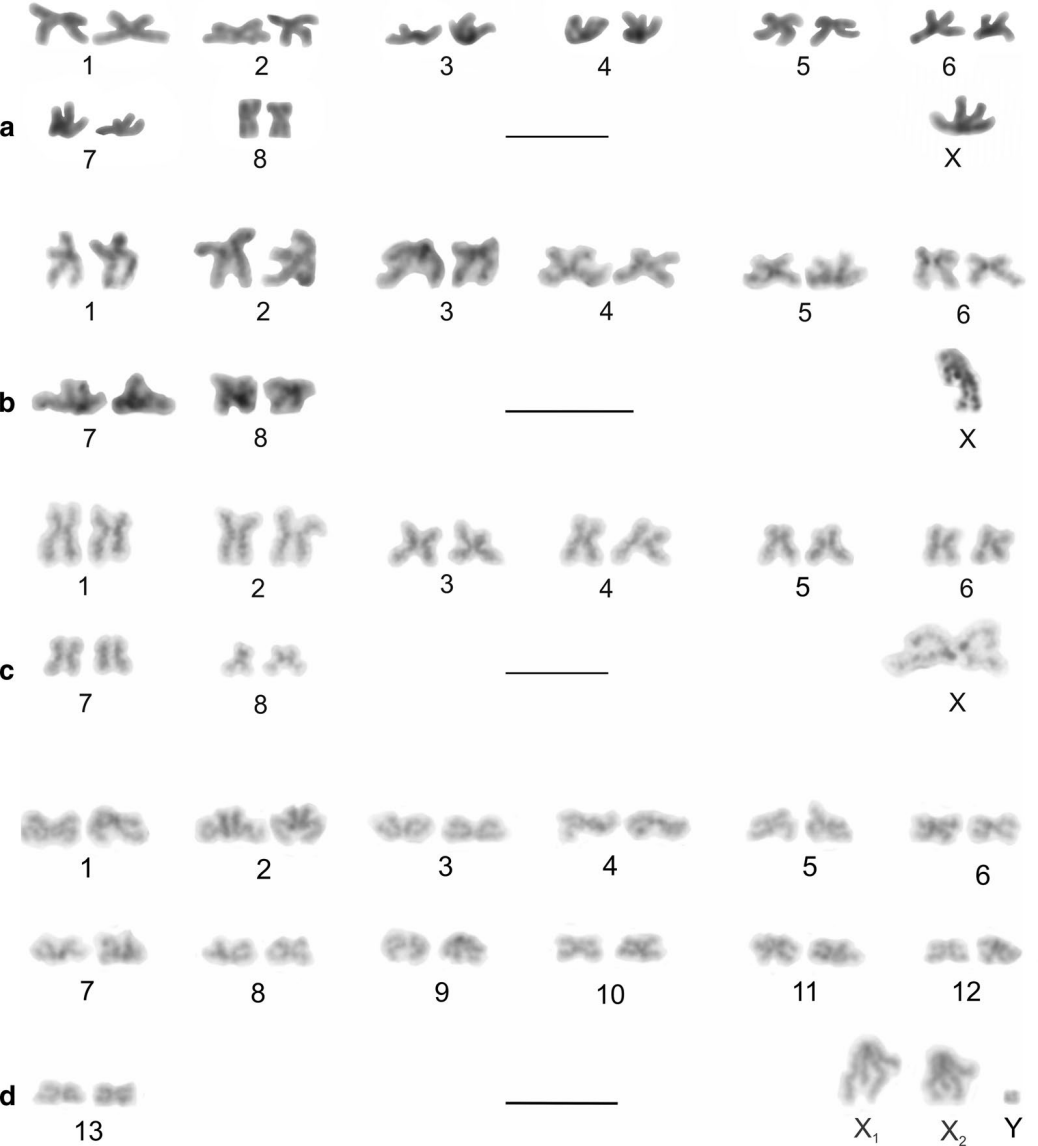
Modisiminae (**a**–**c**) and Ninetinae (**d**), male karyotypes, stained by Giemsa. Based on two sister metaphases II. Karyotypes are predominated by metacentrics. Single sex chromosome of modisimines is the longest element of the karyotype. **a** *Anopsicus* sp., pair nos. 3 and 5 are submetacentric; **b** *Modisimus* cf. *elongatus*. Three pairs submetacentric (nos 1, 3, 6), X chromosome positively heteropycnotic; **c** *Psilochorus simoni*. Chromosome pairs form three size groups (pairs 1–2, 3–7, 8). Two pairs submetacentric (nos 1, 5). Sex chromosome negatively heteropycnotic; **d** *Pholcophora americana*, pair no. 2 submetacentric. Bar = 10 µm

We analysed representatives of *Anopsicus*, *Modisimus*, and *Psilochorus* (Additional file 7: Table S3). The studied modisimines exhibited the same male diploid number (17), sex chromosome system (SCS) (X0), and a metacentric sex chromosome (Additional file 1: Table S1, Additional file 7: Table S3, Fig. 4a–c, Additional file 8: Fig. S5, Additional file 9: Fig. S6). Chromosomes of modisimines were biarmed except for one pair of *P*. *californiae* (Additional file 1: Table S1, Additional file 7: Table S3). The sex chromosome was the longest chromosome of the karyotype (Additional file 1: Table S1).

**Fig. 5:**
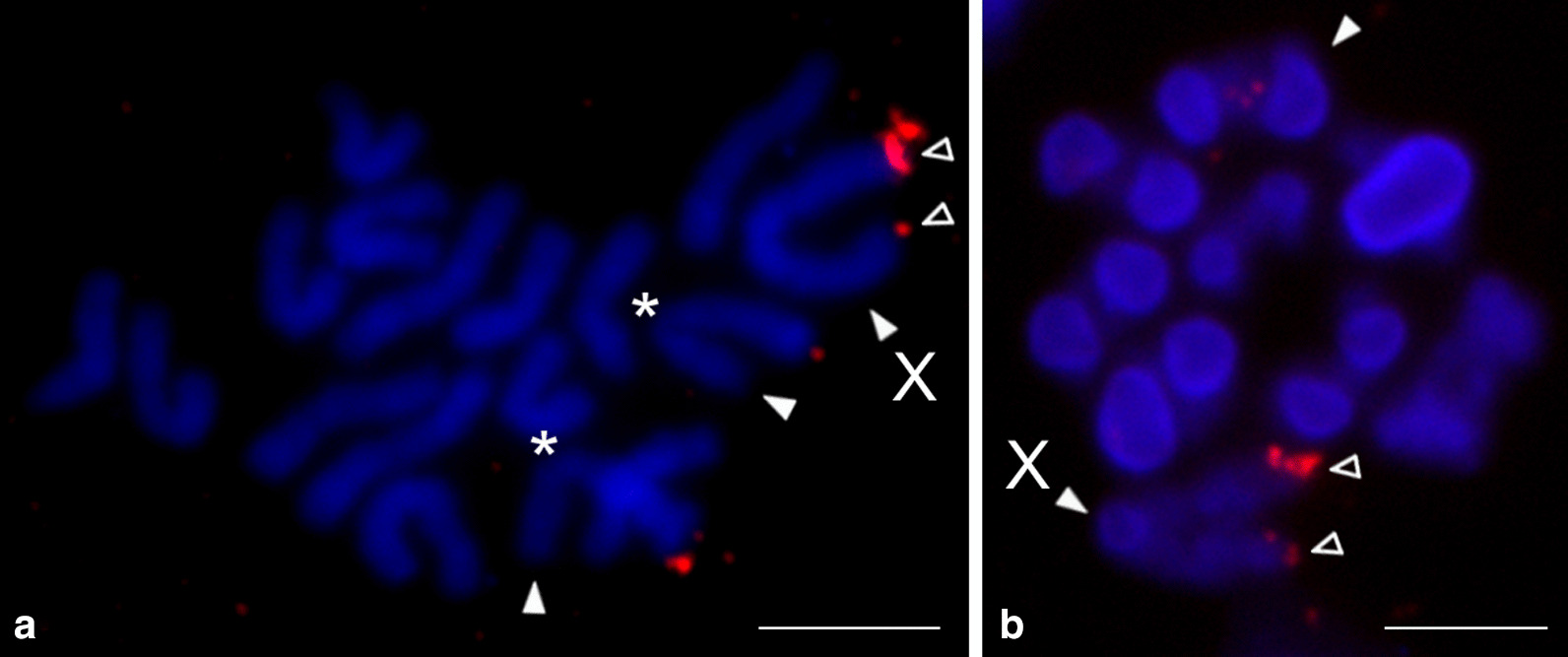
Modisiminae, males, detection of NORs (FISH). *Arrowhead* = NOR bearing chromosome (**a**) or bivalent (**b**), *asterisk* = centromere, *open arrowhead* = sex chromosome-linked NOR, X=X chromosome. **a** *Psilochorus californiae* (X0), mitotic metaphase. Chromosomes of one metacentric pair contain a terminal NOR. X chromosome bears NOR at both ends; **b** *P. pallidulus* (X0), metaphase I. One bivalent includes a tiny NOR. X chromosome has two terminal NORs. Bar = 5 µm

The genus *Psilochorus* was represented by three species. Chromosome pairs of *P. californiae* decreased gradually in size. In contrast, the longest pair of *P. pallidulus* and the two longest pairs of *P. simoni* were prominent. Moreover, in the latter two species the last pair was much smaller than the penultimate pair (Additional file 1: Table S1). The karyotype of *P. pallidulus* was formed exclusively by metacentric chromosomes (Additional file 9: Fig. S6b). The karyotypes of the other two species were also formed by metacentrics except for two submetacentric pairs of *P. simoni*, and two submetacentric and one subtelocentric pair of *P. californiae* (Fig. 4c, Additional file 9: Fig. S6a). Nucleolus organizer regions were detected in *P. californiae* and *P. pallidulus*. Their karyotype included a NOR-bearing metacentric pair. While the NOR of *P. californiae* exhibited a terminal location, placement of NOR in *P. pallidulus* was not determined. In both species, the sex chromosome bore NORs at both ends (Fig. 5).

### Ninetinae

**Fig. 6:**
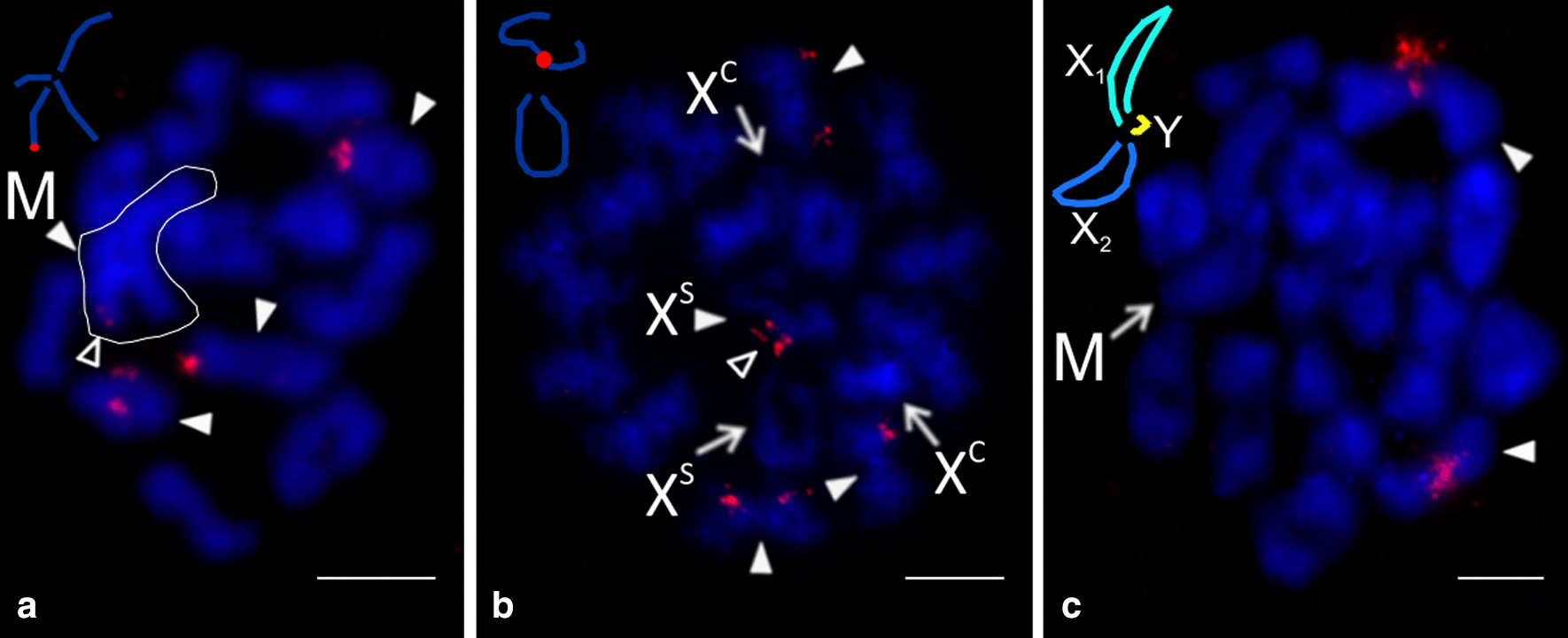
Ninetinae, males, detection of NORs (FISH). Figures contain scheme of sex chromosomes (red = NOR). *Arrowhead* = chromosome (**b**), bivalent (**a**, **c**) or multivalent (**a**) with NOR, M = sex chromosome multivalent, *open arrowhead* = sex chromosome-linked NOR, X = X chromosome. **a**, **b** *Kambiwa neotropica* (X_1_X_2_X_3_X_4_Y) **a** Metaphase I, three bivalents contain terminal NOR. Moreover, sex chromosome multivalent (delimited by line) includes a tiny NOR at end of one “arm”. Note scheme for morphology of multivalent. **b** Metaphase II (n = 16) containing four X chromosomes. Two X chromosomes are the most condensed elements of the plate (X^C^). Two other X chromosomes have lower condensation and monoarmed morphology (X^s^). Note scheme for morphology of monoarmed X chromosomes. One monoarmed X contains a NOR at short arm. In addition, three other chromosomes bear a NOR; **c** *Pholcophora americana* (X_1_X_2_Y)*,* metaphase I. Two large bivalents contain terminal NOR. Note scheme for probable morphology of sex chromosome multivalent. Arms of each X chromosome are associated with each other. Bar = 5 µm

We studied representatives of *Kambiwa* and *Pholcophora* (Additional file 7: Table S3, Additional file 10: Fig. S7)*.* The chromosomes of *K. neotropica* were metacentric except for two monoarmed elements (Additional file 11: Fig. S8a). Several lines of observations demonstrated a complicated sex chromosome system in *Kambiwa*. Sex chromosomes formed a peculiar cross-shaped element in male metaphase I (Additional file 10: Fig. S7a, b). Comparison of data on male 2n (2n♂ = 29, Additional file 11: Fig. S8a) and male metaphase I (12 bivalents plus cross-shaped element) (Additional file 10: Fig. S7a, b) suggested that the cross-shaped element is a multivalent comprising of five chromosomes. Analysis of male metaphases II demonstrated the presence of a X_1_X_2_X_3_X_4_Y system. There are two types of metaphase II plates, one with 13 chromosomes including the Y microchromosome, and another one with 16 chromosomes including four heterochromatic X (Fig. 6b, Additional file 11: Fig. S8b, c). Two X chromosomes differed from the remaining two by a specific condensation at metaphase II. These chromosomes were monoarmed (Figs. 6b). The morphology of the tiny Y chromosome was unresolved. Chromosomes of *Pholcophora americana* (2n♂ = 29, X_1_X_2_Y) decreased gradually in size except for the prominent longest pair (Additional file 1: Table S1). They were metacentric except for one submetacentric pair (Fig. 4d). The X chromosomes showed a similar length. While they were the longest elements of the karyotype, the Y chromosome was the smallest one (Additional file 1: Table S1).

Three chromosome pairs of *Kambiwa* contained a terminal NOR (Fig. 6a). Moreover, one monoarmed X chromosome bore a NOR on its short arm (Fig. 6b). *Pholcophora* exhibited two chromosome pairs bearing terminal NORs (Fig. 6c).

### Pholcinae

We analysed pholcines representing twelve genera (Additional file 12: Table S4). Male 2n ranged from 9 (*Micropholcus fauroti*) to 25 (*Nipisa deelemanae*, three *Pholcus* species, *Spermophora senoculata*) (Additional file 12: Table S4). Chromosome morphology was determined in representatives of eight genera (Additional file 1: Table S1). Chromosome pairs of most pholcines decreased gradually in size. In several species, however, the longest pair(s) were prominent (first pair of *Belisana sabah* and *Pholcus opilionoides*; first three pairs of *Leptopholcus guineensis*; first five pairs in *Pholcus* sp.). In *Aetana kinabalu* the last chromosome pair was much smaller than the penultimate pair (Additional file 1: Table S1). Chromosome pairs were biarmed, except for two subtelocentric pairs in *Quamtana filmeri*, one subtelocentric pair in *Pholcus kindia*, and one acrocentric pair in *A. kinabalu* and *Pholcus* sp. (Additional file 12: Table S4).

**Fig. 7:**
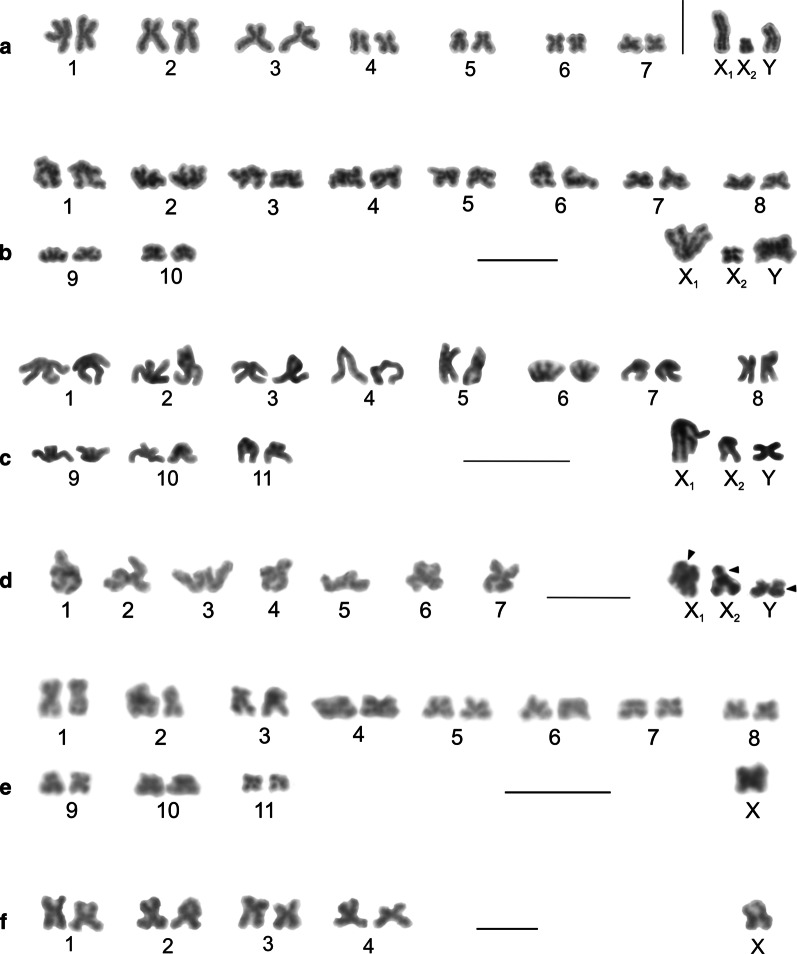
Pholcinae, male karyotypes, Giemsa staining. Based on two sister metaphases II (**a**–**c**, **e**, **f**) or metaphase II (**d**). **a** *Leptopholcus guineensis*. Chromosome pairs form two size groups, large-sized (nos 1–3) and small-sized (nos 4–7). The X_2_ chromosome replaced by X_2_ from another plate. The X_2_ and Y chromosome as well as three pairs (nos 2, 3, 5) submetacentric. Sex chromosomes positively heteropycnotic; **b** *Pholcus kindia*. Chromosomes biarmed except for one subtelocentric pair (no. 6). Chromosomes X_1_ and Y longest elements of karyotype, chromosomes X_2_ and Y positively heteropycnotic; **c** *Pholcus* sp. Chromosome pairs form two size groups, large-sized (nos 1–5) and small-sized (nos 6–11). Chromosomes metacentric except for three submetacentric (nos 7, 9, 11) and one acrocentric pairs (no. 4), and subtelocentric X_2_ chromosome. Y chromosome positively heteropycnotic; **d** *Quamtana filmeri*, haploid karyotype. Chromosomes metacentric except for one submetacentric (no. 4) and two subtelocentric pairs (nos 3, 5), and subtelocentric X_2_ chromosome. Y chromosome replaced by Y from another plate. Sex chromosomes positively heteropycnotic. Centromeres of sex chromosomes marked by arrowheads; **e** *Belisana sabah*. Karyotype metacentric except for one submetacentric pair (no. 3). First pair considerably longer than the other pairs, which gradually decrease in size. Third and eleventh pairs replaced by corresponding pairs from another plate. X chromosome positively heteropycnotic; **f** *Micropholcus fauroti*, chromosomes metacentric. Bar = 10 µm

Concerning sex chromosomes, most pholcines showed the X_1_X_2_Y system, which was found in nine genera (*Aetana*, *Leptopholcus*, *Metagonia, Muruta*, *Nipisa*, *Pehrforsskalia, Pholcus*, *Quamtana*, *Spermophora*) (Additional file 1: Table S1, Fig. 7a–d, Additional file 13: Fig. S9, Additional file 14: Fig. S10, Additional file 15: Fig. S11, Additional file 16: Fig. S12). The X_1_ chromosome was usually the largest element of the karyotype, the X_2_ chromosome a small to medium element, and the Y chromosome usually a microchromosome or small element (Additional file 1: Table S1). While the X_1_ and X_2_ chromosomes of *Aetana* and *Spermophora* were medium-sized elements of similar size, the Y chromosome of these species was a microchromosome (Additional file 1: Table S1). Although there are no precise data on the morphology of the sex chromosomes in *Metagonia*, they probably also consist of X chromosomes of similar size and a tiny Y chromosome (Additional file 11: Fig. S8f, Additional file 14: Fig. S10e). Most other pholcines exhibiting the X_1_X_2_Y system showed a X_2_ size reduction (up to 2.7% of TCL in *Pholcus pagbilao*) and a considerable increase of the Y chromosome size (up to 11.7% of TCL in *P. kindia*). In some *Pholcus* and *Quamtana* species, the X_2_ chromosome was only slightly larger than the Y chromosome (Additional file 1: Table S1). In *Leptopholcus* and two *Pholcus* species (*P. kindia*, *P. pagbilao*), the Y chromosome was even longer than the X_2_ chromosome (Additional file 1: Table S1, Fig. 7a, b, Additional file 16: Fig. S12b). The X_1_ chromosome of pholcines was metacentric, except for *Aetana*, whose X_1_ chromosome was submetacentric (Additional file 12: Table S4, Additional file 16: Fig. S12a). In contrast, the morphology of the X_2_ was variable. It was metacentric in *Aetana*, *Nipisa*, *Spermophora*, and African *Pholcus* species (Fig. 7b, Additional file 11: Fig. S8i, Additional file 12: Table S4, Additional file 13: Fig. S9f, Additional file 16: Fig. S12a), submetacentric in *Leptopholcus*, *Muruta*, and *Pholcus phalangioides* (Additional file 12: Table S4, Fig. 7a, Additional file 15: Fig. S11b), subtelocentric in *Quamtana filmeri* and *Pholcus* sp. (Fig. 7c, d, Additional file 12: Table S4), and acrocentric in *Quamtana hectori*, *Pholcus opilionoides* and *P. pagbilao* (Additional file 12: Table S4, Additional file 16: Fig. S12b–d). The Y chromosome exhibited a metacentric morphology, except for *Leptopholcus* and *P. pagbilao*, whose Y chromosome was submetacentric (Fig. 7a, Additional file 12: Table S4, Additional file 16: Fig. S12b). The morphology of the Y chromosome of *Aetana*, *Muruta*, and *Nipisa*, as well as sex chromosomes of *Metagonia* and *Pehrforsskalia*, were not determined.

**Fig. 8:**
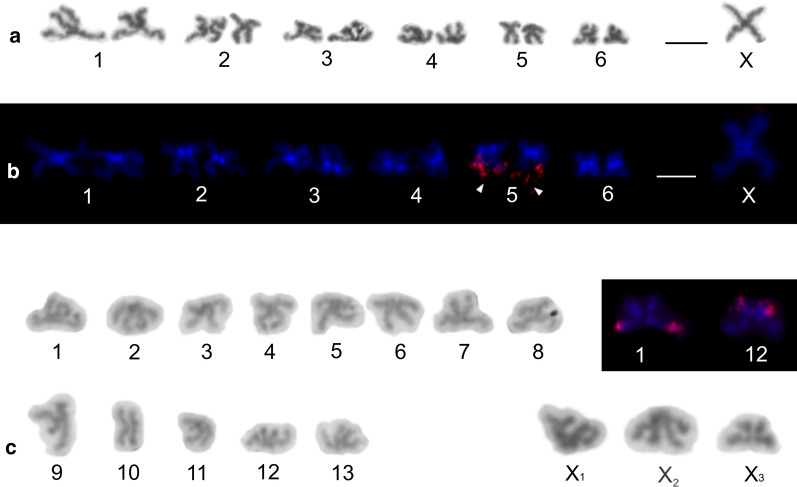
Male karyotype of *Cantikus sabah* (Pholcinae) (**a, b**) and *Smeringopus pallidus* (Smeringopinae) (**c**). Based on metaphase II (**c**) or two sister metaphases II (**a**, **b**). *Arrowhead* = NOR. **a** Diploid karyotype, Giemsa stained. Note metacentric morphology of chromosomes. **b** Diploid karyotype, detection of NORs (FISH). Fifth pair bears a terminal NOR (arrowheads). In contrast to most other pholcids, centromeres are formed by a prominent AT-rich block; **c** Haploid karyotype, Giemsa stained. Chromosomes metacentric except for three submetacentrics (nos 5, 9, 12). Sex chromosomes show a slight positive heteropycnosis. Inset: detection of NORs (FISH); chromosomes of two pairs (nos. 1, 12) contain terminal NOR. Bar = 10 µm

Some pholcines displayed the X0 system (*Belisana*, *Cantikus*, and *Micropholcus*). The X chromosome was metacentric (Additional file 12: Table S4, Figs. 7e, f, 8a, b, Additional file 17: Fig. S13). Its size was comparable to the size of short (*Micropholcus*) or medium-sized chromosome pairs (*Belisana*, *Cantikus*) (Additional file 1: Table S1).

**Fig. 9:**
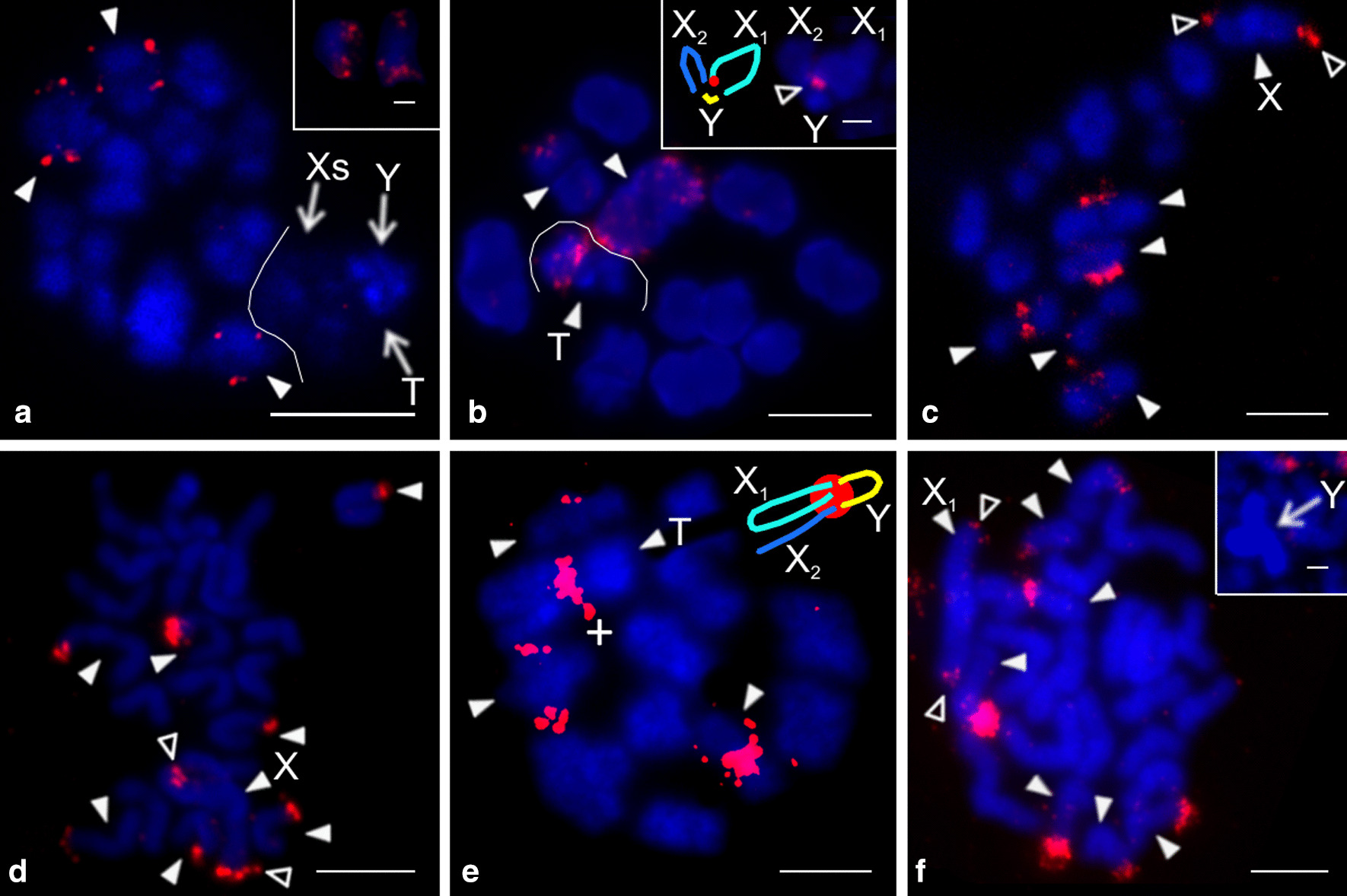
Pholcinae, males, NORs (FISH) I. Figures **b**, **e** contain scheme of sex chromosome trivalent X_1_X_2_Y (red circle at **c** = signal formed by several NORs). *Arrowhead* = NOR bearing chromosome (**c**, **d**, **f**), bivalent (**a**–**c**, **e**) or trivalent (**b**, **e**), *open arrowhead* = sex chromosome-linked NOR, T = sex chromosome trivalent (**a**, **b**, **e**), X = X chromosome, X_1_ = X_1_ chromosome, X_2_ = X_2_ chromosome, Xs = X chromosomes, Y = Y chromosome, +  = signal formed by several NORs. **a** *Spermophora senoculata* (X_1_X_2_Y), diakinesis, note three NOR-bearing bivalents, sex chromosome trivalent separated by line (X chromosomes considerably decondensed). Inset: mitotic metaphase, chromosome pair bearing two terminal NOR loci; **b** *Aetana kinabalu* (X_1_X_2_Y), metaphase I, the largest bivalent and one medium-sized bivalent bear a terminal NOR. The sex chromosome trivalent (separated by line) also includes NOR signal. Position of NOR is, however, unclear. Inset: diplotene, sex chromosome trivalent and its scheme, note NOR at the end of X_1_ chromosome; **c**, **d** *Belisana sabah* (X0), **c** Metaphase I. Five bivalents contain NOR, sex chromosome with NOR at both ends. **d** Incomplete mitotic metaphase (21 chromosomes), NORs terminal, X chromosome with NOR at both ends; **e**, **f** *Pholcus phalangioides* (X_1_X_2_Y)*.* **e** Metaphase I, three bivalents contain NOR. The sex chromosome trivalent contains the signal in region of chromosome pairing (see scheme). **f** Mitotic metaphase, NORs terminal. X_1_ chromosome bears NOR at both ends. Number of the other NOR-bearing chromosomes (seven) suggests that the X_2_ chromosome contains a NOR too. Inset: another mitotic metaphase, Y chromosome considerably condensed, without NOR. Bar = 5 µm; insets 1 µm

**Fig. 10:**
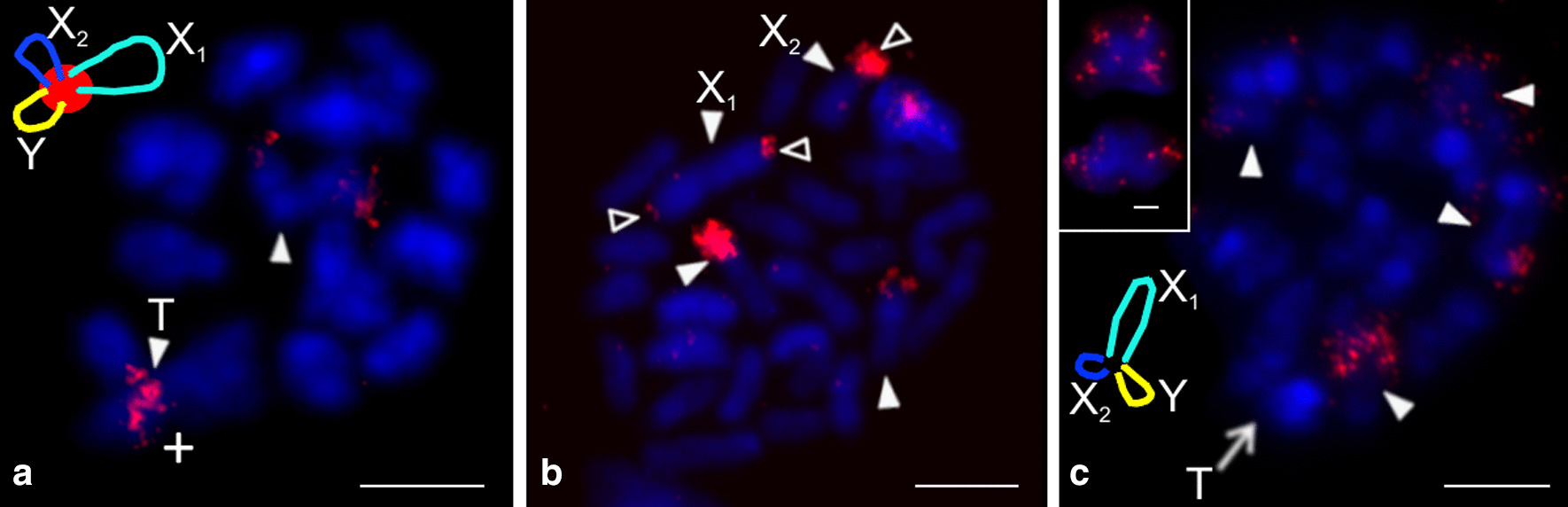
Pholcinae, males, NORs (FISH) II. Figures **a**, **c** contain scheme of sex chromosome trivalent X_1_X_2_Y (red circle = signal formed by several NORs). *Arrowhead* = NOR bearing chromosome (**b**), bivalent (**a**, **c**) or trivalent (**a**), *open arrowhead* = sex chromosome-linked NOR, T = sex chromosome trivalent X_1_ = X_1_ chromosome, X_2_ = X_2_ chromosome, Y = Y chromosome, +  = signal formed by several NORs. **a**, **b** *Muruta tambunan* (X_1_X_2_Y)*.* **a** Metaphase I, note a bivalent bearing a terminal NOR and sex chromosome trivalent with signal in region of chromosome pairing (see scheme). **b** Mitotic metaphase, the large X_1_ chromosome bears two terminal NORs, the X_2_ chromosome contains one terminal NOR. Chromosomes of a chromosome pair exhibit another terminal NOR; **c** *Pholcus pagbilao* (X_1_X_2_Y), metaphase I. Plate contains four NOR bearing bivalents and sex chromosome trivalent without signal (see scheme). Inset: metaphase II, NOR bearing pair, upper chromosome contains two terminal loci. Bar = 5 µm; inset 1 µm

Nucleolus organizer regions were detected in representatives of nine genera, namely *Aetana*, *Belisana*, *Cantikus*, *Micropholcus*, *Muruta*, *Nipisa*, *Pholcus*, *Quamtana*, and *Spermophora* (Additional file 12: Table S4). Pholcines showed a considerable diversity of NOR patterns. Their karyotypes contained from one (*Cantikus*, *Micropholcus*, *Muruta*, *Q. filmeri*) to five NOR-bearing chromosome pairs (*Belisana*) (Figs. 8b, 9, 10, Additional file 18: Fig. S14). In most species, we also determined the morphology of these pairs, including the NOR location. Pairs bearing NORs were biarmed; NORs were placed at chromosome ends (Additional file 12: Table S4). All pairs included one NOR locus except for one pair of *Nipisa*, *P. pagbilao*, and *Spermophora*, which had a locus at both ends (Figs. 9a, 10c, Additional file 18: Fig. S14b). In *Spermophora*, both loci of this pair were homozygous for the presence of a NOR (Fig. 9a). In the studied male of *P. pagbilao*, one of these loci was heterozygous for the presence of a NOR (Fig. 10c). Concerning the studied *Nipisa* male, comparison of the NOR pattern of the chromosome pairs in metaphase I (three NOR bearing pairs, Additional file 18: Fig. S14c) and mitotic metaphase (five chromosomes with NOR, including one chromosome with NOR at both ends, Additional file 18: Fig. S14b) implied that one chromosome pair consists of a chromosome with NOR at both ends plus a chromosome with one NOR or without NOR. It means that one or even both loci of this pair were heterozygous for the presence of a NOR.

Sex chromosome-linked NORs were revealed in *Aetana*, *Muruta*, *Nipisa, Quamtana*, *Pholcus phalangioides* (X_1_X_2_Y), and *Belisana* (X0) (Additional file 12: Table S4, Figs. 9b–f, 10a, b, Additional file 18: Fig. S14b–h). In *Aetana*, the X_1_ chromosome had a terminal NOR (Fig. 9b). In *Muruta, Nipisa*, *Quamtana*, and *P. phalangioides*, NORs were detected at both ends of the X_1_ chromosomes (Additional file 12: Table S4, Figs. 9f, 10b, Additional file 18: Fig. S14b, d, f–h). Furthermore, the X_2_ chromosome of some pholcines involved NOR(s) too. While the X_2_ chromosome of *Muruta* and *P. phalangioides* exhibited NOR at one end only (Figs. 9f, 10b), the X_2_ chromosome of *Nipisa* was terminated by NOR at both ends (Additional file 18: Fig. S14b). In the latter pholcid, the Y chromosome also had a terminal NOR (Additional file 18: Fig. S14b). In *Belisana*, a NOR was detected at both ends of the X chromosome (Fig. 9c, d). In species exhibiting the X_1_X_2_Y system, the sex chromosome ends bearing a NOR took part in sex chromosome pairing during male meiosis (Figs. 9b, e, 10a, Additional file 18: Fig. S14c, e).

### Smeringopinae

**Fig. 11:**
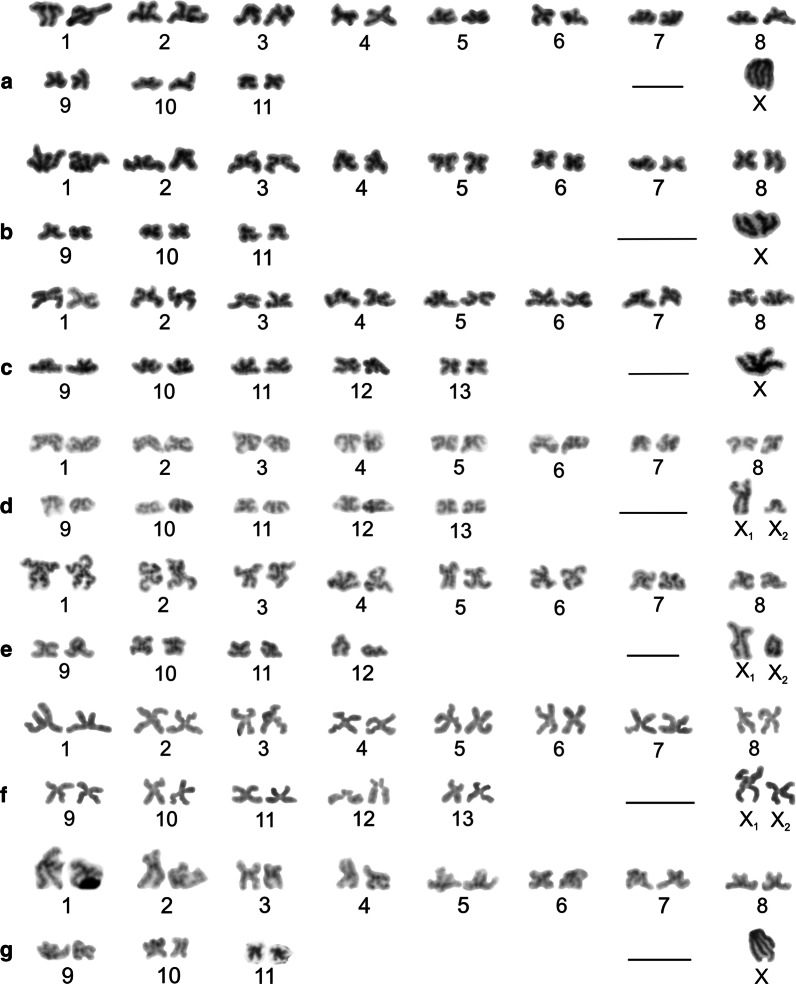
Smeringopinae, male karyotypes, stained by Giemsa. Based on two sister metaphases II. Chromosomes are mostly metacentric. Sex chromosomes of some species are positively heteropycnotic during metaphase II (**b**,** c**,** f**,** g**). Chromosome X (or X_1_) is the longest element of the karyotype except for **a** and **e**. **a** *Crossopriza* sp., four pairs submetacentric (nos 1, 3, 8, 10); **b** *Holocnemus hispanicus*, three submetacentric (nos 2–4) and one subtelocentric pairs (no. 11); **c** *H. pluchei*, four pairs submetacentric (nos 2, 5, 8, 9); **d** *Hoplopholcus cecconii*, chromosomes metacentric except for one submetacentric pair (no. 6) and subtelocentric X_2_; **e** *Smeringopus cylindrogaster*, one submetacentric pair (no. 5), one acrocentric pair (no. 12), and acrocentric X_2_; **f** *S. similis*, two pairs submetacentric (nos 3, 13) and one pair subtelocentric (no. 12); **g** *Stygopholcus skotophilus*, three submetacentric (nos 2, 3, 7) and one subtelocentric pair (no. 11). Last pair replaced by corresponding pair from another plate. Bar = 10 µm

We analysed representatives of *Crossopriza*, *Holocnemus*, *Hoplopholcus*, *Smeringopus*, and *Stygopholcus* (Additional file 19: Table S5). The male 2n ranged from 23 to 29 (Additional file 19: Table S5, Figs. 8c, 11, Additional file 20: Fig. S15, Additional file 21: Fig. S16). Chromosome pairs of most smeringopines decreased gradually in length. In some species the longest pair (*Holocnemus*, *Smeringopus cylindrogaster*) or two longest pairs (*Crossopriza lyoni*, *Stygopholcus skotophilus*) were prominent. Furthermore, the last pair of *S. skotophilus* was much smaller than the penultimate pair. Chromosome pairs were exclusively biarmed except for *Holocnemus hispanicus*, *S. skotophilus*, and some *Hoplopholcus* and *Smeringopus* species, whose karyotype included one monoarmed pair (Additional file 1: Table S1).

The male karyotype of *Hoplopholcus* and *Smeringopus* was composed of 28 chromosomes, including the X_1_X_2_0 system (Additional file 19: Table S5, Fig. 11d, f, Additional file 20: Fig. S15, Additional file 22: Fig. S17a–e), except for *S. pallidus* (2n♂ = 29, X_1_X_2_X_3_0) (Fig. 8c, Additional file 22: Fig. S17f) and *S. cylindrogaster* (2n♂ = 26, X_1_X_2_0) (Fig. 11e). The metacentric X_1_ chromosome was the largest element of the karyotype, except for three *Smeringopus* species. The X_2_ chromosome was usually a medium-sized element (Additional file 1: Table S1). It had a variable morphology, being metacentric in *S. ndumo*, *S. pallidus* and *S. similis*, submetacentric in *S. atomarius*, subtelocentric in *Hoplopholcus* and *S. peregrinus*, and acrocentric in *S. cylindrogaster* and *Smeringopus* sp. (Additional file 1: Table S1). The metacentric X_3_ chromosome of *S. pallidus* was the smallest chromosome of the karyotype (Additonal file 1: Table S1).

Representatives of the genera *Crossopriza*, *Holocnemus*, and *Stygopholcus* exhibited the X0 system, including a metacentric X chromosome (Additional file 19: Table S5, Fig. 11a–c, g, Additional file 19: Fig. S16, Additional file 23: Fig. S18), which was the largest element of the set except for *Crossopriza* sp. and *Holocnemus caudatus* (Additional file 1: Table S1).

**Fig. 12:**
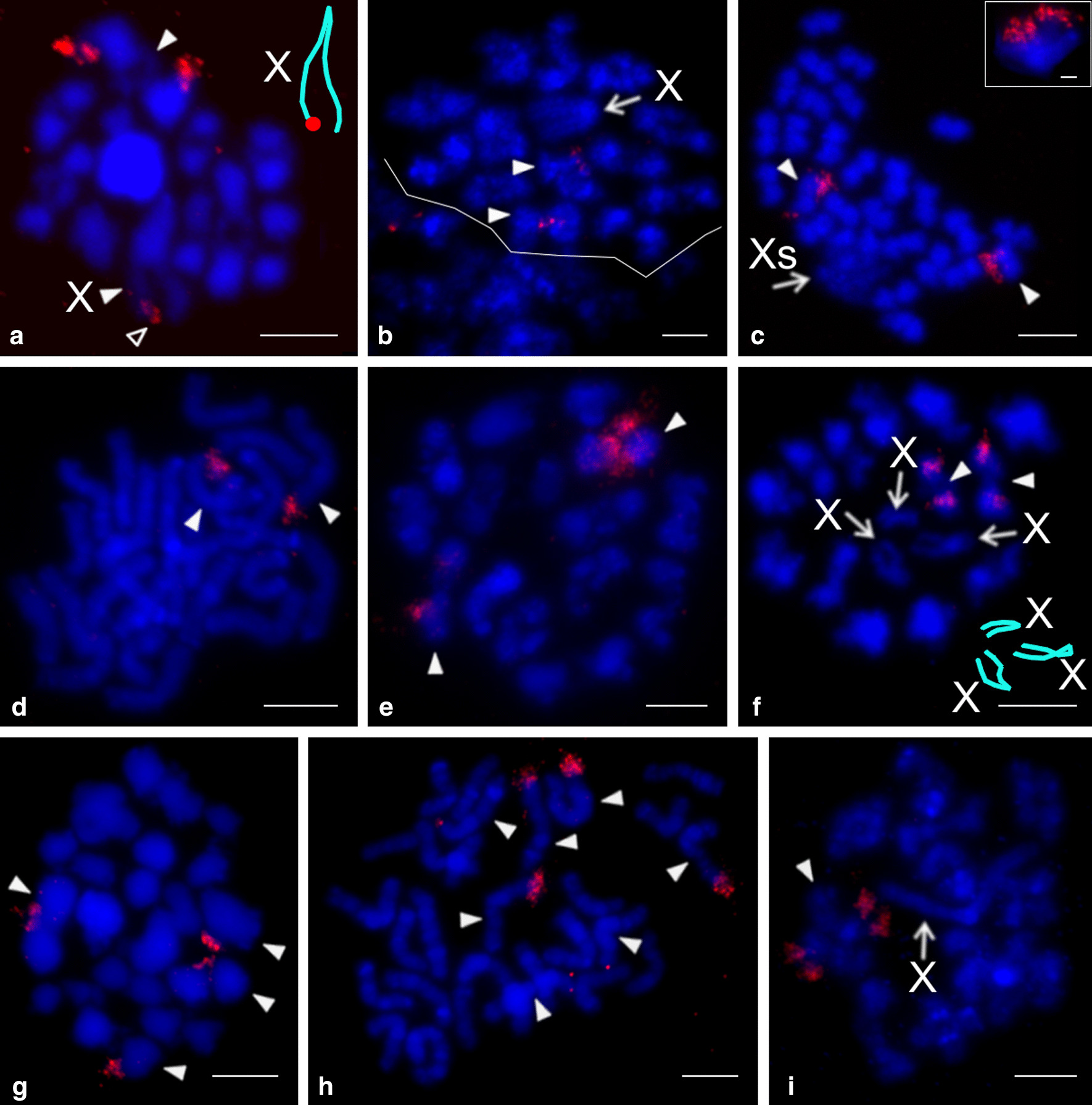
Smeringopinae, males, detection of NORs (FISH). *Arrowhead* = NOR-bearing chromosome (**a**, **c**, **d**, **h**) or bivalent (**a**, **b**, **e**–**g**, **i**), *open arrowhead* = sex chromosome-linked NOR, X = X chromosome, Xs = X chromosomes. **a** *Holocnemus hispanicus* (X0), metaphase I, the largest bivalent and one end of the X chromosome bear a terminal NOR (see scheme of X chromosome, red = NOR); **b** *H. pluchei* (X0), metaphase I (separated by a line from a diffuse stage), two bivalents containing a terminal chiasma have a NOR in chiasma region; **c** *Hoplopholcus forskali* (X_1_X_2_0)*,* plate formed by two fused sister prometaphases II. Sex chromosomes form a cluster having a very low condensation. Chromosomes of one pair bear NOR. Inset: NOR-bearing chromosome from another metaphase II. This chromosome exhibits metacentric morphology and terminal location of NOR; **d** *H. labyrinthi* (X_1_X_2_0), mitotic metaphase. Chromosomes of one pair bear terminal NOR; **e** *Smeringopus atomarius* (X_1_X_2_0), metaphase I, note two bivalents with NOR; **f** *S. pallidus* (X_1_X_2_X_3_0), two bivalents with NOR. Note scheme of sex chromosome association; **g**, **h** *Smeringopus* sp. (X_1_X_2_0), **g** metaphase I, four bivalents include NOR. **h** Mitotic metaphase, seven chromosomes show a terminal NOR; **i** *Stygopholcus skotophilus* (X0)*,* metaphase I, one large bivalent bears a terminal NOR. Bar = 5 µm, inset 1 µm

Smeringopines exhibited a considerable diversity of the NOR number (Additional file 19: Table S5). Their karyotypes contained one (*H. hispanicus*, *Hoplopholcus* species, *S. skotophilus*) (Fig. 12a, c, d, i), two (*Holocnemus pluchei*, *S. atomarius*, *S. pallidus*) (Figs. 8c, 12b, e, f) or four (*Smeringopus* sp.) (Fig. 12g, h) chromosome pairs bearing a NOR. In most species, the morphology of these pairs and the NOR location was also determined. Chromosome pairs bearing NORs were biarmed; NORs were terminal (Additional file 19: Table S5). *Smeringopus* sp. exhibited an odd number of chromosomes bearing a NOR, which indicates heterozygosity of one pair for the presence of a NOR (Fig. 12h). The karyotype of *H. hispanicus* (X0) also contained a sex chromosome-linked NOR, which was placed at the end of the X chromosome (Fig. 12a).

### Chromosome behaviour in the male germline

**Fig. 13:**
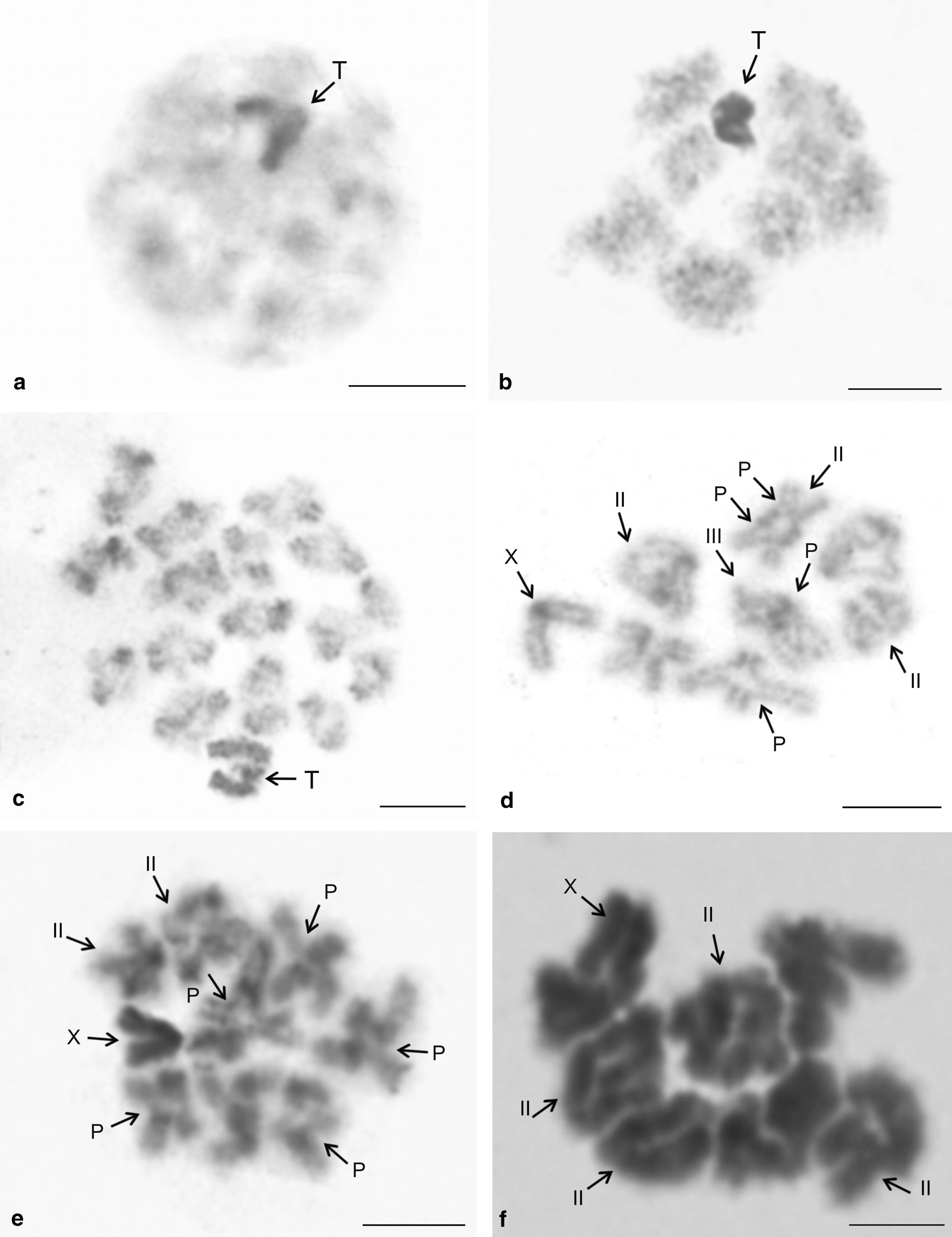
Meiosis of pholcid males, diffuse stage and pattern of chiasmata. P = pericentric chiasma, T = sex chromosome trivalent, X = X chromosome, II = bivalent with two chiasmata, III = bivalent with three chiasmata. **a** *Artema nephilit* (X_1_X_2_Y), early diffuse stage. Bivalents considerably decondensed, sex chromosomes form positively heteropycnotic body; **b** *Metagonia* sp. (X_1_X_2_Y), late diffuse stage, bivalents condensed but without well differentiated chiasmata. Sex chromosomes form a highly condensed body; **c** *Artema nephilit* (X_1_X_2_Y), diakinesis. Bivalents retain low condensation, except for terminal regions; **d** *Physocyclus dugesi* (X0), diplotene. Some bivalents have a pericentric chiasma. Centromere regions are dark, more condensed; **e** *Modisimus* cf. *elongatus* (X0), diplotene. Sex chromosome is positively heteropycnotic. Most bivalents have pericentric chiasma. Centromeric regions are dark, more condensed; **f** *Chisosa diluta* (X0), metaphase I. Most bivalents have two chiasmata. Bar = 10 µm

**Fig. 14:**
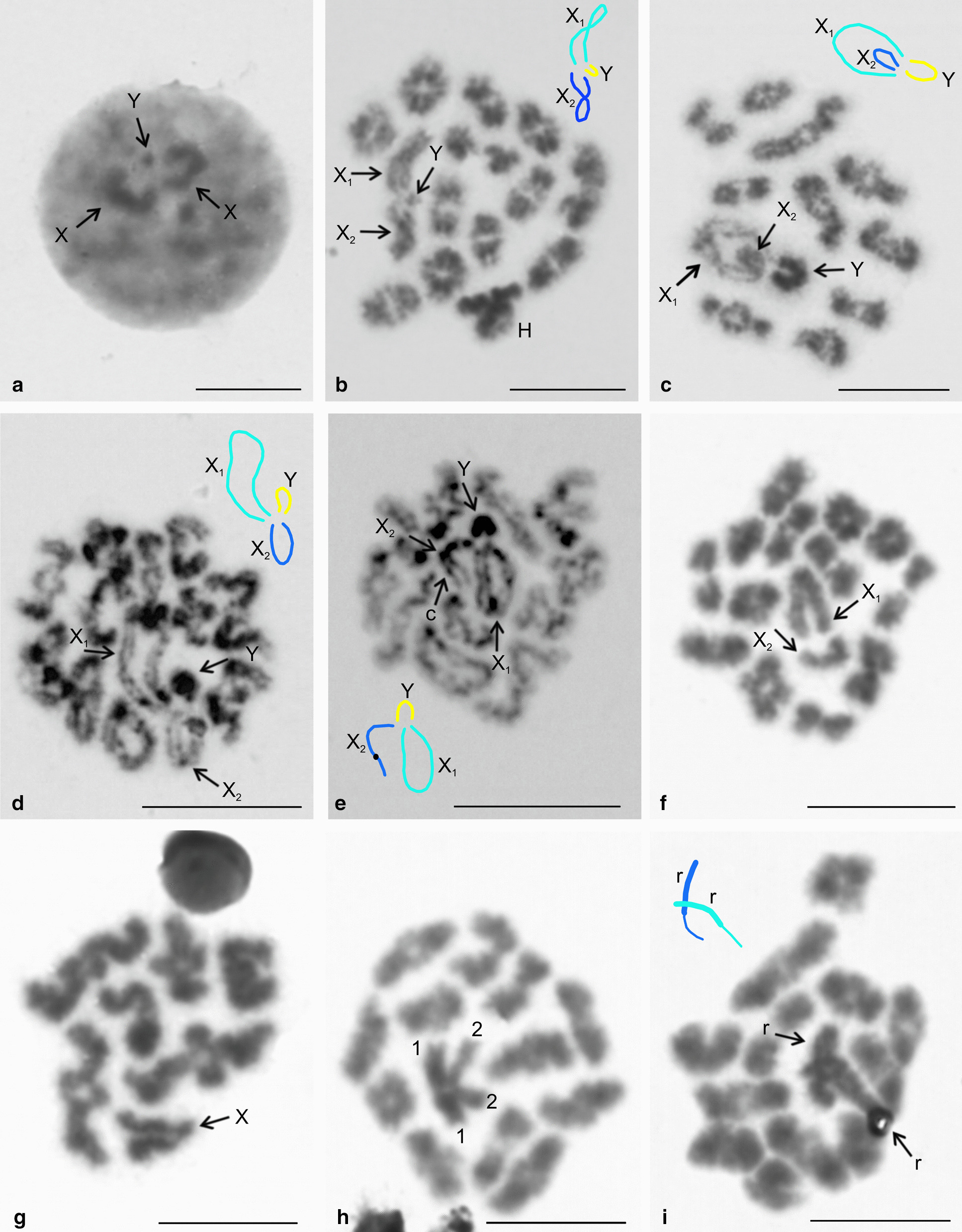
Meiosis of pholcid males, sex chromosome pairing. Figures **b**–**e** contain scheme of sex chromosome trivalent X_1_X_2_Y. H = positively heteropycnotic bivalent, X = X chromosome, X_1_ = X_1_ chromosome, X_2_ = X_2_ chromosome, Y = Y chromosome. **a**, **b** *Pholcophora americana* (X_1_X_2_Y). X chromosomes associated by ends of both arms with tiny Y chromosome. **a** Premeiotic interphase. **b** Diakinesis. Note positive heteropycnosis of a large bivalent; **c** *Pholcus kindia* (X_1_X_2_Y), diakinesis. Sex chromosomes pair by ends of both arms. Condensation of the X chromosomes is much lower than that of the Y chromosome; **d**, **e** *P. opilionoides* (X_1_X_2_Y), late prophase I, Y chromosome shows a considerable condensation. **d** Early diplotene. Both ends of X chromosomes involved in pairing. **e** Late diplotene. Both ends of X_1_ and Y chromosome take part in pairing. By contrast, only long arm of the X_2_ chromosome is involved in pairing (c—centromeric knob of X_2_ chromosome); **f** *Hoplopholcus forskali* (X_1_X_2_0), metaphase I, X chromosomes pair by ends of both arms in the middle of the plate; **g** *Cantikus sabah* (X0), diplotene. Note parallel association of the X chromosome arms; **h**, **i** *Kambiwa neotropica* (X_1_X_2_X_3_X_4_Y), metaphase I formed by 12 bivalents and a sex chromosome multivalent (in the middle of the plate). **h** positively heteropycnotic, cross-shaped sex chromosome multivalent is composed of two thick (1) and two thin "arms" (2). **i** Multivalent disintegrated into two rod-shaped structures (r). Each of them consists of one thick and one thin "arm" (see scheme). Bar = 10 µm

Males exhibited standard meiosis except for prophase I. Following pachytene, bivalents appeared globular and almost decondensed (so-called diffuse stage). In contrast, the sex chromosomes formed a highly spiralised and positively heteropycnotic body (i.e. stained more intensively than the other chromosomes) (Fig. 13a). In some pholcids, bivalents became considerably condensed during the late diffuse stage, whereas they retained a globular shape (e.g., *Smeringopus*). Despite condensation of bivalents, chiasmata were not discernible or only partially expressed (Fig. 13b). Following the diffuse stage, bivalents of some species uncoiled and exhibited a morphology typical for late prophase I (i.e., diplotene and diakinesis). Alternatively, the standard morphology of the bivalents emerged gradually during their condensation (Additional file 11: Fig. S8g). In some species, diplotene was reduced or even missing. In that case, bivalents showed a diakinetic morphology after the diffuse stage (Figs. 13c, 14b, c). In some pholcids, the bivalents (*Metagonia*, Additional file 11: Fig. S8e, f) or their middle part (*Artema nephilit*, Fig. 13c) retained a low condensation during late prophase I.

Male meiosis of most pholcids was remarkable for a very low chiasma frequency. The absolute majority of bivalents contained only a single chiasma (Additional file 2: Table S2, Additional file 7: Table S3, Additional file 12: Table S4, Additional file 19: Table S5, Fig. 14b–i, Additional file 11: Fig. S8d–f, h). An increased frequency of chiasmata (chiasma frequency ≥ 1.1 per bivalent) was found only in artemines with a low diploid number, in *Modisimus* cf. *elongatus* (Modisiminae), *Pholcus* sp., and *Quamtana filmeri* (Pholcinae) (Additional file 2: Table S2, Additional file 7: Table S3, Additional file 12: Table S4, Fig. 13d–f). In *Physocyclus dugesi* (Arteminae) and *M.* cf. *elongatus* (Modisiminae), we also found rare bivalents containing three chiasmata (Fig. 13d). In some species, centromeric regions formed a knob or flexure on the bivalents during late prophase I, which allowed us to determine the relative position of the chiasmata and centromere (Fig. 13d, e). Chiasmata of most analysed pholcids were predominantly distal and intercalar. In *Modisimus* and *Physocyclus*, pericentric chiasmata were frequent or even predominant (Fig. 13d, e). A large bivalent displayed positive heteropycnosis in late prophase I of *Pehrforsskalia* and *Pholcophora* (Fig. 14b, Additional file 11: Fig. S8g).

The sex chromosomes exhibited a specific behaviour in the male germline, which was often already initiated in the spermatogonia. During spermatogonial mitosis, the sex chromosomes differed often by condensation and pycnosis from the other chromosomes. In some species, they were more condensed and positively heteropycnotic. This behaviour was frequent in modisimines and pholcines (Additional file 24: Fig. S19a, b). In some *Pholcus* species, the Y chromosome exhibited a more intensive heteropycnosis than the X chromosomes. This Y chromosome pattern continued frequently at premeiotic interphase and meiosis (Fig. 14c–e, Additional file 11: Fig. S8i, Additional file 24: Fig. S19f, Additional file 25: Fig. S20a). In *Artema*, X chromosome condensation was delayed in comparison with the other chromosomes during spermatogonial metaphase (Additional file 24: Fig. S19c).

The sex chromosomes of some pholcids exhibited a specific location in the mitotic plate. In species with the X_1_X_2_Y system, they were often associated in the middle of the metaphase plate (*Artema*, *Nipisa*, *Leptopholcus*, *Pholcus*). While chromosomes X_1_ and X_2_ were often aligned in parallel (Additional file 24: Fig. S19b, c), chromosome Y was sometimes released from the association (Additional file 24: Fig. S19b). Sex chromosomes were usually not discernible at spermatogonial mitoses of pholcids with the X_1_X_2_0 system, due to the absence of heteropycnosis. In some species, however, it was possible to detect the prominent X_1_ chromosome. It was usually placed in the middle of the plate (Additional file 24: Fig. S19d). We did not find any preferential location of the sex chromosome in spermatogonia of pholcids with the X0 system.

Meiotic pairing of sex chromosomes was already established in the premeiotic interphase. It was initiated by a parallel attachment of the X chromosomes (Additional file 24: Fig. S19e). Following completion of the pairing, sex chromosomes formed a body on the periphery of the nucleus. They retained their peripheral position during early prophase I (leptotene–pachytene) and the diffuse stage. In species with the X_1_X_2_Y and X0 systems, this position was often retained during late prophase I (Figs. 13c–f, 14b, Additional file 11: Fig. S8d–h, Additional file 25: Fig. S20a). In contrast, in species with multiple X systems, as well as in *Kambiwa* (complex SCS), the sex chromosomes were preferentially located in the middle of the plate in late prophase and metaphase I (Fig. 14f, h, i).

Chromosomes of the X_1_X_2_Y, XY, and multiple X systems paired without chiasmata in male meiosis. Biarmed sex chromosomes paired by the ends of both arms (Fig. 14c, f, Additional file 11: Fig. S8d, Additional file 15: Fig. S11e, Additional file 22: Fig. S17a, Additional file 26: Fig. S21a); the arms of each chromosome were often associated (Figs. 6c, 12f, 13c, 14b, Additional file 11: Fig. S8d). This pattern was also exhibited by the tiny Y chromosome of *Aetana* and *Spermophora* (Additional file 11: Fig. S8d). It was impossible to determine the pairing mode of the Y chromosome in other pholcids with a tiny size of this element. In some smeringopines with the X_1_X_2_0 system and subtelocentric X_2_ chromosome, only the long arm of this element was involved in pairing in some plates during late prophase and metaphase I. Similarly, in some pholcines with a X_1_X_2_Y system and non-metacentric X_2_ (*Quamtana*, *Pholcus phalangioides*), only the long arm of the X_2_ took part in pairing during this period (Additional file 15: Fig. S11c, g). In these species, all plates exhibited the same pattern of sex chromosome pairing. In *P. opilionoides*, pairing of the short arm of the X_2_ chromosome was completed during early diplotene. Only the long arm of this chromosome was involved in pairing during late diplotene (Fig. 14d, e). In species with the X0 system, the arms of the X chromosome were usually associated with each other during late prophase and metaphase I (Figs. 5b, 12a, b, 13e, 14g, Additional file 5: Fig. S3c, e, Additional file 17: S13c, Additional file 23: Fig. 18a, c, e).

Our data suggest a complex sex chromosome pairing in *Kambiwa* (X_1_X_2_X_3_X_4_Y). During late prophase and metaphase I, sex chromosomes formed a cross-shaped multivalent consisting of four "arms" (Fig. 14h, Additional file 10: Fig. S7b). Two "arms" were thick and two were thin, which was most obvious in diakinesis (Additional file 10: Fig. S7a). The tiny Y chromosome was not discernible. It was only detected at spermatogonial mitosis and metaphase II (Additional file 11: Fig. S8a, c). In some plates, the multivalent disintegrated into two parts associated with each other. Each part was formed by one thick and one thin “arm" connected at one end (Fig. 14i).

Segregation of the sex chromosomes was usually delayed in anaphase I (Additional file 25: Fig. S20b). In some species, the sex chromosomes lagged even during telophase I. In species with two or three X chromosomes, the X chromosomes were arranged in parallel on the periphery of the plate during anaphase I. In contrast to the other chromosomes, chromatids of each X chromosome were associated during this period. Moreover, the arms of each X chromosome were also aligned (Additional file 25: Fig. S20b). The single X chromosome of the X0 system exhibited the same pattern of chromatid and arm association. The association of chromatids and arms of particular X chromosomes was completed during prophase or metaphase II (Additional file 25: Fig. S20d). Association of the X chromosomes continued until the end of meiosis (Additional file 15: Fig. S11d, Additional file 22: Fig. S17b). The sex chromosome of *Cantikus* (X0) was remarkable for a precocious centromere division at metaphase II (Additional file 25: Fig. S20f). In contrast, the X chromosomes of *Aetana* (X_1_X_2_Y) exhibited slightly delayed centromeric division during this period (Additional file 25: Fig. S20g). In anaphase II, the segregation of the X chromosome(s) was slightly delayed in some pholcids, namely in *Physocyclus* (Arteminae), *Psilochorus* (Modisiminae), *Holocnemus*, and *Hoplopholcus* (Smeringopinae) (Additional file 25: Fig. S20i). Remarkably, the Y chromosome was placed preferentially in the middle of the plate from anaphase I until the end of meiosis in both X_1_X_2_Y and XY systems (Additional file 25: Fig. S20h, Additional file 26: Fig. S21b).

The sex chromosomes of each species showed a specific pattern of condensation and pycnosis during male meiosis. They became condensed and positively heteropycnotic during the premeiotic interphase (Fig. 14a, Additional file 24: Fig. S19e, f), forming a sex chromosome body (SCB). Sex chromosome condensation decreased considerably during the onset of prophase I, which was frequently accompanied by the disintegration of the SCB and loss of positive heteropycnosis. The following period of increase of condensation and pycnosis of the sex chromosomes culminated during the pachytene and diffuse stage, which was often accompanied by restoration of the SCB (Fig. 13a, b). Condensation of the sex chromosomes was often considerably reduced during the transition from the diffuse stage to late prophase I, which was accompanied by disintegration of the SCB. In some taxa, recondensation of the X chromosomes was delayed during late prophase I in comparison to the bivalents and Y chromosome. This was the case in *Pholcophora* (Ninetinae), *Leptopholcus*, *Metagonia*, *Spermophora*, and *Pholcus* (except for *P. opilionoides* and *P. phalangioides*) (Pholcinae) (X_1_X_2_Y), where the X chromosomes continued low condensation until diakinesis (Figs. 9a, 14c, Additional file 11: Fig. S8e, f, Additional file 25: Fig. S20a). On the other hand, the sex chromosome(s) of some pholcids (e.g. *Holocneminus*, *Cantikus*, *Pehrforsskalia*) displayed no or only a slight transient decrease of condensation during the transition from diffuse stage to diplotene (Fig. 14g, Additional file 11: Fig. S8g, Additional file 25: Fig. S20c). The sex chromosomes of *Cantikus* formed a highly condensed SCB until early metaphase I.

The sex chromosomes were considerably condensed and positively heteropycnotic during interkinesis (i.e. interphase between the first and second meiotic division). In *Holocneminus*, this pattern persisted until prometaphase II (Additional file 25: Fig. S20e). In most other species, the sex chromosome condensation decreased considerably during the onset of prophase II. This event was often accompanied by the loss of positive heteropycnosis. The sex chromosomes of some species (e.g. *Hoplopholcus forskali*) were almost decondensed during prophase II. They recondensed suddenly during late prophase or prometaphase II (Additional file 25: Fig. S20d). The sex chromosomes of some pholcids also showed positive heteropycnosis during metaphase and anaphase II (e.g. Additional file 25: Fig. S20h, i).

Besides the germline cells, pholcid testes also frequently contained endopolyploid nuclei, whose size exceeded that of the diploid nuclei considerably. In some species, the sex chromosomes were positively heteropycnotic and formed a cluster in these nuclei (Additional file 27: Fig. S22).

## Discussion

### Diploid numbers and morphology of chromosome pairs

#### Evolution of chromosome numbers

**Fig. 15:**
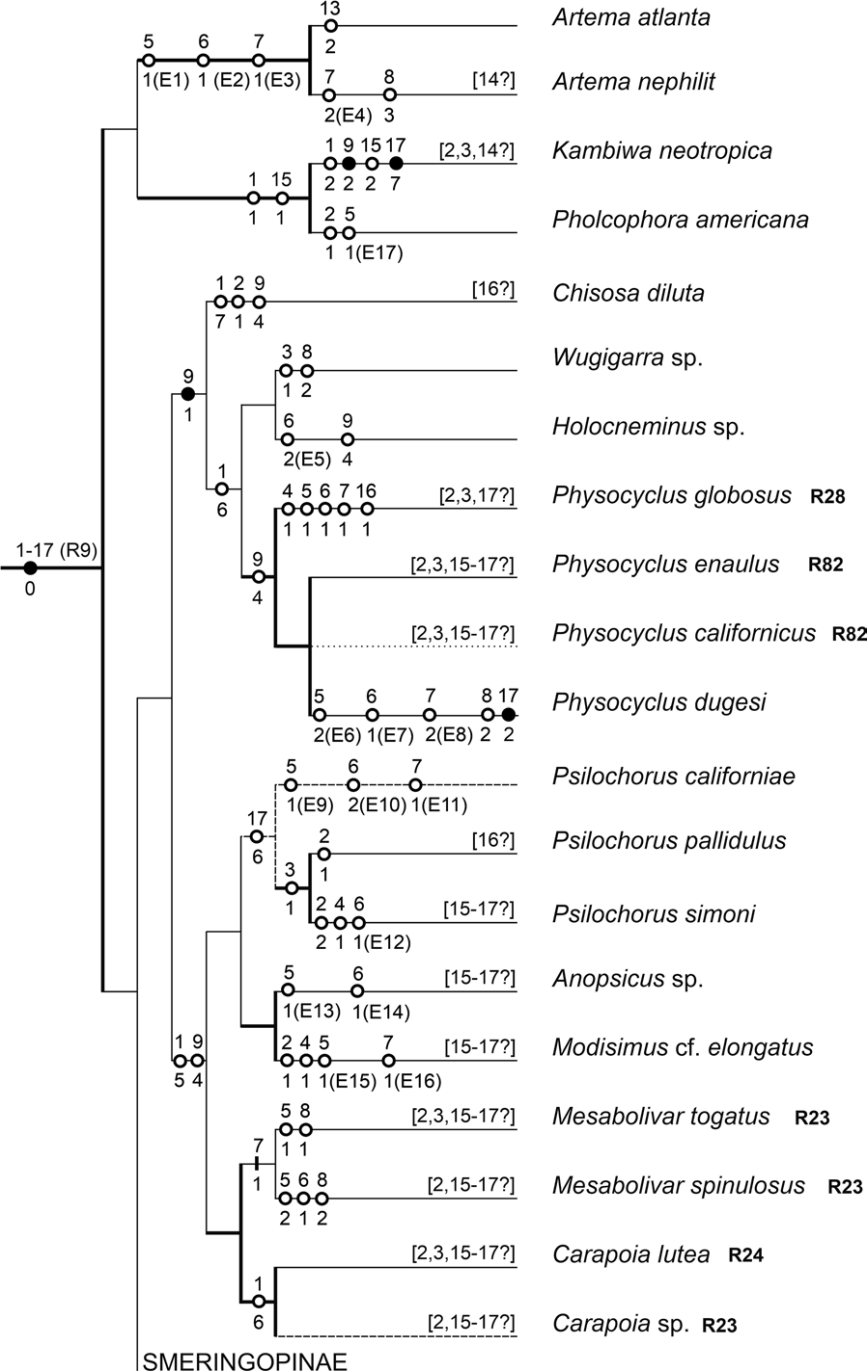
Arteminae, Modisiminae, and Ninetinae: karyotype characters mapped on a cladogram. Based on the most recent molecular phylogeny of Pholcidae [20, 21]. Standard continuous lines: low bootstrapping branch support, i.e. < 70%; thick continuous lines: modest to full support of hypotheses, ≥ 70% [21]; discontinuous lines: taxa not included in molecular phylogeny—phylogenetic position is based on cytogenetic data (dashed lines) or unresolved (dotted lines). Number above mark: number of character; number below mark: character state. Full marks: characters without homoplasy; empty marks: homoplasic characters. In some species, data on some characters are missing (information in square brackets). Selected events are marked E and numbered (see Additional file 1: Table S1 for chromosomes formed by these events). Chromosomes formed by the other events are identifiable unequivocally in Additional file 1: Table S1. Concerning chromosome pairs, only well-defined events changing chromosome morphology substantially are considered. Reconstruction of karyotype evolution is based on our data, except for taxa marked by the formula RX (see reference X for information). See “Methods” (pp. 32–33) for coding of characters

Male diploid numbers of pholcids range from 9 (three *Micropholcus* species) [25, this study] to 33 (*Artema* spp.) [this study]. The diploid number of the *Micropholcus* species is the lowest one found in araneomorph spiders with monocentric chromosomes so far [25]. Particular pholcid subfamilies differ by their range of numbers of chromosomal pairs (NCPs). Reported data suggest a low diversity of NCPs in modisimines (7–8 pairs) [25–27, this study], ninetines (12–13 pairs) [this study], and smeringopines (11–13 pairs) [9, 28–32, this study]. The other two subfamilies show a much higher range of NCPs: artemines from 6 to 15 pairs [30, 32, 33, this study] and pholcines from 4 to 11 pairs [25, this study]. A high range of NCPs in artemines could reflect the possible non-monophyly of this group (see Fig. 15). In pholcines, there are also reports on 12 chromosome pairs in two species, but these data are dubious. The number of chromosome pairs in the species analysed by Sharma and Parida [34] can not be verified from the published information. In the species studied by Wang et al. [35], the presented data do not allow to determine unequivocally the NCPs and SCS.

Remarkably, the karyotype of an early-diverging pholcid, *Artema* (2n♂ = 33, X_1_X_2_Y, chromosomes biarmed) is very similar to karyotypes of the haplogynes *Filistata insidiatrix* (Filistatidae; 2n♂ = 33, X_1_X_2_Y) [9], *Paculla* sp. (Pacullidae; 2n♂ = 33, X_1_X_2_Y) [14], and *Hypochilus pococki* (Hypochilidae; 2n♂ = 29, X_1_X_2_Y) [9]. Whereas *Artema*, *Filistata*, and *Paculla* exhibit the same diploid number and biarmed morphology of chromosome pairs, the karyotype of *Hypochilus* is slightly different, showing lower NCPs and a higher ratio of monoarmed chromosome pairs. The karyotype of *Hypochilus* can be derived from the pattern found in *Artema*, *Filistata*, and *Paculla* by two chromosome fusions and several pericentric inversions. This pattern of karyotypes suggests that the ancestral pholcid karyotype was close to those found in *Artema*, *Filistata* and *Paculla* (2n♂ = 33, X_1_X_2_Y, chromosomes biarmed).

Recent studies on spider phylogenomics suggest two primary lineages of haplogynes, one consisting of filistatids and hypochilids, and another one comprising synspermiate haplogynes, including pholcids and pacullids [10, 36, 37] (Fig. 1). Considering the placement of *Artema*, *Filistata*, *Paculla*, and *Hypochilus* in this phylogeny, the karyotype structure found in the three first genera (2n♂ = 33, X_1_X_2_Y, biarmed chromosomes) could be also the ancestral haplogyne karyotype. However, this conclusion conflicts with the hypothesis supposing that the ancestral karyotype of haplogynes was very close to that of entelegynes (20 chromosome pairs + X_1_X_2_0) [9]. This hypothesis is supported by the occurrence of very similar karyotypes in the haplogyne family Drymusidae (17 biarmed pairs + X_1_X_2_Y) and the family Austrochilidae (18 biarmed pairs + XY) [9], which is an early-diverging lineage of a clade formed by protoentelegynes and entelegynes [37]. The observed karyotype pattern of austrochilids and drymusids could reflect an early separation of drymusids within haplogynes, specifically before derivation of the clade including filistatids, hypochilids, pacullids, and pholcids. To determine the correct phylogenetic position of drymusids, more detailed phylogenomic analysis of early-diverging araneomorph clades is needed.

A fundamental trend of spider karyotype evolution is the reduction of chromosome numbers, which took place independently in many clades [38]. Our analysis suggests the same pattern in pholcids. Available data indicate the reduction to 13 chromosome pairs in the last common ancestor of smeringopines and pholcines, and separately in ancestral ninetines, or alternatively in the last common ancestor of ninetines, smeringopines and pholcines (see “Phylogenetic implications”, p. 29). Furthermore, a comparison of cytogenetic and molecular data suggests a further reduction to 11 pairs in ancestral pholcines, to 8 pairs in the common ancestor of modisimines, and to 7 pairs in the common ancestor of the artemine genera *Holocneminus*, *Physocyclus*, and *Wugigarra* (Fig. 15). The longest one or two chromosome pairs of pholcids are often prominent, which could reflect the formation of these pairs by fusion. In *Leptopholcus* (2n♂ = 17, X_1_X_2_Y), the first three pairs are considerably longer than the remaining ones. This set could be derived by three fusions from a karyotype containing ten chromosome pairs. Reductions of chromosome numbers also took place during the evolution of pholcid genera. However, the frequency of these events on genus level is low (Additional file 1: Table S1).

**Fig. 16:**
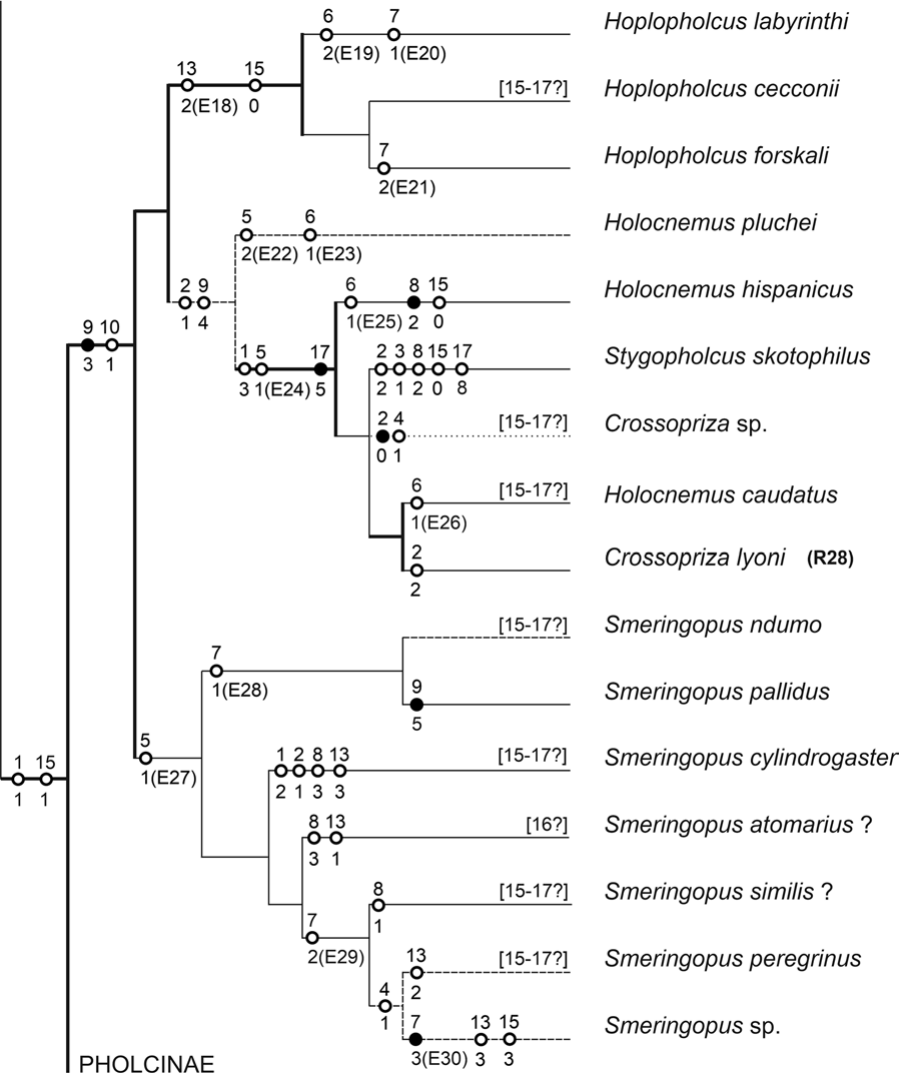
Smeringopinae: karyotype characters mapped on a cladogram. Based on the most recent molecular phylogeny of Pholcidae [20, 21]. Standard continuous lines: low bootstrapping branch support, i.e. < 70%; thick continuous lines: modest to full support of hypotheses, ≥ 70% [21]; discontinuous lines: taxa not included in molecular phylogeny—phylogenetic position is based on other sources [81] and/or on cytogenetic data (dashed lines) or unresolved (dotted lines). Number above mark: number of character; number below mark: character state. Full marks: characters without homoplasy; empty marks: homoplasic characters. In some species, data on some characters are missing (information in square brackets). Selected events are marked E and numbered (see Additional file 1: Table S1 for chromosomes formed by these events). Chromosomes formed by the other events are identifiable unequivocally in Additional file 1: Table S1. Concerning chromosome pairs, only well-defined events changing chromosome morphology substantially are considered. Reconstruction of karyotype evolution is based on our data, except for taxa marked by the formula (RX) (some information taken from reference X). See “Methods” (pp. 32–33) for coding of characters

Variation of NCPs has also been suggested on intraspecific level in pholcids, namely in *Crossopriza lyoni* [30]. This widespread synanthropic species has been karyotyped by several authors. Recent studies consistently report 11 biarmed chromosome pairs in this species; analysed populations came from Brazil, India, and Vietnam [30, 32–34, 39, this study]. This number of pairs is probably an ancestral feature of the clade formed by *Crossopriza*, *Holocnemus caudatus*, *H. hispanicus*, and *Stygopholcus* (Fig. 16). In contrast, initial cytogenetic studies on *C. lyoni* suggested a higher NCPs in Indian populations, namely 12 [29] or 13 [28]. While the first study does not contain information on chromosome morphology, the karyotype with 13 pairs was exclusively biarmed. This pattern could be derived from a set with 11 biarmed pairs by fissions of two pairs and subsequent inversions of four newly formed pairs. However, our results indicate a very low frequency of chromosome fissions during pholcid evolution and a low intraspecific variability of pholcid karyotypes. Therefore, differentiation of the karyotype of *C. lyoni* by multiple fissions is very unlikely. Diversity of NCPs in *C. lyoni* is most probably an artifact, possibly caused by species misidentifications. Reported data on NCPs also differ in *Micropholcus fauroti*. While Araujo et al. [26] reported eight metacentric pairs in a Brazilian population, we found only four metacentric pairs in another Brazilian population and African populations (Cape Verde, South Africa). Notably, we frequently found in our preparations of *Micropholcus* clusters formed by the fusion of chromosome plates. This artefact, along with the low number of observed plates, could have led Araujo et al. [26] to report double NCPs in this species. Four chromosome pairs were also reported in other karyotyped *Micropholcus* species [25].

#### Evolution of chromosome pairs

Similarly to the majority of other haplogynes with monocentric chromosomes [9], karyotypes of most pholcids are predominated by metacentric chromosomes. A different pattern has been reported only in two *Physocyclus* species [30, this study] and *Pholcus manueli* [35]. The karyotype of the latter species is suggested to be composed exclusively of monoarmed chromosomes. However, this idea is based only on the pattern of constitutive heterochromatin, i.e. a marker that could also be localised in regions other than the centromere. Therefore, this hypothesis should be tested by determination of the centromere position during mitotic metaphase or metaphase II. During metaphase II, chromatids of spider chromosomes are separated, except for the centromere.

At first glance, chromosome pairs of pholcids seem to be conservative elements, due to their predominantly metacentric morphology. However, comparison of closely related species revealed a dynamic nature of these pairs. Closely related taxa having the same number of pairs often differ by the morphology of one or several pairs, whose metacentric morphology is changed to submetacentric or even monoarmed (Additional file 1: Table S1). This pattern suggests the operation of pericentric inversions or some translocation variants. In some cases, the size of the pair is retained even after the change of its morphology to non-metacentric (e.g., acrocentric pair of *Artema nephilit*), which points to the operation of pericentric inversions. An enormous increase of the first pair (*Crossopriza lyoni*, *Holocnemus caudatus*) or considerable reduction of the last pair (some *Psilochorus* species, *Stygopholcus*, *Wugigarra* [this study], *Mesabolivar spinulosus* [25]), which are not accompanied by changes of NCPs, probably reflect an origin of these pairs by non-reciprocal or unequal reciprocal translocations. In the last three taxa, the last pair exhibits a subtelocentric morphology, which probably reflects translocation of most of an arm of an original biarmed pair to another chromosome. In *Aetana*, a considerable reduction of the last pair is accompanied by a change in morphology to acrocentric. This pattern could reflect centric fission of a biarmed chromosome pair, followed by integration of one product into another chromosome.

Diploid numbers of pholcids could be decreased by centric fusions of monoarmed pairs. Beside this, the number of pairs could be reduced by nested fusions. In contrast to centric fusions, this process involves biarmed chromosomes.

In conclusion, our data suggest the frequent involvement of fusions, inversions, and translocations of chromosome pairs in the karyotype evolution of pholcids. These kinds of rearrangements can be very effective in the formation of interspecific reproductive barriers [40, 41], and may thus have played a role in speciation processes in pholcids.

### Sex chromosomes

#### X_1_X_2_Y system

##### Ancestral composition and origin of the X_1_X_2_Y system

Pholcids exhibit a considerable diversity of sex chromosomes. We found six SCS in these spiders, namely X0, X_1_X_2_0, X_1_X_2_X_3_0, XY, X_1_X_2_Y, and X_1_X_2_X_3_X_4_Y. The X_1_X_2_Y system has been reported in seven haplogyne families, namely Sicariidae [9, 42, 43], Filistatidae [9, 14–16], Drymusidae, Hypochilidae, Pholcidae [9], Pacullidae [14], and Plectreuridae [15]. Specific morphology and the meiotic pairing of chromosomes X_1_, X_2_, and Y suggest that the X_1_X_2_Y system arose once in haplogynes [9]. Together with molecular and paleontological data, this points to an ancient origin of the haplogyne X_1_X_2_Y system. Plectreurids have been found in Jurassic strata [44]. A recent molecular phylogeny suggests the origin of spiders possessing an X_1_X_2_Y system during the early Mesozoic [37]. The phylogenetic distribution of the X_1_X_2_Y system using recent phylogenomic trees [10, 36, 37] suggests that this sex chromosome determination is ancestral for haplogynes, including pholcids. It could have arisen even earlier, namely before the separation of entelegyne and haplogyne spiders [16] or before the separation of mygalomorphs and araneomorphs in ancient opisthothele spiders. If so, the supposed ancestral sex chromosome system X_1_X_2_0 of mygalomorphs [6] and entelegynes [9] arose from the X_1_X_2_Y system by the loss of the Y chromosome. The ancestral X_1_X_2_Y system probably consisted of two large metacentric X chromosomes of similar size and a metacentric Y microchromosome [9]. Species with this pattern have been found in almost all X_1_X_2_Y families [9, 16, 18, this study]. The X_1_X_2_Y system was supposed to arise by rearrangements between autosomes and sex chromosomes [43]. In this case, it would be an ancient neo-sex chromosome system. According to our hypothesis, chromosomes X_1_, X_2_, and Y are derived from CSCP. It was suggested that multiple X chromosomes of spiders arose by nondisjunctions of the X chromosome of the CSCP [7]. The Y chromosome could originate in a similar way, namely by a nondisjunction of the Y chromosome of the CSCP and subsequent degeneration of the newly formed element. Nondisjunctions could be a major mechanism of formation of multiple X chromosomes in spiders [7, 18, 45]. This unusual origin of sex chromosomes is supported by the inactivation of multiple X chromosomes during meiosis of spider females. This unique behaviour probably evolved to avoid the negative effects of duplicated X chromosomes on female meiosis [5, 7].

According to our data, chromosomes of the X_1_X_2_Y system are dynamic elements. Although they exhibit a conservative pairing during male meiosis, they underwent frequent rearrangements during pholcid evolution. Remarkably, chromosomes X_1_, X_2_, and Y differ by their pattern of morphological evolution and evolutionary plasticity. See Additional file 28: Appendix S1 for evolution of particular sex chromosomes of the X_1_X_2_Y system.

##### Conversion of the X_1_X_2_Y system into other sex chromosome systems

In some haplogynes, including pholcids, the X_1_X_2_Y system has been transformed into other SCS [9, 15, this study]. According to [9], the X_1_X_2_Y system underwent conversion to the X0 system, namely by X chromosome fusion (formation of the XY system) and subsequent loss of the Y chromosome. Our results suggest that patterns of conversion of the X_1_X_2_Y to the X0 system are more diversified. In some pholcids, loss of the Y chromosome preceded fusion of the X chromosomes, which resulted in the formation of the X_1_X_2_0 and, subsequently, of the X0 system. We suppose that this scenario could be more frequent during the evolution of the haplogyne X_1_X_2_Y system, due to specific features of its Y chromosome, which could facilitate degeneration and extinction of this element (tiny size, absence of recombinations between the X and Y chromosome).

#### XY system

In *Wugigarra* the X_1_X_2_Y system was transformed into the XY system by X chromosome fusion [this study]. The XY system of another haplogyne, *Diguetia* (Diguetidae), is suggested to arise by the same process [9]. The sex chromosomes of *Wugigarra* and *Diguetia* retained their metacentric morphology, except for the acrocentric X chromosome of *D. canities*, which probably originated from metacentric X by pericentric inversion [9]. The Y chromosomes of these haplogynes are small elements. While sex chromosomes of *Wugigarra* retain their achiasmatic pairing by both ends, only one X chromosome end is involved in achiasmatic pairing in *Diguetia* [9]. Notably, the XY system has also been suggested in other pholcids, namely *Smeringopus ndumo* (reported as *S. pallidus*) [9] and *Holocnemus pluchei* [31]. Our data showed that these reports are erroneous, based on revision of original preparations (*S. ndumo*) and laboratory stock studied by previous authors (*H. pluchei*). An XY system was also discovered in several other spiders [6, 9, 46, 47]. Its origin is, however, different. It is suggested to have arisen from the X0 determination by a fusion of the X chromosome and an autosome(s), resulting in the formation of neo-X and neo-Y chromosomes. Another possible origin of the system might be by a fusion of the X chromosome and X chromosome of the CSCP.

#### Multiple X chromosome systems

##### X_1_X_2_0 system

Although the X_1_X_2_0 system is the most frequent sex chromosome determination in entelegyne araneomorphs [18, 45], it is rare in haplogyne araneomorphs [9, 15, this study]. Provided that the X_1_X_2_Y system is ancestral for araneomorphs [16], the X_1_X_2_0 system has originated in the same way in both entelegynes and haplogynes, namely from the X_1_X_2_Y system by the loss of the Y chromosome. The X_1_X_2_Y system of the pholcids *Aetana* and *Artema*, including a minute Y chromosome [this study], could represent an evolutionary transition between the X_1_X_2_Y and X_1_X_2_0 systems. In haplogynes, the X_1_X_2_0 system has originated several times, namely in filistatids [15], plectreurids [9], and pholcids [this study].

In pholcids, we found the X_1_X_2_0 system in two smeringopine genera, *Hoplopholcus* and *Smeringopus*. Mapping of the distribution of the X_1_X_2_0 system in smeringopines suggests an origin of this system in their ancestor. Remarkably, we never found the X_1_X_2_0 system in pholcids in which it was reported previously (see database of Araujo et al. [24]). Instead, these species exhibit the X_1_X_2_Y system, which had probably been mistaken for the X_1_X_2_0 system, due to the small size of the Y chromosome. The X_1_X_2_Y system was revealed in pholcids less than two decades ago [9].

Remarkably, the X_1_ and X_2_ chromosomes of the X_1_X_2_0 system show a similar evolution to the X chromosomes of the X_1_X_2_Y system in pholcids (see Additional file 28: Appendix S1 for evolution of chromosomes of the X_1_X_2_Y system). While the X_1_ is a conservative element retaining large size and metacentric morphology, X_2_ underwent reduction and frequent changes of morphology. The size of the X_1_ chromosome is more variable (7.9–15.1% of TCL) than that of the X_2_ element (5.1–7.4% of TCL), which could reflect insertions of fragments derived from CPs into the X_1_ chromosome. In each species, the X_2_ chromosome is reduced in comparison with the X_1_ chromosome (including representatives with an ancestral state, i.e. metacentric morphology of the X_2_ chromosome). Therefore, the X_2_ chromosome was probably already reduced in an ancestor of smeringopines (having a X_1_X_2_0 or X_1_X_2_Y system). The original metacentric X_2_ chromosome has been transformed several times to a monoarmed one, namely in the ancestor of *Hoplopholcus* and in several *Smeringopus* species. The morphology of the X_2_ chromosome has most probably changed by a pericentric inversion or translocation.

##### X_1_X_2_X_3_0 system

A sex chromosome system comprising three X chromosomes is relatively frequent in entelegyne [48] and mygalomorph spiders [6]. Among haplogynes, *Smeringopus pallidus* is the first reported species with this sex chromosome determination [this study]. It was obviously derived from the X_1_X_2_0 system found in the other *Smeringopus* species. However, the origin of the third X chromosome of *S. pallidus* is unresolved. The behaviour of the three X chromosomes of this species during male meiosis (pairing, pattern of condensation and heteropycnosis) is the same as in X_1_X_2_0 species, which contradicts the possible origin of the extra X chromosome from autosomes. Another possibility is nondisjunction of an X chromosome, which is supposed to be a mechanism of X_1_X_2_X_3_0 formation in entelegynes [7, 45, 49]. This hypothesis is supported by the same meiotic behaviour of sex chromosomes in X_1_X_2_0 and X_1_X_2_X_3_0 species. The sex chromosomes of *S. pallidus* (two large metacentric X chromosomes of similar size + one small metacentric X chromosome) can be derived from the ancestral pattern of the X_1_X_2_0 system in *Smeringopus* (two metacentric X chromosomes, X_2_ considerably smaller than X_1_) by nondisjunction of the X_1_ chromosome. Formation of an additional large X chromosome would substantially increase the sum of the relative lengths of the X chromosomes. However, the values do not differ substantially between *S. pallidus* (16.9% of TCL) and the karyotypically most similar *S. ndumo* (18% of TCL), and fall within the range of values found in other *Smeringopus* species (14.2–19.8% of TCL). Therefore, the X_3_ chromosome of *S. pallidus* more likely arose by a fission of the ancestral metacentric X_1_ chromosome into two acrocentric chromosomes, followed by their pericentric inversion. This hypothesis is supported (1) by a reduction of the X_1_ chromosome of *S. pallidus* in comparison with other *Smeringopus* species, and (2) by the sum of X_1_ and X_3_ sizes in *S. pallidus*, which is similar to the X_1_ size in the karyotypically most similar *S. ndumo*. To resolve the origin of the X_1_X_2_X_3_0 system in *S. pallidus*, sequencing of its X chromosomes needs to be carried out.

#### X0 system

##### Phylogenetic distribution and origins of the pholcid X0 system

The X0 system is common in pholcids. It has been reported in some artemines, pholcines, smeringopines, and all modisimines karyotyped so far [24, this study]. Character mapping suggests at least five origins of the X0 system in pholcids, namely in the (1) artemines with low diploid numbers (see "Phylogenetic implications", p. 29), (2) ancestor of modisimines (Additional file 29: Fig. S23), (3) common ancestor of smeringopines *Holocnemus*, *Crossopriza*, and *Stygopholcus* (Additional file 30: Fig. S24), (4) pholcine *Belisana*, and (5) common ancestor of the pholcines *Cantikus* and *Micropholcus* (Additional file 31: Fig. S25). The X0 system was also reported in *Pholcus manueli* [35]. However, chromosome plates obtained in *P. manueli* (male mitoses) do not allow us to determine the SCS of this species unequivocally. Similar to *P. phalangioides* (X_1_X_2_Y) [9], the karyotype of *P. manueli* is composed of 25 chromosomes and contains an odd peculiar chromosome formed exclusively by constitutive heterochromatin [35]. The odd heterochromatic element of *P. phalangioides* is a Y chromosome [9]. Therefore, *P. manueli* exhibits with all probability the X_1_X_2_Y system.

The X0 system is common in spiders. Phylogenetic distribution of the X0 system in spiders suggests multiple origins of this system [6, 18, 45]. X0 systems of entelegynes and mygalomorphs arose by chromosome fusions from multiple X chromosome systems [6, 18, 45]. In haplogynes, the X0 system has been found in nine families (see database [24]). The X0 system of haplogynes with monocentric chromosomes arose from XY or X_1_X_2_0 systems. The X0 system of smeringopine pholcids arose from the X_1_X_2_0 system by X chromosome fusion [this study]. In contrast, the X0 system of a clade formed by *Chisosa*, *Holocneminus*, *Physocyclus*, and *Wugigarra* probably arose from the XY system (Additional file 29: Fig. S23). Formation of the X0 system in the other pholcids is unresolved. Our discovery of both X0 [this study] and ancestral X_1_X_2_Y systems [J. Král and O. Košulič, unpublished] in the same genus, the pholcine *Belisana*, indicate a relatively fast formation of the X0 system during the evolution of this clade. An increase in the size of the Y chromosome during the evolution of pholcines (see Additional file 28: Appendix S1 for evolution of the Y chromosome) did not prevent this element from being subjected to reduction and loss in the common ancestor of *Cantikus* and *Micropholcus* (X0)*.*

##### Evolution of the X0 system in pholcids

Despite its multiple origins, the single X chromosome of pholcids exhibits an extremely conservative morphology. Except for *Modisimus* it is always mediocentric (Additional file 1: Table S1), which indicates a strong selection pressure to keep this feature. We suppose that this morphology could be essential to ensure the self-association and regular segregation of the X chromosome univalent during male meiosis. In mice, the meiotic stability of the sex chromosome univalents is promoted by their self-association [50].

In contrast to morphology, the size of the X chromosome varies considerably, namely from 8.89 (*Belisana sabah*) to 21.21% of TCL (*Chisosa diluta*) (Additional file 1: Table S1). A considerable diversity of X chromosome sizes occurs even on a genus level (*Crossopriza*, *Holocnemus*, *Psilochorus*). The increase in size of the X chromosome could be a consequence of an integration of material from CPs, as suggested, for example, by the large difference of X chromosome size between two *Holocnemus* species (*H. caudatus* and *H. hispanicus*).

#### X_1_X_2_X_3_X_4_Y system

Comparison of data on the male karyotype and meiosis suggests that the X_1_X_2_X_3_X_4_Y system occurs in *Kambiwa*. This pholcid exhibits one chromosome pair less than the other studied ninetine, *Pholcophora*, which displays a X_1_X_2_Y system [this study]. This pattern indicates the origin of the X_1_X_2_X_3_X_4_Y system by rearrangements between chromosomes of the X_1_X_2_Y system and a chromosome pair. The Y microchromosome was not involved in the rearrangements. Sex chromosome systems arising by rearrangements between chromosome pair(s) and sex chromosomes have also been reported in some other araneomorphs [7, 9, 47, 51–53] and in some mygalomorphs [6, 46]. These events are always apomorphies of low-level taxa (species or species groups), which indicates their relatively recent origin.

#### Sex chromosomes and pholcid speciation

In summary, our data suggest frequent and diverse structural changes of sex chromosomes during pholcid evolution. These events include inversions and translocations of sex chromosomes, integration of fragments or even whole chromosomes into the SCS, and loss of the Y chromosome. Since structural changes of sex chromosomes are often very potent in formation of interspecific reproduction barriers [54–57], they have probably been involved in the speciation process in pholcids. In keeping with this view, closely related pholcids often differ in sex chromosome morphology [this study].

Particular sex chromosomes of pholcid males do not recombine, which suggests a high degree of differentiation of these chromosomes. This degree of sex chromosome differentiation is associated with markedly stronger reproductive isolation [58]. Although sex chromosome rearrangements could be less detrimental in meiosis of spider males due to achiasmatic sex chromosome pairing, in female heterozygotes they can lead to a collapse of homologous sex chromosome pairing.

### Nucleolus organizer regions

#### NOR bearing pairs

The pattern of NORs has only been determined in a low number of spiders so far. Usually, these structures were detected by silver staining, a technique that visualizes NORs that were active in the preceding interphase only [59]. Therefore, it is impossible to reconstruct the evolution of NORs in particular families or even higher taxa of spiders. Karyotypes of most spiders contain, however, a low number of these structures. The common ancestral pattern of opisthothele spiders comprised probably one or two chromosome pairs bearing a terminal NOR locus [6].

In our study, we analysed the evolution of spider NORs on a family level for the first time. Nucleolus organizer regions have been visualized only in three pholcids so far, in each case by silver staining [30, 32]. To reconstruct NOR evolution in pholcids, we analysed 30 pholcid species, including one species studied earlier by silver staining. To detect NORs, we used fluorescence in situ hybridization (FISH), which visualizes inactive NORs as well. According to our results, pholcids exhibit a considerable diversity of NOR numbers (from one to nine loci); congeneric species often differ in the number of NOR loci. Almost all detected NOR loci are homozygous for the presence of NOR, which suggests a high stability in the number of NOR loci at species level. Findings of loci heterozygous for the presence of NOR are rare in pholcids (*Nipisa*, *Pholcus pagbilao, Smeringopus* sp*.*) [this study]. In the case of *Artema atlanta*, intraspecific variability of NORs is doubtful. While the studied karyotype from South Africa contained one NOR locus [this study], two NOR loci were reported in an Indian population probed by silver staining [32]. Considering the low quality of the signals detected, the information of the latter authors should be verified by FISH.

Character mapping suggests that ancestral pholcids had a single biarmed CP bearing a terminal NOR (Additional file 29: Fig. S23). Whereas the number of NORs underwent multiple changes during pholcid evolution, the pattern of their location has remained conservative. Each NOR-bearing chromosome pair of pholcids includes a single NOR, except for exceptional pairs having NORs at both ends. These pairs were only found in some pholcines [this study]. NORs of pholcids are terminal, except for a pericentric NOR of *Physocyclus globosus* [30], which might originate from a paracentric inversion. The terminal location of pholcid NORs suggests that these structures have mostly spread by ectopic recombination. This mechanism could be promoted by hybridization of individuals belonging to populations differing in the number of NOR loci, which is indicated by the finding of heterozygotes for the presence of NOR in *Nipisa, P. pagbilao*, and*, Smeringopus* sp.

Our data indicate an increase in the number of NOR bearing CPs in some pholcid clades. In the ancestor of ninetines and the common ancestor of smeringopines and pholcines (Additional file 29: Fig. S23, Additional file 30: Fig. S24) or common ancestor of ninetines, smeringopines, and pholcines (see "Phylogenetic implications", p. 29), the number of NOR-bearing pairs increased to two. During subsequent evolution, NORs spread to three chromosome pairs in *Kambiwa* (Ninetinae) and in the ancestor of pholcines. Karyotypes of *Smeringopus* sp. and some pholcines contain four NOR-bearing pairs. In *Belisana*, there are even five pairs bearing NORs. On the other hand, character mapping suggests that the number of NOR-bearing pairs decreased several times in smeringopines (*Stygopholcus*, *Holocnemus hispanicus*, *Hoplopholcus*) and pholcines (*Aetana*, *Muruta*, *Quamtana*, common ancestor of *Cantikus* and *Micropholcus*) (Additional file 30: Fig. S24, Additional file 31: Fig. S25). Remarkably, these clades often exhibit reduced NCPs in comparison with their relatives; NORs retain a terminal position.

##### Sex chromosome-linked NORs

In contrast to other opisthothele spiders, haplogynes often exhibit sex chromosome-linked NORs [9]. These NORs are also common in pholcids. They have been found in all pholcid subfamilies [30, this study]. Sex chromosome-linked NORs of pholcids and other haplogynes are always restricted to the X chromosome(s) [9, 30, this study] except for *Nipisa* [this study]. Character mapping suggests at least five origins of these NORs in pholcids, namely in (1) *Physocyclus* (Arteminae, X0), (2) *Kambiwa* (Ninetinae, X_1_X_2_X_3_X_4_Y), (3) *Psilochorus* (Modisiminae, X0) (Additional file 29: Fig. S23), (4) the common ancestor of *Crossopriza lyoni* and *Holocnemus hispanicus* (Smeringopinae, X0) (Additional file 30: Fig. S24), and (5) during early evolution of pholcines (X_1_X_2_Y) (Additional file 31: Fig. S25). In modisimines, NOR data are available for *Psilochorus* only. Therefore, their sex chromosome-linked NORs could have arisen earlier than during the evolution of this genus. Sex-chromosome linked NORs of pholcids always have a terminal location, which suggests an origin of these structures by ectopic recombination.

Most pholcids with the X0 system and sex-chromosome-linked NORs display two terminal NORs (e.g., *Physocyclus*, *Psilochorus*) [this study]. The second NOR was probably formed by ectopic recombination between the arms of the X chromosome, which was facilitated by their meiotic association. Although the *Physocyclus* species analysed in our paper exhibit two sex chromosome-linked NORs, a study on *P. globosus* did not reveal these structures [30]. Since these authors used silver staining and gonial mitoses to detect NORs, it is possible that sex-chromosome linked NORs of this species are inactivated in the germline cells. Alternatively, *P. globosus* may belong to a *Physocyclus* lineage that lacks sex chromosome-linked NORs.

We revealed the most complex evolution of sex chromosome-linked NORs in the X_1_X_2_Y system of pholcines. According to our data, sex chromosome-linked NORs already appeared during the early evolution of pholcines, no later than before the separation of the clade containing *Aetana*. Paleontological [60] and molecular data [61] indicate relative antiquity of the pholcine lineage with sex chromosome-linked NORs (further SCL-NOR clade). According to the latter dataset, this clade evolved at the latest during the Jurassic. The ancestral pattern of sex chromosome-linked NORs was probably formed by a single terminal locus on the X_1_ chromosome, which is retained in *Aetana*. In another early-diverging pholcine, *Belisana*, the X_1_X_2_Y system has been transformed to X0. During this transformation, the terminally positioned sex chromomosome-linked NOR originating from the X_1_ chromosome was probably retained. The second NOR of *Belisana* was probably formed by ectopic recombination between the arms of the single X chromosome.

The subsequent evolution of NORs in the pholcine X_1_X_2_Y system probably involved gradual spreading of the NORs by ectopic recombination to the other X_1_ end, and after that to one or both ends of the X_2_ chromosome. Remarkably, the X chromosome ends are attached in male meiosis to ensure sex chromosome pairing, which could promote spreading of NORs among sex chromosomes by ectopic recombination. We suppose that spreading of NORs by ectopic recombination could also be facilitated by the association of X chromosome bivalents during female meiosis of spiders [5]. Besides pholcines, multiple SCS containing NORs have been found only in the ninetine *Kambiwa*. In contrast to pholcines, the end of the X chromosome containing NOR is not involved in chromosome pairing in this spider (see “Sex chromosome behaviour”, p. 26).

As demonstrated in some animals, sex chromosome-linked NORs can ensure achiasmatic pairing of sex chromosomes [62, 63]. The specific terminal location of sex chromosome-linked NORs in pholcines suggests involvement of these structures in achiasmatic pairing as well. Pairing ensured by NORs could be more stable, which may have promoted an increase in Y chromosome size during the evolution of pholcines. We suppose that the terminal NOR(s) of pholcids with the X0 system have a similar function as in the X_1_X_2_Y system, i.e. they could strengthen the association of the X chromosome arms during male meiosis.

Remarkably, our data suggest the loss or degeneration of sex chromosome-linked NORs in some pholcids, especially in the SCL-NOR clade. The most obvious cases are *Pholcus pagbilao* and the clade formed by *Cantikus* and *Micropholcus.* In contrast to *Pholcus phalangioides*, we did not detect the target 18S rDNA motif on the sex chromosomes of *P. pagbilao*. This sequence could be degenerated or lost during the evolution of NORs involved in pairing. A similar pattern was found in some *Drosophila* species, in which most rDNA of sex chromosome-linked NOR is lost, except for motifs involved in pairing [64]. Loss/degeneration of sex chromosome-linked NORs in the common ancestor of the pholcines *Cantikus* and *Micropholcus* was accompanied by a considerable decrease of NCPs and formation of the X0 system in this lineage.

Some authors propose that sex chromosome-linked NORs could be a synapomorphy of some haplogyne clades [9, 30]. Our data support another scenario, namely multiple invasions of NORs on sex chromosomes of haplogynes by ectopic recombinations. Although ectopic recombination is probably also a frequent mechanism of NOR dispersion in entelegynes [65], these spiders exhibit a much lower frequency of sex chromosome-linked NORs than haplogynes [5]. It was suggested that the dispersion of NORs on spider sex chromosomes by ectopic recombination is reduced by sex chromosome inactivation in meiosis of both spider sexes [65]. Since sex chromosomes of haplogynes show a similar pattern of meiotic condensation and heterochromatinisation as entelegynes [9, this study], it would be interesting to determine the mechanisms facilitating the transmission of NORs on haplogyne sex chromosomes. It could be the lower degree of X chromosome condensation during some periods of male prophase I [this study]. In keeping with this hypothesis, NORs have almost never been detected on the Y chromosome of the haplogyne X_1_X_2_Y system [9, this study], which could be a consequence of its considerable condensation in the germline. Another possibility of frequent spreading of NORs on sex chromosomes of haplogynes could be the presence of NOR on their CSCP. Chromosomes of the CSCP are associated with the other sex chromosomes during male meiosis [7], which could facilitate spreading of NORs on these sex chromosomes.

### Chromosome behaviour in the male germline

#### Modifications of meiotic division

The prophase of the first meiotic division is modified in pholcid males. Following pachytene, nuclei enter the so-called diffuse stage. The male diffuse stage was also found in other haplogynes, in the protoentelegyne family Leptonetidae [9], and in some clades of early-diverging mygalomorphs [6] and entelegynes [66]. The diffuse stage evolved in other animal groups as well. In some pholcids, the diplotene is reduced: recondensed bivalents exhibit a diakinetic morphology after the diffuse stage [this study]. This pattern has also been found in some other haplogynes [9, 67].

Pholcid karyotypes are predominated by biarmed chromosomes, which generally form more chiasmata than monoarmed ones [68]. In spite of this, most pholcids show a very low chiasma frequency in male meiosis (Additional file 2: Table S2, Additional file 7: Table S3, Additional file 12: Table S4, Additional file 19: Table S5). This pattern is probably an ancestral pholcid feature. The frequency of chiasmata is increased particularly in some pholcids with a low diploid number, namely some artemines, modisimines, and pholcines [25, 26, this study]. Since a reduction of diploid number is accompanied by decrease of total chiasma number per karyotype, increase of chiasma frequency per bivalent could be a compensatory mechanism to avoid reduction of genetic variability in these species. Pholcids usually exhibit a predomination of intercalar and distal chiasmata. A different pattern has been found in *Modisimus* and *Physocyclus*, which show a relatively high proportion of pericentric chiasmata. Relocation of chiasmata in these spiders could be a consequence of inversions, which can change the position of chiasmata [69].

#### Polyploid cells

Germline cells of spider males form spermiocysts, whose cells are connected by cytoplasmic bridges and synchronized during their development [70]. As a result, plates belonging to the same mitotic or meiotic phase are frequently fused on chromosome preparations [26, 30]. Besides these artefacts resembling polyploid plates, preparations of pholcid testes contained many large endopolyploid nuclei, which could belong to a specific cell lineage. Occurrence of these nuclei in testes reflects a high metabolic activity of testicular tissues. Overcondensation of sex chromosomes in endopolyploid nuclei of some pholcids could reflect transcriptional repression of these chromosomes. Endopolyploid nuclei were also reported in the testes of some other opisthothele spiders [5, 6, 71]. In pholcids, they have only been reported in the silk and venom glands so far [72].

#### Sex chromosome behaviour

Spider sex chromosomes are distinguished by a specific behaviour in the male germline [5, 6, 18, 38, 45, 73], which is sometimes already initiated at spermatogonial mitosis [5]. In some pholcids, the sex chromosomes exhibit a specific condensation during this division. Chromosomes of the X_1_X_2_Y system are usually associated in spermatogonia, whereas X chromosomes are often arranged in parallel [this study]. Moreover, sex chromosomes of this system are preferentially located in the middle of spermatogonial metaphases [9, this study]. Concerning the other pholcid sex chromosome systems, we were not able to determine the relative position of their chromosomes in spermatogonia. Notably, multiple X chromosomes of some entelegynes also show specific condensation, and similar arrangement and location at spermatogonia, as found in the X_1_X_2_Y system [5].

Despite the considerable diversity of SCS in pholcids, pairing of their sex chromosomes during male meiosis is conservative. Similar to other spiders [5], it is already established during premeiotic interphase [9, this study]. Pairing of chromosomes of the X_1_X_2_Y system is initiated by the parallel attachment of X chromosomes [this study]. The mode of chromosome pairing within the pholcid X_1_X_2_Y system is the same as in other haplogynes [9]. Biarmed chromosomes of this system pair without chiasmata, namely by the ends of both arms [9, this study]. This sex chromosome attachment is probably an ancestral mode of achiasmatic sex chromosome pairing in spiders [5]. It is retained in achiasmatic XY and multiple X chromosome systems of pholcids [this study]. Remarkably, pairing of the monoarmed X_2_ chromosome is modified in some pholcids. In *Artema* (X_1_X_2_Y) and smeringopines (X_1_X_2_0), the monoarmed X_2_ shows standard pairing. However, attachment of the short arm is less stable in the latter group during late prophase and metaphase I. In some pholcines with monoarmed X_2_, only the long arm of the X_2_ takes part in pairing during this period [9, this study]. In contrast, there are pholcines with monoarmed X_2_, both ends of which participate in pairing. This pattern indicates that restriction of pairing to the long arm of X_2_ does not depend only on the morphology of this chromosome. Concerning the X0 system, arms of the sex chromosome are associated together in pholcids during late prophase and metaphase I [this study]. This pattern is also common in other spiders exhibiting the X0 system and biarmed sex chromosome [5, 9].

**Fig. 17:**
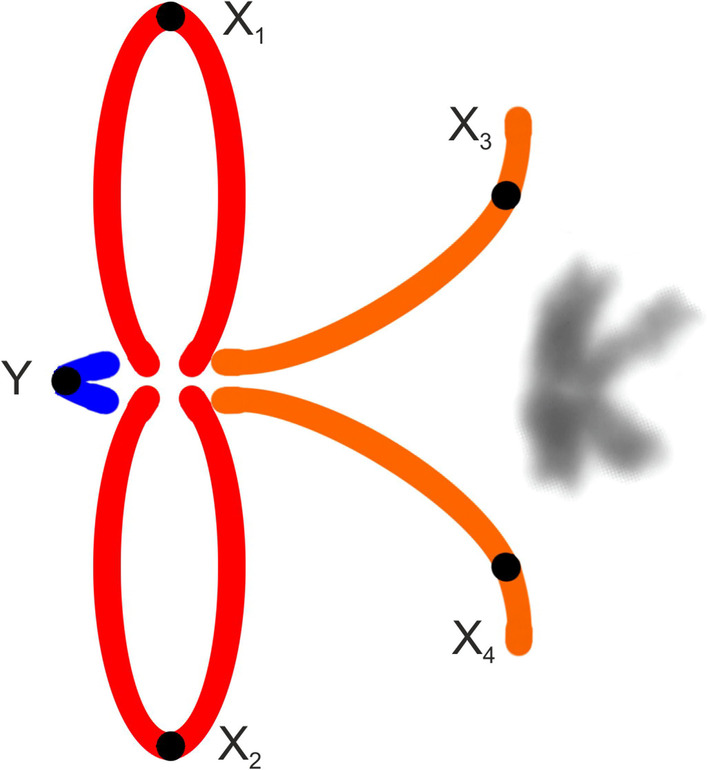
*Kambiwa neotropica* (X_1_X_2_X_3_X_4_Y), male meiosis, hypothesis on sex chromosome pairing. Pairing between X_1_ and X_3_ as well as X_2_ and X_4_ is more stable than the other attachments. Location of the Y microchromosome is unresolved. It is probably placed in the middle of the sex chromosome multivalent. This assumption is based on the pattern of meiotic pairing between the X chromosomes and Y microchromosome(s) in achiasmatic sex chromosome systems of haplogynes with more than two X chromosomes. *Red* = biarmed X chromosomes, *black knob* = centromere, *blue* = Y chromosome, *orange* = monoarmed X chromosomes. On the right—photograph of the sex chromosome multivalent

Our data suggest a possible pattern of sex chromosome pairing in the X_1_X_2_X_3_X_4_Y system of *Kambiwa*. As indicated above, two of its X chromosomes are probably biarmed and two other X are monoarmed. X chromosomes of *Kambiwa* probably pair by the ends of their arms during male meiosis. According to our model (Fig. 17), both arms of the biarmed X chromosomes are involved in pairing. One monoarmed X chromosome bears a NOR at the end, which is not involved in pairing. Since this NOR is placed in the short arm, only the long arm of this chromosome is involved in pairing. We hypothesize the same mode of pairing for the second monoarmed X chromosome. Our data indicate that the sex chromosome multivalent of *Kambiwa* contains two X chromosome pairs; each of them consists of one biarmed and one monoarmed chromosome. Pairing between these pairs is less stable than within them. We assume a similar location of the Y chromosome as in the achiasmatic sex chromosome systems of other haplogynes with more than two X [14], namely in the middle of the multivalent.

Meiotic segregation of the sex chromosomes is modified in pholcid males. Regardless of the type of SCS, it is usually delayed in anaphase I. The sex chromosome(s) of some pholcids show precocious or delayed division during metaphase II. In some pholcids, X chromosome segregation is also delayed during the second meiotic division [this study].

As in other spiders, the sex chromosomes of pholcids are usually placed at the periphery of the plate during premeiotic interphase, prophase and metaphase I. Notably, our data suggest relocation of sex chromosomes to the middle of the plate during late prophase I in pholcids with multiple X systems. The same behaviour of X chromosomes has evolved in males of avicularioid mygalomorphs [6]. Its function is unclear. After segregation during anaphase I, X chromosomes of pholcids retain their association until the end of meiosis, whereas the Y chromosome tends to be placed in the middle of the plate [this study].

As in other spiders, the course of sex chromosome condensation and pycnosis is complicated and species-specific in male meiosis of pholcids. The Y chromosome is often more condensed than the X chromosomes. In some pholcids, condensation of the X chromosomes is delayed during diplotene and diakinesis (e.g. *Nipisa*, *Leptopholcus*, most *Pholcus* species) [this study].

It should be underlined that chromosomes of the CSCP can also possess a specific behaviour in the male germline of spiders. In some mygalomorphs, they are associated and exhibit precocious chromatid separation in spermatogonial mitosis. Furthermore, they are heterochromatic in some male meiotic phases of some haplogynes and mygalomorphs [5, 6, 9]. Therefore, a large heterochromatic pair observed in male prophase I of several pholcids is probably a CSCP [this study]*.*

### Phylogenetic implications

**Fig. 18:**
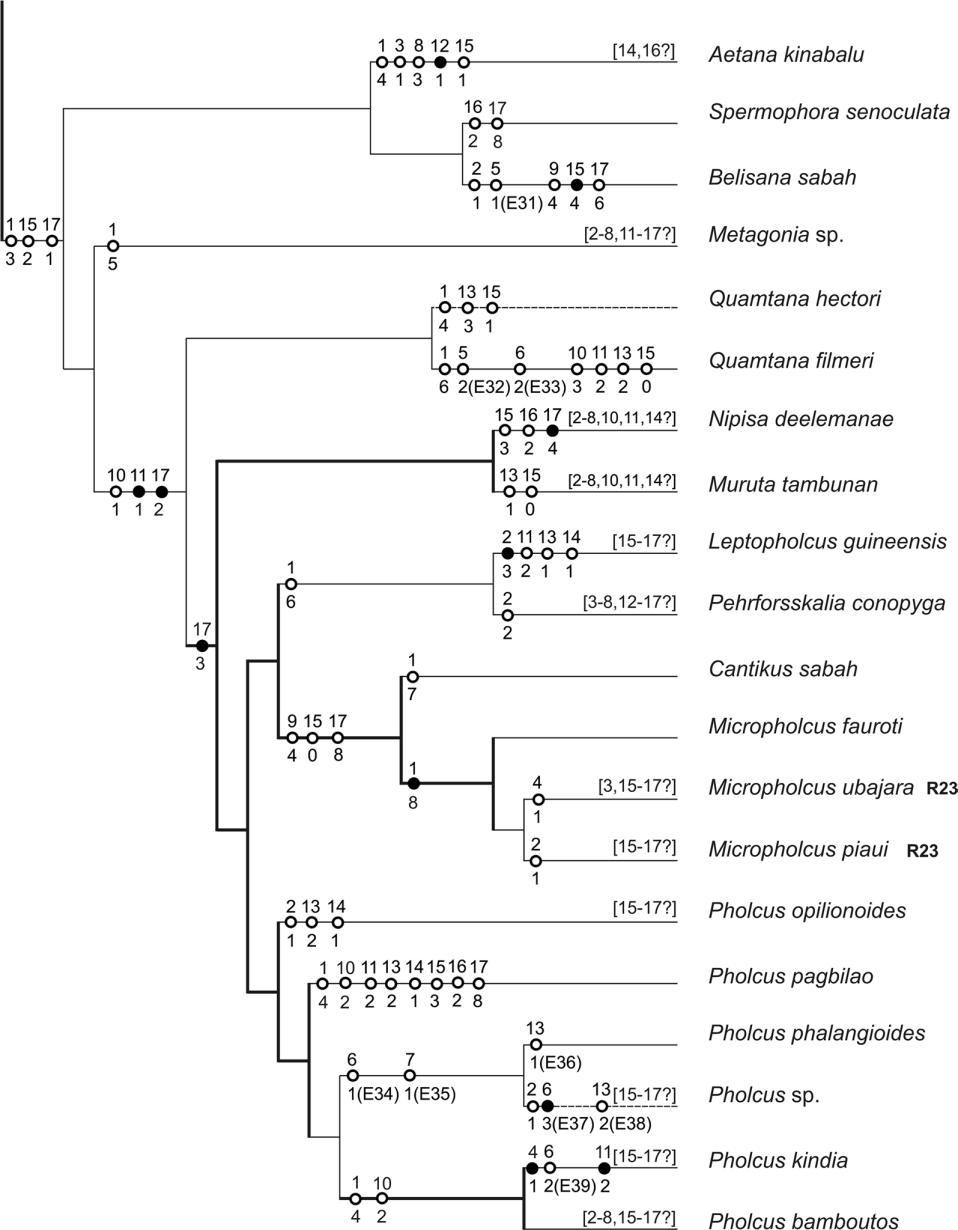
Pholcinae: karyotype characters mapped on a cladogram. Based on the most recent molecular phylogeny of Pholcidae [20, 21]. Standard continuous lines: low bootstrapping branch support, i.e. < 70%; thick continuous lines: modest to full support of hypotheses, ≥ 70% [21]; discontinuous lines: taxa not included in molecular phylogeny—phylogenetic position is based on cytogenetic data (dashed lines) or unresolved (dotted lines). Number above mark: number of character; number below mark: character state. Full marks: characters without homoplasy; empty marks: homoplasic characters. In some species, data on some characters are missing (information in square brackets). Selected events are marked E and numbered (see Additional file 1: Table S1 for chromosomes formed by these events). Chromosomes formed by the other events are identifiable unequivocally in Additional file 1: Table S1. Concerning chromosome pairs, only well-defined events changing chromosome morphology substantially are considered. Reconstruction of karyotype evolution is based on our data, except for taxa marked by the formula RX (see reference X for information). See "Methods" (pp. 32–33) for coding of characters

Despite the relatively limited sample of studied species, our study emphasizes the potential of karyotype data as an independent source of information for phylogenetic reconstruction. Based on character mapping, many chromosome features were identified as apomorphies, which can be potentially used to reconstruct pholcid phylogeny. Most of these features concern the number of chromosome pairs, chromosome morphology, SCS, and NOR pattern. Character mapping also suggests, however, a high level of homoplasy and many characters that need to be mapped on terminal branches, especially those concerning chromosome morphology. In general, this suggests a limited use of certain karyotype data for the reconstruction of pholcid phylogeny. However, numerous clades established on the basis of morphological and/or molecular data are in fact supported by karyotype data:

- At the level of subfamilies: e.g. Smeringopinae (2 characters) and Pholcinae (3 characters), sister relationship of Smeringopinae and Pholcinae (2 characters) (Figs. 16, 18).
- At the level of genus-groups, e.g.:
  - Sister-group relationship between *Quamtana* and the *Pholcus* group of genera (three characters) (first proposed based on morphology [76], later supported by molecular data [20]) (Fig. 18).
  - Clade formed by all artemine genera except *Artema* (in our sample *Chisosa*, *Wugigarra*, *Holocneminus*, and *Physocyclus*) (1 character) (Fig. 15).
  - Separation of *Stygopholcus* and *Hoplopholcus* (Fig. 16) (only character 15 supports a close relationship between the two genera, as originally claimed e.g. by Brignoli [75–77]).
  - Sister group relationship between *Cantikus* (previously in *Pholcus*) and *Micropholcus* (three characters) (strongly supported by molecular data, [20]) (Fig. 18).
- At the level of genera: e.g., *Artema* (three characters), *Hoplopholcus* (two characters) (Figs. 15, 16).

In some cases, the karyotype data suggest plausible alternative topologies that should be tested by molecular (ideally phylogenomic) approaches.

- Position of Ninetinae. Previous hypotheses placed ninetines either as sister to all other pholcids [61, 78] or together with *Artema* as sister to all other pholcids [20]. Ancestral ninetines probably exhibited 13 chromosome pairs, as found in *Pholcophora*. The ancestor of another studied ninetine, *Kambiwa* (12 pairs), probably had the same NCPs as *Pholcophora*; one pair has most probably been incorporated into the SCS. Mapping of chromosome data on the molecular tree suggests two chromosome changes in the ancestors of Ninetinae (decrease of NCPs from 15 to 13 and increase of NOR number to two loci) and the same two changes in the common ancestor of Smeringopinae + Pholcinae (Figs. 15, 16). Our chromosome data thus suggest a sister-group relationship between Ninetinae and Smeringopinae + Pholcinae. Clearly, the position of ninetines continues to be unclear.
- Phylogeny of artemines with low diploid numbers. The molecular phylogeny suggests the following topology: *Chisosa* (*Physocyclus* (*Wugigarra* + *Holocneminus*)) [21]. In this clade, the X0 system could either arise once, with subsequent reversion to the XY system (in *Wugigarra*), or it possibly evolved three times from the XY system, namely in *Chisosa*, *Holocneminus*, and *Physocyclus* (Fig. 15, Additional file 29: Fig. S23). The first hypothesis is improbable, since the X and Y chromosomes of *Wugigarra* exhibit the same mode of meiotic pairing as the chromosomes of the ancestral pholcid X_1_X_2_Y system. This mode of pairing would probably not be retained, if a reversion to the XY system had occurred. Although the second hypothesis (multiple convergent origin of the X0 system from the XY system) is supported by several independent origins of the X0 system in other pholcids, karyotype data lend some support to an alternative topology (*Wugigarra* (*Chisosa* (*Holocneminus* + *Physocyclus*))), which includes only a single origin of the X0 system from the XY system in the clade, namely at the base of the clade formed by the genera *Chisosa*, *Holocneminus*, and *Physocyclus*. This scenario contradicts a fairly strong node linking *Wugigarra* and *Holocneminus* (bootstrapping support 81) [21], but the alternative topology supported by karyotype data should be further tested too.
- Position of *Spermophora*. This spider differs from most other pholcines by the absence of sex chromosome-linked NORs. According to the molecular phylogeny, *Spermophora* belongs to an early-diverging clade of pholcines, which also includes the genera *Aetana* and *Belisana* [21], both of which have sex chromosome-linked NORs (Fig. 18, Additional file 31: Fig. S25). In this phylogeny, the absence of sex chromosome-linked NORs in *Spermophora* reflects NOR loss. An alternative hypothesis, based on chromosomes, is that the absence of sex chromosome-linked NORs in *Spermophora* represents a symplesiomorphy of pholcids rather than a loss, suggesting a sister-group relationship between *Spermophora* and all other pholcines. This is a plausible scenario because the position of *Spermophora* (and also that of *Aetana* and *Belisana*) was not strongly resolved in the molecular phylogeny.
- Phylogeny of the CHS clade (formed by *Crossopriza*, *Holocnemus*, and *Stygopholcus*). The molecular phylogeny suggests the topology *H. hispanicus* (*Stygopholcus* (*H. caudatus* + *Crossopriza*))) (Fig. 16). Karyotype data show a specific medium-sized submetacentric pair (feature 6-1) in *H. caudatus and H. hispanicus*, which evolved either once or twice. This uncertainty is related to the question of *Holocnemus* monophyly: *H. caudatus* and *H. hispanicus* might be sister taxa, sharing feature 6-1. Molecular data did not support such a relationship, but also did not strongly contradict it [20]. The two species are geographic neighbors and are morphologically similar, which lends further credibility to the karyotype data. The type species of *Holocnemus*, *H. pluchei*, is a morphologically isolated species and probably not closely related to *H. caudatus* and *H. hispanicus* (B.A. Huber, unpublished data). Chromosome data suggest it is probably sister taxon to the other taxa of the CHS clade (Fig. 16). Mapping of the chromosome information on a molecular cladogram suggests that eleven CPs is a synapomorphy of *Stygopholcus*, *Holocnemus caudatus*, *H. hispanicus*, and *Crossopriza* (Fig. 16). The molecular cladogram also suggests another synapomorphy for this clade, namely a sex chromosome-linked NOR. This structure is already present at the base of this clade in *H. hispanicus*, i.e. before the separation of *Stygopholcus* (Fig. 16)*.* In contrast to *H. hispanicus*, however, *Stygopholcus* does not exhibit sex chrososome-linked NOR, indicating secondary loss of this marker. The chromosome data suggest a more plausible explanation of the pattern found in *Stygopholcus*. Similar to *Spermophora*, the absence of sex chromosome-linked NOR may represent a symplesiomorphy of pholcids rather than a loss, which suggests a sister-group relationship between *Stygopholcus* and a clade formed by *H. caudatus*, *H. hispanicus*, and *Crossopriza*.
- *Smeringopus* phylogeny. Molecular data suggested the topology *S. pallidus* (*S. cylindrogaster* (*S. atomarius* (*S. similis* + *S. peregrinus*))) (Fig. 16) (accepting that *S. peregrinus*, which was not included in the analysis, and *S. peregrinoides* are closely related). Mapping of chromosome data on this cladogram suggests two independent origins of the specific shortest chromosome pair exhibiting an acrocentric morphology, namely in *S. cylindrogaster* and *S. atomarius*. This pair might arise by pericentric inversion from a short subtelocentric pair, which is a synapomorphy of *S. similis* and *S. peregrinus*. Chromosome data indicate that the shortest chromosome pair exhibiting an acrocentric morphology may be a synapomorphy of *S. cylindrogaster* and *S. atomarius*. This is in agreement with a morphological cladistic analysis [79], suggesting that the sister-group relationship between *S. cylindrogaster* and *S. atomarius* indicated by the karyotype data is a plausible alternative to the molecular hypothesis.
- Position of *Pholcus phalangioides*. Mapping of chromosome information on the cladogram derived from molecular data suggests an independent decrease of NCPs from eleven to ten and a considerable decrease of X_2_ size in the two *Pholcus* clades, namely *P. pagbilao* and African *Pholcus* species (Fig. 18). Chromosomes suggest an alternative topology, namely a single origin of these features at the base of a clade formed by African and South Asian members of *Pholcus*. A closer relationship of African *Pholcus* with the Southeast Asian *P. pagbilao* than with *P. phalangioides* is plausible, as the latter was identified as a rougue taxon in [20], and is thus likely misplaced in Fig. 18.

## Conclusions

Our study focused on the chromosome evolution of pholcids. A high species diversity of these spiders makes them a suitable model to analyse the fundamental trends of karyotype differentiation in haplogyne araneomorphs, a large spider clade comprising 20 families and more than 6000 species. Although the karyotype evolution of haplogynes includes many specific traits, it is not satisfactorily understood.

As in most other haplogynes, pholcids exhibit low to modest diploid numbers (2n♂ = 9–33). The diploid number of *Micropholcus* (2n♂ = 9) is the lowest found in araneomorph spiders with monocentric chromosomes so far. Pholcid karyotypes are predominated by metacentric chromosomes, which is another typical haplogyne feature. The evolution of pholcid chromosome pairs included frequent centric fusions, pericentric inversions, and translocations. Pholcid SCS are diversified. The ancestral sex chromosome determination of haplogynes, including pholcids, is presumably the X_1_X_2_Y system, which exhibits a specific chromosome morphology and achiasmatic pairing in male meiosis. The chromosomes of this system are already associated during spermatogonial mitoses. Chromosomes of the X_1_X_2_Y system differ in the pattern of their evolution. In some pholcid lineages, the X_1_X_2_Y system is converted into multiple X or XY systems, which retain the original achiasmatic sex chromosome pairing, and subsequently into the X0 system. Our data also suggest frequent integration of autosome fragments into sex chromosomes, as well as inversions and translocations of these chromosomes. Evolution of some pholcine X_1_X_2_Y lineages has included an enormous increase in size of the Y chromosome. Concerning haplogynes, there are no SCS formed by rearrangements between chromosome pair(s) and achiasmatic sex chromosomes, except for *Kambiwa*, which indicates potent constraints preventing these events. Our study also provides a first analysis of NOR evolution within a spider family. Pholcids display a considerable diversity of NOR patterns. The ancestral pattern was probably formed by a single terminal NOR locus. The subsequent evolution was accompanied by multiple increases of NOR number, as well as multiple invasions of NORs on sex chromosomes. Almost all NORs display a terminal location, which probably reflects their preferential spreading by ectopic recombination. The X_1_X_2_Y system of pholcines shows a specific distribution of sex chromosome-linked NORs, which are located almost exclusively at X chromosome ends involved in pairing. This pattern indicates involvement of NORs in achiasmatic pairing. In some pholcines these NORs were lost or degenerated. As in other haplogynes, the prophase of the first meiotic division is modified in pholcid males. It includes a diffuse stage, which is distinguished by an extreme decondensation of bivalents. Another specific feature of male meiosis is a very low recombination frequency in most pholcids.

Our study suggests a very low intraspecific diversity of pholcid karyotypes. Closely related species differ, however, often by the morphology of several chromosome pairs and sex chromosomes, as well as NOR pattern. Based on this pattern, we suppose that these changes can already appear during intraspecific karyotype differentiation. In several species, an intraspecific diversity of NOR pattern is suggested by the occurrence of heterozygotes for NOR. Rearrangements of chromosome pairs and sex chromosomes probably contribute towards the formation of interspecific reproductive barriers. Rapid sex chromosome evolution of pholcids may accelerate species diversification of these spiders. Although some karyotype features of pholcids are highly homoplasic, others are congruent with previous phylogenetic hypotheses based on morphology and/or molecules, suggesting a considerable potential for phylogenetic reconstruction. In some cases, our karyotype data suggest plausible alternative hypotheses on pholcid phylogenetic relationships, which can be tested by approaches of molecular phylogeny and phylogenomics. Our study offers novel hypotheses on karyotype evolution of haplogyne spiders, which can be tested by methods of molecular biology and cytogenetics in pholcids, as well as other haplogyne clades. Pholcids seem to be a suitable model group to analyse (1) participation of NORs in achiasmatic pairing, (2) co-evolution of sex chromosomes and NORs, (3) evolutionary differentiation of SCS composed of non-recombining chromosomes, and (4) increase and rejuvenation of the Y chromosome during evolution.

## Methods

### Material

In total, 197 specimens belonging to 47 pholcid species were analysed. Information on the studied species (number of analysed specimens, their sex, ontogenetic stage, and locality data) and deposition of voucher specimens are given in Additional file 32: Table S6.

### Preparation of Giemsa-stained slides and their evaluation

Chromosome plates were obtained from subadult or adult males, specifically from testes or the whole content of the abdomen (in small species). Slides were prepared by the spreading technique [80]. Tissues were hypotonized in 0.075 M KCl for 20–25 min at room temperature (RT) and fixed twice (10 and 20 min) in ethanol: acetic acid (3:1) (RT). The cell suspension was prepared from a piece of fixed tissue in a drop of 60% acetic acid on a slide using a pair of tungsten needles. The preparation was placed on a histological plate (40 °C). The drop was moved by a tungsten needle until almost complete evaporated. The remaining suspension was discarded. Slides were stained using 5% Giemsa solution in Sörensen buffer (pH 6.8) for 28 min (RT).

Preparations were inspected under an Olympus BX 50 microscope equipped with DP 71 CCD camera. To construct the karyotype, in most cases several metaphases II or mitotic metaphases (preferably five), were analysed to determine the relative chromosome length (RCL) and chromosome morphology. In the case of metaphases II, plates containing both sister cells were evaluated, except for *Aetana*, *Artema nephilit*, *Hoplopholcus labyrinthi*, and *Quamtana filmeri*. In these species, only single metaphase II cells were available. In some species, determination of chromosome morphology was difficult due to chromosome coiling. In most cases, it was impossible to distinguish the CSCP from autosomes. Therefore, the CSCP and autosomes are referred to collectively as chromosome pairs. Relative chromosome length was estimated as a percentage of the total chromosome length of the haploid set (TCL). Chromosome morphology was based on the position of the centromere [81], which was calculated as the ratio of the longer and shorter chromosome arms. Based on this ratio, four chromosome morphologies were recognized, specifically metacentric (1.0–1.7), submetacentric (1.71–3.0), subtelocentric (3.01–7.0), and acrocentric (> 7). Some chromosomes exhibited a transitional morphology between two of these types. In these cases, chromosome morphology was denoted by the formula x/y (this formula means transition between morphology x and y). Mediocentric chromosomes (1.0–1.3) were considered as a subset of metacentric chromosomes. Metacentric and submetacentric chromosomes were considered as biarmed, and subtelocentric and acrocentric chromosomes as monoarmed. The sex chromosome system was usually identified from meiosis of the heterogametic sex, based on segregation of the sex chromosomes and/or their behavior in prophase and metaphase I. In *Psilochorus californiae*, the sex chromosome system was determined on the basis of the sex chromosome-specific pattern of NORs. Some karyotypes contained several different X chromosomes. In this case, X chromosomes were numbered according to decreasing size. The preferential position of sex chromosomes was evaluated from 10 or 20 chromosome plates. Chromosomes were measured using the programme IMAGEJ 1.47 (http://imagej.nih.gov/ij/). Karyotypes were assembled using the programme Corel Photo Paint X3. In some ninetines (*Kambiwa*) and pholcines (*Metagonia*, *Muruta*, *Nipisa*, *Pehrforsskalia*, *Pholcus bamboutos*), data on chromosome morphology were not sufficient to assemble the karyotype. Karyotypes of *Holocnemus caudatus*, *Pholcus phalangioides*, and *Spermophora senoculata* have already been published in another paper [9]. Finally, several late prophases I (preferably five or ten) were analysed to determine the frequency of chiasmata, which was assessed as a ratio of total chiasmata number found to total bivalent number counted in the analysed plates.

### Detection of nucleolus organizer regions

Nucleolus organizer regions were detected using the biotin labelled 18S rDNA probe. Initially, we tested probes from several spider species. The probe from the haplogyne spider *Dysdera erythrina* (Dysderidae) showed the lowest level of non-specific hybridization and was used in our study. The probe was generated following [65]. The *D. erythrina* 18S rDNA amplicon was about 1600 bp long. It was cloned into the P-Gem T easy vector (Promega, Madison, WI, USA) and Sanger sequenced using both specific [65] and M13 universal primers. We recovered a 1549 bp long sequence, which was deposited in GenBank under the acc. no. MT886274. Blastn search revealed that the sequence partially matches the 18S rDNA sequences of other *D. erythrina* isolates (Acc. nos. KF929034 and KY016439) with identity > 99.9%. The probe was detected by streptavidin-Cy3, with amplification of the signal (biotinylated antistreptavidin, streptavidin-Cy3). Chromosomes were counterstained by DAPI (see [65] for details of method). Besides unstained slides, preparations stained by Giemsa were used for NOR detection after their observation under immersion oil objective. Immersion oil was removed from these preparations by xylene and benzine baths (1 min each, RT). The stain was removed from slides by their incubation in fixative (3 min, RT). In *Artema*, pre-treatment of slides with proteinase K was carried out to remove cytoplasmatic residues (see [65] for details of treatment). Selected chromosome plates were captured with an Olympus IX81 microscope equipped with an ORCA-AG CCD camera (Hammatsu), or Zeiss Axioplan 2 microscope along with F-View CCD camera (Olympus). Images were pseudocoloured (red for Cy3, blue for DAPI) and superimposed with Cell^R software (Olympus Soft Imaging Solutions) or AnalySIS 3.2 software (Soft Imaging, System GmbH, Münster, Germany). Morphology of NOR-bearing pairs was determined using images or inferred from data on the morphology of the chromosome pairs (in case all pairs had the same morphology, e.g. metacentric). In pholcines exhibiting the X_1_X_2_Y system, NOR-bearing sex chromosomes were identified by a combination of two approaches. First, sex chromosomes often exhibited more intensive fluorescence than the other chromosomes during mitosis. Second, from comparison of late prophase/metaphase I and mitotic metaphase, it is possible to distinguish the NOR-bearing chromosomes of the X_1_X_2_Y system. In meiosis, these chromosomes pair by their NOR-bearing regions, thus forming one strong signal comprised of several NORs.

### Evolution of cytogenetic characters

We organized the obtained data into 17 characters describing the number, size, and morphology of chromosomes, SCS, and NORs. The evolution of cytogenetic characters was reconstructed by character mapping on the latest molecular phylogeny of Pholcidae [20, 21] (Figs. 15, 16, 18, Additional file 29: Fig. S23, Additional file 30: Fig. S24, Additional file 31: Fig. S25). To reconstruct karyotype evolution of pholcids, we also used data obtained by other authors (see Additional file 2: Table S2, Additional file 7: Table S3, Additional file 12: Table S4, Additional file 19: Table S5). Doubtful data were excluded from the reconstruction (see “Discussion” for their analysis). The cytogenetic characters used in the reconstruction of karyotype evolution are listed below.

#### Number, size, and morphology of chromosome pairs

*Character 1.* Number of chromosome pairs: (0) 15, (1) 13, (2) 12, (3) 11, (4) 10, (5) 8, (6) 7, (7) 6, (8) 4.

*Character 2.* Relative size of first three chromosome pairs: (0) gradual decrease in size from the first to fourth pair, (1) a considerable increase of chromosomes of the first pair (size difference between chromosome of first and second pair > 1.5% of TCL), (2) first two pairs considerably increased (size difference between chromosome of second and third pair > 1.5% of TCL), (3) first three pairs considerably increased (size difference between chromosome of third and fourth pair > 1.5% of TCL).

*Character 3.* Relative size of the smallest chromosome pair: (0) small size difference between last two pairs, (1) considerable reduction of last pair (size difference between chromosomes of last two pairs > 1.5% of TCL).

*Character 4.* Morphology of longest chromosome pair: (0) metacentric, (1) submetacentric.

*Character 5.* Morphology of long marker chromosome pair: (0) metacentric, (1) submetacentric, (2) subtelocentric.

*Character 6.* Morphology of medium-sized marker chromosome pair: (0) metacentric, (1) submetacentric, (2) subtelocentric, (3) acrocentric.

*Character 7.* Morphology of short marker chromosome pair: (0) metacentric, (1) submetacentric, (3) subtelocentric, (4) acrocentric.

*Character 8.* Morphology of shortest chromosome pair: (0) metacentric, (1) submetacentric, (2) subtelocentric, (3) acrocentric.

In the case of characters 2–8, we did not consider chromosome pairs of species to be homologous except for several specific cases, where we consider some chromosomes of closely related species exhibiting a very similar karyotype to probably be homologous. It concerns the events E1, E2, E3, E24, E27, E28, E29, E34, and E35 (see Additional file 1: Table S1).

#### Sex chromosomes

*Character 9.* Sex chromosome system: (0) X_1_X_2_Y, (1) XY, (2) X_1_X_2_X_3_X_4_Y, (3) X_1_X_2_0, (4) X0, (5) X_1_X_2_X_3_0.

*Character 10.* X_2_ chromosome. (0) large element, similar to X_1_, (1) reduction of X_2_ (size ≤ 7% of TCL for Arteminae, Pholcinae; size ≤ 7.5% of TCL for Smeringopinae), (2) considerable reduction of X_2_ (size ≤ 4% of TCL), (3) considerable increase of X_2_ (≥ 2% of TCL).

*Character 11.* Y chromosome. (0) tiny, (1) increase (size ≥ 4.5% of TCL), (2) considerable increase (size ≥ 7% of TCL).

*Character 12.* X_1_ morphology: (0) metacentric, (1) submetacentric.

*Character 13.* X_2_ morphology: (0) metacentric, (1) submetacentric, (2) subtelocentric, (3) acrocentric.

*Character 14.* Y morphology: (0) metacentric, (1) submetacentric.

#### Nucleolus organizer regions

*Character 15.* Total number of NOR loci on chromosome pairs: (0) 1, (1) 2, (2) 3, (3) 4, (4) 5.

*Character 16.* Location of NORs on chromosome pairs: (0) each NOR-bearing pair with one terminal NOR, (1) pericentric, (2) each NOR-bearing pair with one terminal NOR, except for one pair with NOR at both ends.

*Character 17.* Sex chromosome-linked NORs: (0) NORs located exclusively on chromosome pairs, (1) NOR at one end of X_1_ chromosome (X_1_X_2_Y system), (2) NOR at both ends of X_1_ chromosome (X_1_X_2_Y system), (3) NOR at both ends of X_1_ chromosome plus one end of X_2_ chromosome (X_1_X_2_Y system), (4) NOR at both ends of X_1_ chromosome and X_2_ chromosome, as well as at one end of Y chromosome (X_1_X_2_Y system), (5) NOR at one X chromosome end (X0 system), (6) NOR at both X chromosome ends (X0 system), (7) X chromosome-linked NOR (X_1_X_2_X_3_X_4_Y system), (8) loss of all sex chromosome-linked NORs.

## Supplementary Information

**Additional file 1: Table S1.** Species under study, male karyotype data (including standard deviation). Abbreviations: EX = product of event X (X = number of event), phase = phase of mitotic/meiotic division used to obtain data on chromosome morphology (m mit = mitotic metaphase, m I = metaphase I, m II = metaphase II), plates = number of chromosome plates evaluated, parameters = parameters used to describe chromosome morphology [CI = centromeric index, RCL = relative chromosome length (% of TCL)]. Chromosome morphology is indicated by background colour of a box (pink: metacentric, brown: submetacentric, dark blue: subtelocentric, light blue: acrocentric, red: unknown).

**Additional file 2: Table S2.** Arteminae, summary of male cytogenetic data, including results of other authors. Doubtful data are not included. See database [24] for full list of published data on pholcid karyotypes, including doubtful data. Abbreviations: a = acrocentric, bi = biarmed, CP = chromosome pair, m = metacentric, n = number of plates evaluated, p = short chromosome arm, pc = pericentric, q = long chromosome arm, SC = sex chromosome, SCS = sex chromosome system, sm = submetacentric, ® = revision of data of other authors, st = subtelocentric, t = terminal, ? = unknown, *X = data of other authors (X = reference number).

**Additional file 3: Fig. S1.** Arteminae, *Artema atlanta*, male karyotype, stained by Giemsa. Based on two sister metaphases II. Chromosomes metacentric, except for four submetacentric pairs (nos 1, 6, 9, 12) and subtelocentric X_2_ chromosome. Note low condensation of X chromosomes. Bar = 10 μm.

**Additional file 4: Fig. S2.** Sex chromosomes of artemines with the X_1_X_2_Y and XY systems. Stained by Giemsa. X_1_ = X_1_ chromosome, X_2_ = X_2_ chromosome, Y = Y chromosome. (**a**–**c**) *Artema atlanta* (X_1_X_2_Y). **a** Metaphase I, composed of 15 bivalents and sex chromosome trivalent. **b** Metaphase II, with chromosomes X_1_ and X_2_ at the periphery of the plate. **c** Metaphase II with Y chromosome. (**d**–**f**) *Wugigarra* sp. **d** Metaphase I, consisting of seven bivalents and a XY pair. **e** Metaphase II containing a positively heteropycnotic X chromosome (n = 8). **f** Metaphase II, containing a Y chromosome (n = 8). Bar = 10 μm.

**Additional file 5: Fig. S3.** Sex chromosomes of artemines with the X0 system. Stained by Giemsa. X = X chromosome. (**a**, **b**) *Chisosa diluta*. **a** Metaphase I, consisting of six bivalents and a peripheral X chromosome. **b** Group of metaphases II separated by lines. It consists of one metaphase containing a positively heteropycnotic X chromosome (n = 7, in the middle of the plate) and two metaphases without sex chromosome (left metaphase is incomplete); (**c**, **d**) *Holocneminus* sp. **c** Metaphase I, comprising seven bivalents and peripheral X chromosome. **d** Two sister metaphases II separated by a line (n = 8 including X chromosome + n = 7); (**e**, **f**) *Physocyclus dugesi*. **e** Diplotene, comprising seven bivalents and peripheral X chromosome. **f** Anaphase II. Note slight positive heteropycnosis of X chromosome. Bar = 10 μm.

**Additional file 6: Fig. S4.** Arteminae, males, detection of NORs (FISH). *Arrowhead* = NOR-bearing chromosome (**b**, **c**) or bivalent (**a**, **d**), X = X chromosome, Y = Y chromosome. (**a**, **b**) *Artema atlanta* (X_1_X_2_Y). **a** Metaphase I, note NOR-bearing bivalent. **b** Two fused sister metaphases II, one pair of submetacentric chromosomes bears a terminal NOR; **c** *Wugigarra* sp. (XY), two fused sister metaphases II. Note chromosomes of a subtelocentric pair bearing terminal NOR at the end of short arm; **d** *Chisosa diluta* (X0), diffuse stage, one bivalent includes a NOR. Bar = 10 μm.

**Additional file 7: Table S3.** Modisiminae and Ninetinae, summary of male cytogenetic data, including results of other authors. Doubtful data are not included. See database [24] for full list of published data on pholcid karyotypes, including doubtful data. Abbreviations: bi = biarmed, CP = chromosome pair, m = metacentric, n = number of plates evaluated, p = short chromosome arm, q = long chromosome arm, SC = sex chromosome, SCS = sex chromosome system, sm = submetacentric, st = subtelocentric, t = terminal, ? = unknown, *X = data of other authors (X = reference number).

**Additional file 8: Fig. S5.** Sex chromosome systems of modisimines. Stained by Giemsa. X = X chromosome. (**a**–**c**) *Anopsicus* sp. (X0) **a** metaphase I, consisting of eight bivalents and peripheral X chromosome. **b** Metaphase II, including X chromosome (n = 9). **c** Metaphase II, without X chromosome (n = 8); (**d**, **e**) *Modisimus* cf. *elongatus* (X0). **d** Diplotene, comprising eight bivalents and a peripheral X chromosome. **e** Two sister metaphases II separated by a line (n = 8 + n = 9, including peripheral X chromosome); (**f**, **g**) *Psilochorus pallidulus* (X0). **f** Diplotene, comprising eight bivalents and peripheral X chromosome. **g** Two sister metaphases II (n = 9, including X chromosome + n = 8). Bar = 10 μm.

**Additional file 9: Fig. S6.** *Psilochorus* (Modisiminae), male karyotypes, stained by Giemsa. Sex chromosome is the longest element of the karyotype. **a** *Psilochorus californiae*. Karyotype metacentric, except for two submetacentric (nos 2, 7) and one subtelocentric pairs (no. 5). Based on spermatogonial metaphase, centromeres marked by arrowheads; **b** *P. pallidulus*. Chromosomes metacentric. Based on two sister metaphases II. Bar = 10 μm.

**Additional file10: Fig. S7.** Sex chromosome systems of ninetines. Stained by Giemsa. Figures **a** and **b** contain a scheme of the multivalent. M = sex chromosome multivalent, O = overlapping of two bivalents, X = X chromosome, X_1_ = X_1_ chromosome, X_2_ = X_2_ chromosome, Y = Y chromosome. (**a**, **b**) *Kambiwa neotropica* (X_1_X_2_X_3_X_4_Y), plates of the first meiotic division consisting of 12 bivalents plus a sex chromosome multivalent consisting of four “arms”. Two “arms” are thick (red) and two are thin (orange). **a** Diakinesis. **b** Metaphase I. Note cross-shaped morphology of multivalent; (**c**, **d**) *Pholcophora americana* (X_1_X_2_Y). **c** Diakinesis, comprising 13 bivalents and sex chromosomes X_1_, X_2_, and Y. Sex chromosomes show end-to-end paring. **d** Two sister metaphases II separated by a line (n = 15, including chromosomes X_1_ and X_2_ + n = 14 including Y microchromosome). Bar = 10 μm.

**Additional file 11: Fig. S8.** Cytogenetics of ninetines (**a**–**c**) and pholcines (**d**–**i**), male germline. Figures **d**, **f**, **i** contain scheme of sex chromosome trivalent X_1_X_2_Y. B = large bivalent, H = large bivalent exhibiting positive heteropycnosis, mon = monoarmed X chromosome, T = sex chromosome trivalent, X = X chromosome, X_1_ = X_1_ chromosome, X_2_ = X_2_ chromosome, Y = Y chromosome. (**a**–**c**) *Kambiwa neotropica* (X_1_X_2_X_3_X_4_Y). **a** Spermatogonial metaphase (2n = 29). Chromosomes are biarmed, except for two monoarmed chromosomes. **b** Metaphase II, consisting of 12 chromosomes and cluster of four positively heteropycnotic X chromosomes. **c** Metaphase II, formed by 12 chromosomes and a Y microchromosome; **d** *Aetana kinabalu* (X_1_X_2_Y), incomplete metaphase I. Sex chromosomes pair by ends of their arms, X chromosomes are positively heteropycnotic; (**e**, **f**) *Metagonia* sp. (X_1_X_2_Y), late prophase I. Note low chromosome condensation. **e** Diplotene. Plate consists of eight bivalents and almost decondensed sex chromosomes. **f** Diakinesis, note the X_1_X_2_Y trivalent, Y chromosome more condensed than X chromosomes; (**g**, **h**) *Pehrforsskalia conopyga* (X_1_X_2_Y). **g** Early diplotene. Note sex chromosomes forming a compact positively heteropycnotic body and large positively heteropycnotic bivalent. **h** Metaphase I, formed by seven bivalents and sex chromosome trivalent. Two bivalents (B) are much longer than the remaining ones. Note tiny Y chromosome; **i** *Pholcus bamboutos* (X_1_X_2_Y), transition from metaphase to anaphase I. Note the delayed separation of sex chromosomes. Only one end of the X_2_ chromosome takes part in pairing. The Y chromosome is positively heteropycnotic. Bar = 10 μm.

**Additional file 12: Table S4.** Pholcinae, summary of male cytogenetic data, including results of other authors. Doubtful data are not included. See database [24] for full list of published data on pholcid karyotypes, including doubtful data. Abbreviations: a = acrocentric, bi = biarmed, CP = chromosome pair, m = metacentric, n = number of plates evaluated, p = short chromosome arm, q = long chromosome arm, SC = sex chromosome, SCS = sex chromosome system, sm = submetacentric, ® = revision of data of other authors, st = subtelocentric, t = terminal, ? = unknown, *X = data of other authors (X = reference number).

**Additional file 13: Fig. S9.** Sex chromosomes of pholcines with the X_1_X_2_Y system, part I. Stained by Giemsa. Figures **a**, **d**, **e** contain a scheme of the sex chromosome trivalent. X_1_ = X_1_ chromosome, X_2_ = X_2_ chromosome, Y = Y chromosome, PS = precocious separation of chromosomes of the bivalent. (**a**–**c**) *Aetana kinabalu*. **a** Metaphase I, comprising 11 bivalents (one bivalent shows a precocious separation of chromosomes) and a sex chromosome trivalent. X chromosomes are positively heteropycnotic. **b** Metaphase II, containing X chromosomes (n = 12). **c** Transition metaphase II/anaphase II, fusion of two sister plates. Note X chromosomes exhibiting a delayed separation of chromatids and a Y microchromosome; (**d**–**f**) *Nipisa deelemanae*. **d** Metaphase I, comprising 11 bivalents and sex chromosome trivalent. **e** Part of a plate formed by several fused metaphases I, sex chromosome trivalent encircled. **f** Two sister metaphases II separated by a line (n = 12, including Y microchromosome + n = 13 including metacentric chromosomes X_1_ and X_2_). Note the reduction of the X_2_ chromosome. Bar = 10 μm.

**Additional file14: Fig. S10.** Sex chromosomes of pholcines with the X_1_X_2_Y system, part II. Stained by Giemsa. Figures **a**, **b** contain a scheme of the sex chromosome trivalent. T = trivalent, X_1_ = X_1_ chromosome, X_2_ = X_2_ chromosome, Y = Y chromosome. (**a**–**d**) *Leptopholcus guineensis*. **a** Early diplotene, consisting of seven bivalents and a sex chromosome trivalent, X chromosomes exhibit a low condensation. **b** Two fused diplotene. **c** Metaphase II, including X chromosomes (n = 9). X1 chromosome is positively heteropycnotic. **d** Metaphase II, including a Y chromosome (n = 8); (**e**, **f**) *Metagonia* sp. **e** Spermatogonial metaphase, note the Y microchromosome. **f** Diplotene, note the positively heteropycnotic body formed by the sex chromosomes. Bar = 10 μm.

**Additional file15: Fig. S11.** Sex chromosomes of pholcines with the X_1_X_2_Y system, part III. Stained by Giemsa. Figures **a**, **c**, **e** contain a scheme of the sex chromosome trivalent. c = centromere, X_1_ = X_1_ chromosome, X_2_ = X_2_ chromosome, Y = Y chromosome. (**a**, **b**) *Muruta tambunan*. **a** Diakinesis, consisting of 11 bivalents and a sex chromosome trivalent. **b** Two sister metaphases II separated by a line (n = 12, including Y chromosome + n = 13 including chromosomes X_1_ and X_2_). Note the metacentric X_1_ chromosome and submetacentric X_2_ chromosome on the periphery of the plate. They exhibit positive heteropycnosis; (**c**, **d**) *Pholcus phalangioides*. **c** Diakinesis, comprising 11 bivalents and a sex chromosome trivalent, which is placed in the middle of the plate and exhibits positive heteropycnosis. Concerning the X_2_ chromosome, only end of the long arm is involved in pairing. **d** Anaphase II. Note the positive heteropycnosis of the sex chromosomes. The X chromosomes are associated; (**e**, **f**) *Spermophora senoculata*. **e** Metaphase I, comprising 11 bivalents and a sex chromosome trivalent. **f** Metaphase II, X chromosomes are less condensed than the other chromosomes; (**g**, **h**) *Quamtana hectori*. **g** Metaphase I, composed of 10 bivalents and a sex chromosome trivalent. Concerning the X_2_ chromosome, only one end is involved in pairing. **h** Two sister metaphases II separated by a line (n = 11 including Y chromosome + n = 12 including chromosomes X_1_ and X_2_). Bar = 10 μm.

**Additional file 16: Fig. S12.** Pholcinae, male karyotypes, Giemsa staining. Based on metaphase II (**a**) or two sister metaphases II (**b**–**d**). **a** *Aetana kinabalu*, haploid set, karyotype metacentric except for submetacentric X_1_ chromosome and acrocentric chromosome (no. 10), which is considerably reduced in comparison with preceding chromosome. The Y chromosome is from another metaphase II. Morphology of the Y chromosome is unresolved; **b** *Pholcus pagbilao*. Karyotype is metacentric except for three submetacentric pairs (nos 5, 7, 10), submetacentric Y chromosome, and acrocentric X_2_; **c** *P. opilionoides*, chromosomes metacentric except for five submetacentric pairs (nos 2–6) and acrocentric X_2_. Chromosome X_1_ is the longest chromosome of karyotype. On the contrary, Y is the smallest one. Y chromosome is replaced by Y from another plate; **d** *Quamtana hectori*. Karyotype metacentric, except for acrocentric X_2_. Centromeres of sex chromosomes marked by arrowheads. Bar = 10 μm.

**Additional file 17: Fig. S13.** Sex chromosomes of pholcines with the X0 system. Stained by Giemsa. X = X chromosome. (**a**, **b**) *Belisana sabah*. **a** Metaphase I, consisting of 11 bivalents and a peripheral X chromosome. **b** Two sister metaphases II separated by a line (n = 11 + n = 12, including a positively heteropycnotic X chromosome); (**c**, **d**) *Cantikus sabah*. **c** Diplotene, comprising six bivalents and a positively heteropycnotic X chromosome placed on the periphery of the plate. **d** Prometaphase II including the X chromosome (n = 7); (**e**, **f**) *Micropholcus fauroti*. **e** Diplotene composed of four bivalents and a positively heteropycnotic X chromosome placed on the periphery of the plate. **f** Plate formed by fusion of two sister metaphases II. It includes a negatively heteropycnotic X chromosome. Bar = 10 μm.

**Additional file 18: Fig. S14.** Pholcinae, males, detection of NORs (FISH). Figures **c**, **e** contain a scheme of the sex chromosome trivalent X_1_X_2_Y (red = signal formed by several NORs). Arrowhead = NOR-bearing chromosome (**a**, **b**, **d**, **f**–**h**), bivalent (**c**, **e**) or trivalent (**c**, **e**), open arrowhead = sex chromosome-linked NOR, T = sex chromosome trivalent, X_1_ = X_1_ chromosome, X_2_ = X_2_ chromosome, Y = Y chromosome, + = signal formed by several NORs. **a** *Micropholcus fauroti* (X0), mitotic metaphase. Note association of two homologous chromosomes containing terminal NOR (in – interphase nucleus); (**b**, **c**) *Nipisa deelemanae* (X_1_X_2_Y). **b** Mitotic metaphase. X chromosomes (X_1_, X_2_) and another chromosome (II) bear a terminal NOR at both ends. Five other chromosomes, including Y chromosome, involves one terminal NOR only. The sex chromosomes X_1_ and X_2_ are associated in parallel in the middle of the plate. **b** Metaphase I, note the three bivalents bearing a NOR and the sex chromosome trivalent with a signal in the region of chromosome pairing (see scheme); (**d**–**f**) *Quamtana hectori* (X_1_X_2_Y). **d** Mitotic metaphase (separated by a line from another plate). X_1_ chromosome bears two NORs, each at opposite end of the chromosome. Chromosomes of two pairs also include a terminal NOR; **e** Metaphase I, two bivalents contain NOR. The sex chromosome trivalent contains a signal in region of chromosome pairing (see scheme). **f** Plate formed by fused sister metaphases II, chromosomes of NOR-bearing pairs exhibit biarmed morphology. The X_1_ chromosome is terminated by NOR at both ends. Y chromosome considerably condensed, without signal; (**g**, **h**) *Q. filmeri* (X_1_X_2_Y), mitotic plates. The X_1_ chromosome bears two NORs, each at opposite end of chromosome. Chromosomes of one pair also contain a terminal NOR. **g** Prophase, sex chromosomes exhibit a more intensive fluorescence than the other chromosomes. **h** Metaphase. Bar = 5 μm except for **c**, **e**, **g** (10 μm).

**Additional file 19: Table S5.** Smeringopinae, summary of male cytogenetic data, including results of other authors. Doubtful data are not included. See database [24] for full list of published data on pholcid karyotypes including doubtful data. Abbreviations: a = acrocentric, bi = biarmed, CP = chromosome pair, m = metacentric, n = number of plates evaluated, p = short chromosome arm, q = long chromosome arm, SC = sex chromosome, SCS = sex chromosome system, sm = submetacentric, ® = revision of data of other authors, st = subtelocentric, t = terminal, ? = unknown, *X = data of other authors (X = reference number).

**Additional file 20: Fig. S15.** *Hoplopholcus* and *Smeringopus* (Smeringopinae), male karyotypes, stained by Giemsa. Based on metaphase II (**a**) or two sister metaphases II (**b**–**e**). Autosome pairs decrease gradually in size. The X_1_ is the longest element of the set (except for **d**). Karyotypes are predominated by metacentrics. **a** *H. labyrinthi*, haploid set, note the two submetacentric chromosomes (nos 5, 9), subtelocentric chromosome (no. 6) and subtelocentric X_2_ chromosome; **b** *S. atomarius*, note one submetacentric (no. 3) and one acrocentric pairs (no. 13), and submetacentric X_2_; **c** *S. ndumo*, note two submetacentric pairs (nos 4, 11); **d** *S. peregrinus*, note three submetacentric (nos 1, 4, 6) and one subtelocentric pairs (no. 12), and subtelocentric X_2_. Sex chromosomes positively heteropycnotic; **e** *Smeringopus* sp., note two submetacentric pairs (nos 1, 2), one acrocentric pair (no. 10) and acrocentric X_2_. Bar = 10 μm.

**Additional file 21: Fig. S16.** *Crossopriza lyoni* (Smeringopinae), male karyotype, Giemsa staining. Based on two sister metaphases II. Karyotype metacentric, except for submetacentric pairs nos 2 and 11. First two pairs differ from the other ones by large size. The X chromosome is the longest chromosome of the set. It is slightly positively heteropycnotic. Bar = 10 μm.

**Additional file 22: Fig. S17.** Sex chromosomes of smeringopines with multiple X chromosomes. Stained by Giemsa. X = X chromosome, X_1_ = X_1_ chromosome, X_2_ = X_2_ chromosome. (**a**, **b**) *Hoplopholcus forskali* (X_1_X_2_0). **a** Diakinesis, composed of 13 bivalents and two X chromosomes, note the end-to-end association of the X chromosomes. **b** Telophase I, half plate containing X chromosomes; (**c**–**e**) *Smeringopus ndumo* (X_1_X_2_0). **c** Diakinesis, comprising 13 bivalents and two X chromosomes. **d** Metaphase II, containing X chromosomes (n = 15). **e** Metaphase II, without the sex chromosomes (n = 13); (**f**–**h**) *S. pallidus* (X_1_X_2_X_3_0). **f** Diakinesis, composed of 13 bivalents and three X chromosomes, sex chromosomes grouped in the middle of the plate. **g** Metaphase II with X chromosomes (n = 16). X chromosomes are associated at the periphery of the plate. They exhibit a slight positive heteropycnosis. **h** Metaphase II, without sex chromosomes (n = 13). Bar = 10 μm.

**Additional file 23: Fig. S18.** Sex chromosomes of smeringopines with the X0 system. Stained by Giemsa. X = X chromosome. (**a**, **b**) *Crossopriza lyoni*. **a** Metaphase I composed of 11 bivalents and X chromosome. **b** Anaphase I; (**c**, **d**) *Holocnemus pluchei*. **c** Metaphase I, consisting of 13 bivalents and an X chromosome. The X chromosome is placed at the periphery of the plate. Note the association of terminal parts of the X chromosome arms. **d** Metaphase II, including the X chromosome. This element is slightly positively heteropycnotic; (**e**, **f**) *Stygopholcus skotophilus*. **e** Diplotene, composed of 11 bivalents and an X chromosome. **f** Plate formed by two fused sister metaphases II, 2n = 23. Note the positively heteropycnotic X chromosome. Bar = 10 μm.

**Additional file 24: Fig. S19.** Pholcidae, male germline, behaviour of sex chromosomes prior to meiosis. *Arrow* = sex chromosomes, X_1_ = X_1_ chromosome, X_2_ = X_2_ chromosome, Y = Y chromosome. (**a**, **b**) *Muruta tambunan* (X_1_X_2_Y), mitotic metaphase, chromosomes X_1_, X_2_, and Y positively heteropycnotic. Chromosomes X_2_ and Y are approximately of the same size. **a** Sex chromosomes grouped in the middle of the plate. **b** Chromosomes X_1_ and X_2_ associated in parallel, Y chromosome released from the association; **c** *Artema nephilit* (X_1_X_2_Y), early mitotic metaphase, X chromosomes are marked by a dotted line. They are associated in parallel in the middle of the plate. Their condensation is slightly delayed in comparison with the other chromosomes; **d** *Hoplopholcus cecconii* (X_1_X_2_0), transition from mitotic metaphase to anaphase. Chromosome X_1_ is placed in the middle of the plate; (**e**, **f**) *Pholcus kindia* (X_1_X_2_Y). **e** Premeiotic interphase. X chromosomes pair in parallel in the middle of the nucleus. The Y chromosome does not take part in pairing. **f** Preleptotene. Sex chromosomes are associated in the middle of the nucleus, the Y chromosome is more condensed than the X chromosomes. Bar = 10 μm.

**Additional file 25: Fig. S20.** Pholcidae, male meiosis, condensation and segregation of sex chromosomes. X = X chromosome, Xs = X chromosomes, X_1_ = X_1_ chromosome, X_2_ = X_2_ chromosome, Y = Y chromosome. **a** *Pholcus kindia* (X_1_X_2_Y), early diplotene. Y chromosome highly condensed. In contrast, X chromosomes almost decondensed; **b** *Hoplopholcus cecconii* (X_1_X_2_0), late anaphase I. X chromosomes are arranged in parallel and less condensed than the other chromosomes. Moreover, their segregation and separation of their chromatids are delayed. Centromeres of sex chromosomes are formed by a prominent knob; **c** *Cantikus sabah* (X0), diplotene. Sex chromosome forms a highly condensed body; **d** *Hoplopholcus forskali* (X_1_X_2_0), plate formed by fusion of 1) two sister late prometaphases II (left) and 2) two sister early prometaphases II (right). In contrast to autosomes, sex chromosomes differ considerably by condensation in early and late prometaphase II. They are almost decondensed during early prometaphase II (right); **e** *Holocneminus* sp. (X0), plate formed by two sister prometaphases II, sex chromosome forms a highly condensed body; **f** *Micropholcus fauroti* (X0), two fused sister metaphases II. Sex chromosome shows precocious division; **g** *Aetana kinabalu* (X_1_X_2_Y), late metaphase II, division of X chromosomes is delayed; **h** *Pholcus* sp. (X_1_X_2_Y), two half-plates of anaphase II containing positively heteropycnotic Y chromosome in the middle; **i** *Psilochorus simoni* (X0), two sister anaphases II. Segregation of X chromosome delayed. This element is slightly positively heteropycnotic at right anaphase II. Bar = 10 μm.

**Additional file 26: Fig. S21.** Arteminae, *Wugigarra* sp., male meiosis, behaviour of sex chromosomes. X = X chromosome, Y = Y chromosome, II = bivalent containing two chiasmata. **a** Metaphase I, three bivalents include two chiasmata. Pairing of metacentric chromosomes X and Y is ensured by ends of their arms. **b** Two sister metaphases II (separated by dashed line). While X chromosome is placed at the periphery of one plate, Y chromosome is in middle of another plate. Note positive heteropycnosis of sex chromosomes. Bar = 10 μm.

**Additional file 27: Fig. S22.** *Pholcus* sp. (X_1_X_2_Y), testes, endopolyploid nucleus. Heterochromatic body in the middle of the nucleus is formed by sex chromosomes (arrow). Inset: a standard diploid nucleus. Bar = 5 μm.

**Additional file 28: Appendix S1.** Evolution of particular chromosomes of the pholcid X_1_X_2_Y system.

**Additional file 29: Fig. S23.** Arteminae, Modisiminae, and Ninetinae: characters of NORs and sex chromosomes mapped on a cladogram. Based on the most recent molecular phylogeny of Pholcidae [20, 21]. Standard continuous lines: low bootstrapping branch support, i.e. < 70%; thick continuous lines: modest to full support of hypotheses, ≥ 70% [21]; discontinuous lines: taxa not included in molecular phylogeny - phylogenetic position is based on cytogenetic data (dashed lines) or unresolved (dotted lines). Number above mark: number of character; number below mark: character state. Full marks: characters without homoplasy; empty marks: homoplasic characters. In some species, data on some characters are missing (information in square brackets). The reconstruction of karyotype evolution is based on our data, except for taxa marked by the formula RX (see reference X for information). See Methods (pp. 32–33) for the coding of characters.

**Additional file 30: Fig. S24.** Smeringopinae: karyotype characters mapped on a cladogram. Based on the most recent molecular phylogeny of Pholcidae [20, 21]. Standard continuous lines: low bootstrapping branch support, i.e. < 70%; thick continuous lines: modest to full support of hypotheses, ≥ 70% [21]; discontinuous lines: taxa not included in molecular phylogeny - phylogenetic position is based on other sources [79] and/or on cytogenetic data (dashed lines) or unresolved (dotted lines). Number above mark: number of character; number below mark: character state. Full marks: characters without homoplasy; empty marks: homoplasic characters. In some species, data on some characters are missing (information in square brackets). The reconstruction of karyotype evolution is based on our data, except for taxa marked by the formula (RX) (some information taken from reference X). See Methods (pp. 32–33) for coding of the characters.

**Additional file 31: Fig. S25.** Pholcinae: characters of NORs and sex chromosomes mapped on a cladogram. Based on the most recent molecular phylogeny of Pholcidae [20, 21]. Standard continuous lines: low bootstrapping branch support, i.e. < 70%; thick continuous lines: modest to full support of hypotheses, ≥ 70% [21]; dashed lines: taxa not included in molecular phylogeny - phylogenetic position is based on cytogenetic data. Number above mark: number of character; number below mark: character state. Full marks: characters without homoplasy; empty marks: homoplasic characters. In some species, data on some characters are missing (information in square brackets). The reconstruction of karyotype evolution is based on our data, except for taxa marked by the formula RX (see reference X for information). See Methods (pp. 32–33) for coding of the characters.

**Additional file 32: Table S6.** Species studied, their instar, sex, collecting data and depositories. Abbreviations: AM = Australian Museum, Sydney, Australia (specimen KS 128687), Co. = county, Hwy = highway, Isl. = island, m = male, Mts. = mountains, N = north, N.P. = national park, NW = northwest, S = south, SL = specimens lost or discarded, sm = subadult male, W = west, ZFMK = Zoological Research Museum Alexander Koenig.

## Data Availability

All supporting data are provided in the additional files. The partial 18S rDNA sequence of *Dysdera erythrina* was deposited in GenBank under the Acc. no. MT886274.

## Abbreviations

AT: Adenine–thymine
CAS: Czech Academy of Science
CCD: Charge-coupled device
CHS clade: Clade formed by *Crossopriza*, *Holocnemus*, and *Stygopholcus*
CI: Centromeric index
CP: Chromosome pair
CSCP: Cryptic sex chromosome pair
Cy-3: Cyanomycin 3
DAPI: 4′,6-Diamidine-2′-phenylindole dihydrochloride
FISH: Fluorescence in situ hybridization
KCl: Kalium chloride
NCPs: Number of chromosome pairs
NOR: Nucleolus organizer region
RCL: Relative chromosome length
rDNA: Ribosomal DNA
rRNA: Ribosomal RNA
RT: Room temperature
S: Svedberg unit
SCB: Sex chromosome body
SCL-NOR clade: Sex chromosome-linked NOR clade (belonging to Pholcinae)
SCS: Sex chromosome system
TCL: Total chromosome length
2n: Diploid chromosome number
