# Correction to: Evolutionary pattern of karyotypes and meiosis in pholcid spiders (Araneae: Pholcidae): implications for reconstructing chromosome evolution of araneomorph spiders

**DOI:** 10.1186/s12862-021-01828-3

**Published:** 2021-05-21

**Authors:** Ival M. vila Herrera, Ji Krl, Markta Pastuchov, Martin Forman, Jana Musilov, Tereza Konkov, Frantiek hlavsk, Magda Zrzav, Petr Nguyen, Pavel Just, Charles R. Haddad, Maty Himan, Martina Koubov, David Sadlek, Bernhard A. Huber

**Affiliations:** 1 Laboratory of Arachnid Cytogenetics, Department of Genetics and Microbiology, Faculty of Science, Charles University, Vinin 5, 128 44 Prague 2, Czech Republic; 2 Research Team of Plant Stress Biology and Biotechnology, Division, of Crop Genetics and Breeding, Crop Research Institute, Drnovsk 507/73, 161 00 Prague 6, Czech Republic; 3 Prague 1, Czech Republic; 4 Invertebrate Zoology Unit, Department of Zoology, Faculty of Science, Charles University, Vinin 7, 128 44 Prague 2, Czech Republic; 5 Laboratory of Molecular Cytogenetics, Department of Molecular Biology and Genetics, Faculty of Science, University of South Bohemia, Braniovsk 31, 370 05 esk Budjovice, Czech Republic; 6 Laboratory of Molecular Cytogenetics, Department of Molecular Biology and Genetics, Institute of Entomology, Biology Centre CAS, Braniovsk 31, 370 05 esk Budjovice, Czech Republic; 7 Research Group of Arachnid Systematics and Ecology, Department of Zoology and Entomology, Faculty of Natural and Agricultural Sciences, University of the Free State, P.O. Box 339, Bloemfontein, 9300 Republic of South Africa; 8 Arachnida Section, Alexander Koenig Zoological Research Museum, Adenauerallee 160, 53113 Bonn, Germany

## Correction to: BMC Ecol Evol (2021) 21:75 https://doi.org/10.1186/s12862-021-01750-8

Following the publication of the original article [1], the authors notified us that Figure 16 contained a few incorrect question marks. These have been removed.

The original article has been corrected.
