# Supplementary material for: Evolutionary pattern of karyotypes and meiosis in pholcid spiders (Araneae: Pholcidae): implications for reconstructing chromosome evolution of araneomorph spiders
Source: BMC Ecol Evol. 2021 May 3;21:75. doi: 10.1186/s12862-021-01750-8 (PMC8091558; doi:10.1186/s12862-021-01750-8)
Supplement: Supplementary file 2 — Additional file 2: Table S2. Arteminae, summary of male cytogenetic data, including results of other authors. Doubtful data are not included. See database [24] for full list of published data on pholcid karyotypes, including doubtful data. Abbreviations: a = acrocentric, bi = biarmed, CP = chromosome pair, m = metacentric, n = number of plates evaluated, p = short chromosome arm, pc = pericentric, q = long chromosome arm, SC = sex chromosome, SCS = sex chromosome system, sm = submetacentric, ® = revision of data of other authors, st = subtelocentric, t = terminal, ? = unknown, *X = data of other authors (X = reference number). [file 12862_2021_1750_MOESM2_ESM.doc]

| **Taxon** | **2n** | **SCS** | **Chromosome pairs:**  **number, morphology** | **Sex chromosome**  **morphology** | **NOR number**  **(CP/SC)** | **NOR-bearing CPs: number,**  **morphology (NOR location)** | **NOR-bearing sex chromosomes:**  **chromosome, morphology (NOR location)** | **Chiasma**  **frequency (n)** |
| --- | --- | --- | --- | --- | --- | --- | --- | --- |
| **Arteminae** |  |  |  |  |  |  |  |  |
| *Artema atlanta* ® | 33 | X_1_X_2_Y | 11m+4sm | X_1_m+X_2_st+Ym | 1/0 | 1 sm (p, t) |  | 1.03 (5) |
| *A. nephilit* | 33 | X_1_X_2_Y | 8m+5sm+1st+1a | X_1_m+X_2_m+Y? | 1/0 | 1 sm (p, t) |  | allmost all biv. 1 chia. (5) |
| *Chisosa diluta* | 13 | X0 | 6m | Xm | 1/0 | 1 m (?) |  | 1.79 (4) |
| *Holocneminus* sp. (Phi9) | 15 | X0 | 5m+1sm+1st | Xm | 1/0 | 1 m (q, t) |  | 1.17 (5) |
| *Physocyclus californicus**^84^ | 15 | X0 | 7m | Xm |  |  |  |  |
| *P. dugesi* | 15 | X0 | 3m+1sm+3st | Xm | 1/2 | 1 sm (p, t) | X, m (1NOR p, t + 1NOR q, t) | 1.70 (10) |
| *P. enaulus**^84^ | 15 | X0 | 7m | Xm |  |  |  |  |
| *P. globosus**^30^ | 15 | X0 | 3m+4sm | Xm |  | 1sm (q, pc) |  |  |
| *P. mexicanus**^31^ | 15 | X0 | 7bi | Xbi |  |  |  |  |
| *Physocyclus* sp.*^85^ | 15 | X0 |  |  |  |  |  |  |
| *Physocyclus* sp.*^84^ | 15 | X0 | 7m | Xm |  |  |  |  |
| *Wugigarra* sp. | 16 | XY | 6m+1st | Xm+Ym | 1/0 | 1 st (p, t) |  | 1.76 (5) |

**Table S2** Arteminae, summary of male cytogenetic data, including results of other authors. Doubtful data are not included. See database [24] for full list of published data on pholcid karyotypes, including doubtful data. Abbreviations: a = acrocentric, bi = biarmed, CP = chromosome pair, m = metacentric, n = number of plates evaluated, p = short chromosome arm, pc = pericentric, q = long chromosome arm, SC = sex chromosome, SCS = sex chromosome system, sm = submetacentric, ® = revision of data of other authors, st = subtelocentric, t = terminal, ? = unknown, *^X^ = data of other authors (^X^ = reference number).
