# Supplementary figures and images for: Evolutionary pattern of karyotypes and meiosis in pholcid spiders (Araneae: Pholcidae): implications for reconstructing chromosome evolution of araneomorph spiders
Source: BMC Ecol Evol. 2021 May 3;21:75. doi: 10.1186/s12862-021-01750-8 (PMC8091558; doi:10.1186/s12862-021-01750-8)

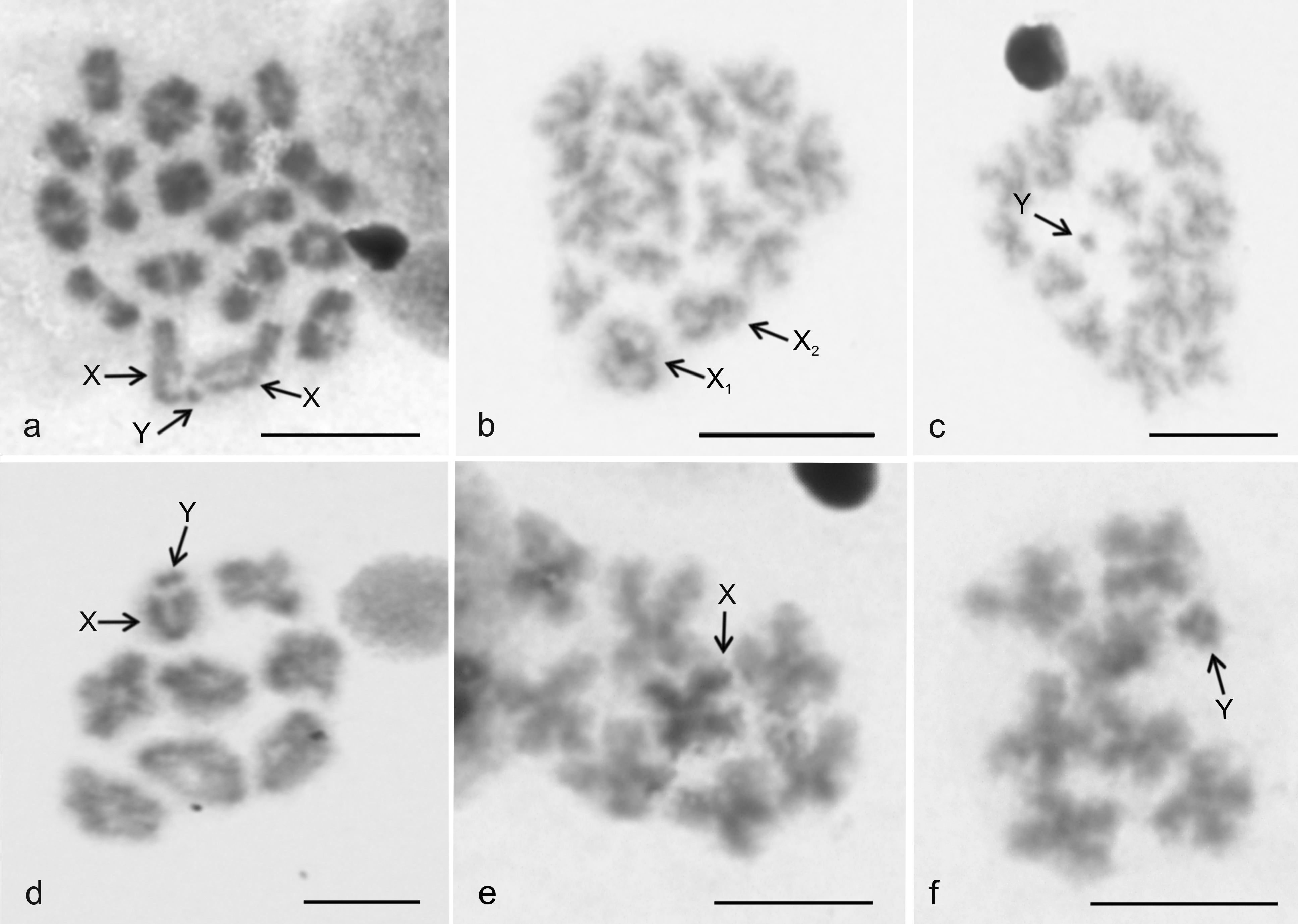

Supplement: Supplementary file 4 — Additional file 4: Fig. S2. Sex chromosomes of artemines with the X1X2Y and XY systems. Stained by Giemsa. X1 = X1 chromosome, X2 = X2 chromosome, Y = Y chromosome. (a–c) Artema atlanta (X1X2Y). a Metaphase I, composed of 15 bivalents and sex chromosome trivalent. b Metaphase II, with chromosomes X1 and X2 at the periphery of the plate. c Metaphase II with Y chromosome. (d–f) Wugigarra sp. d Metaphase I, consisting of seven bivalents and a XY pair. e Metaphase II containing a positively heteropycnotic X chromosome (n = 8). f Metaphase II, containing a Y chromosome (n = 8). Bar = 10 μm. [file 12862_2021_1750_MOESM4_ESM.tif]

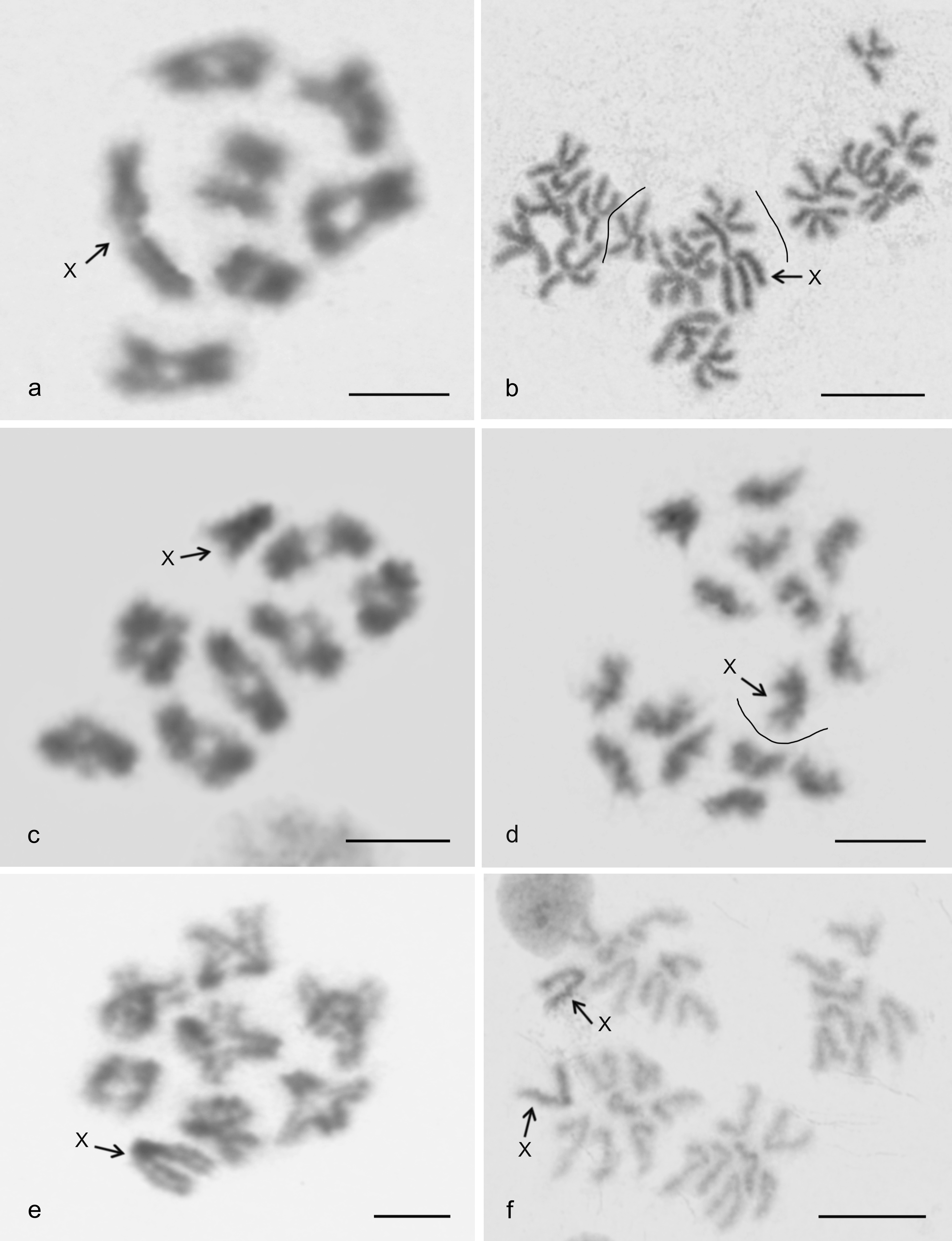

Supplement: Supplementary file 5 — Additional file 5: Fig. S3. Sex chromosomes of artemines with the X0 system. Stained by Giemsa. X = X chromosome. (a, b) Chisosa diluta. a Metaphase I, consisting of six bivalents and a peripheral X chromosome. b Group of metaphases II separated by lines. It consists of one metaphase containing a positively heteropycnotic X chromosome (n = 7, in the middle of the plate) and two metaphases without sex chromosome (left metaphase is incomplete); (c, d) Holocneminus sp. c Metaphase I, comprising seven bivalents and peripheral X chromosome. d Two sister metaphases II separated by a line (n = 8 including X chromosome + n = 7); (e, f) Physocyclus dugesi. e Diplotene, comprising seven bivalents and peripheral X chromosome. f Anaphase II. Note slight positive heteropycnosis of X chromosome. Bar = 10 μm. [file 12862_2021_1750_MOESM5_ESM.tif]

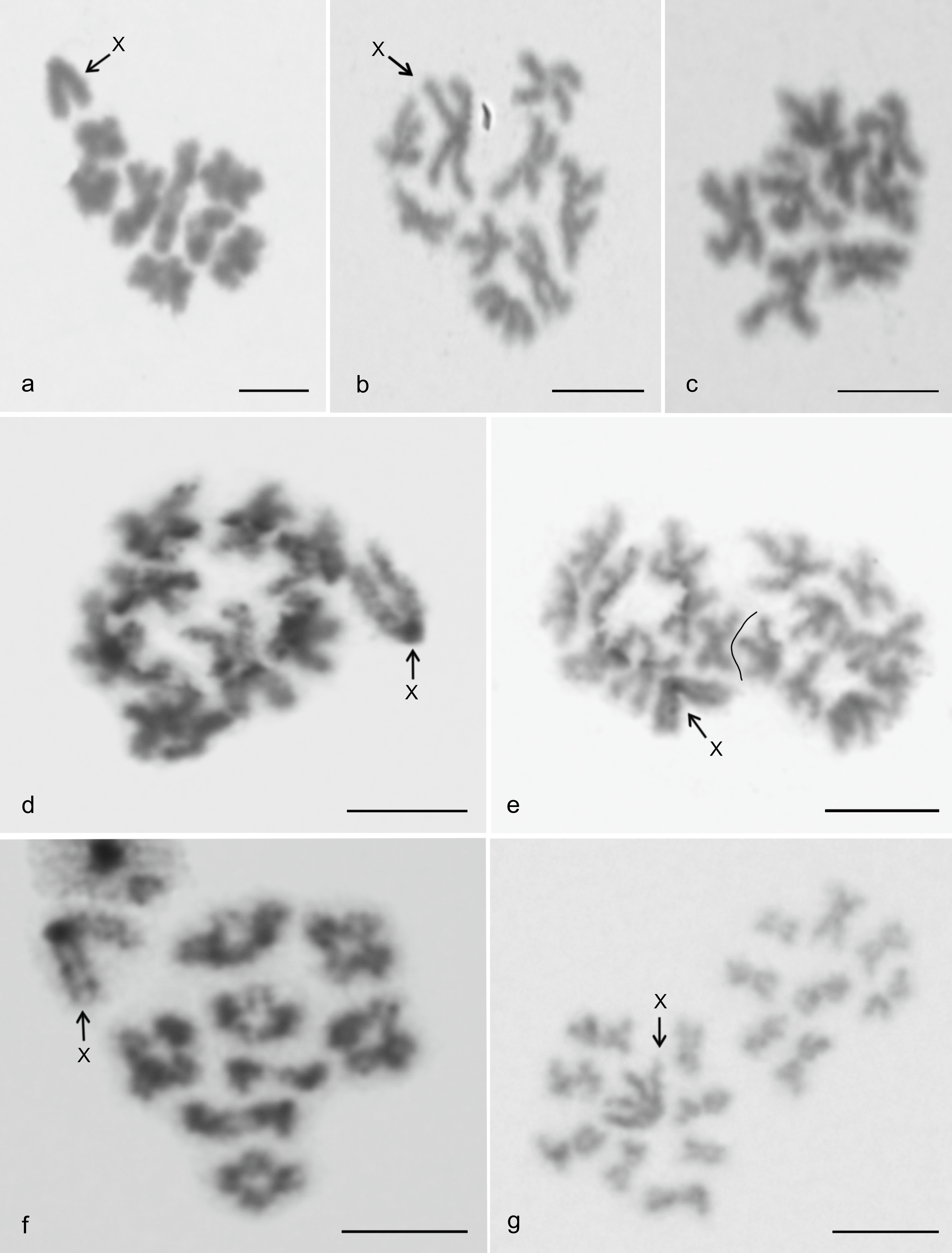

Supplement: Supplementary file 8 — Additional file 8: Fig. S5. Sex chromosome systems of modisimines. Stained by Giemsa. X = X chromosome. (a–c) Anopsicus sp. (X0) a metaphase I, consisting of eight bivalents and peripheral X chromosome. b Metaphase II, including X chromosome (n = 9). c Metaphase II, without X chromosome (n = 8); (d, e) Modisimus cf. elongatus (X0). d Diplotene, comprising eight bivalents and a peripheral X chromosome. e Two sister metaphases II separated by a line (n = 8 + n = 9, including peripheral X chromosome); (f, g) Psilochorus pallidulus (X0). f Diplotene, comprising eight bivalents and peripheral X chromosome. g Two sister metaphases II (n = 9, including X chromosome + n = 8). Bar = 10 μm. [file 12862_2021_1750_MOESM8_ESM.tif]

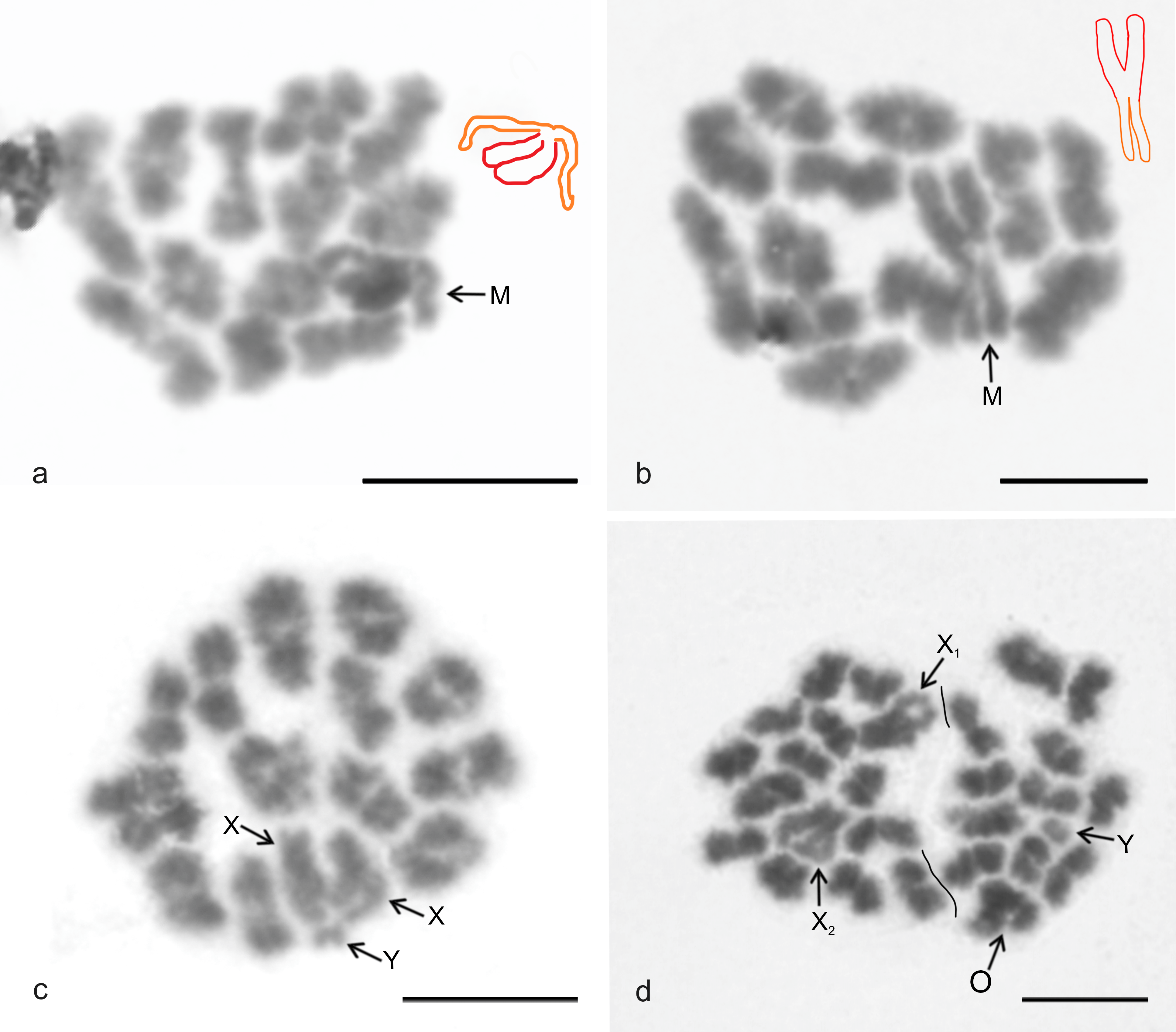

Supplement: Supplementary file 10 — Additional file10: Fig. S7. Sex chromosome systems of ninetines. Stained by Giemsa. Figures a and b contain a scheme of the multivalent. M = sex chromosome multivalent, O = overlapping of two bivalents, X = X chromosome, X1 = X1 chromosome, X2 = X2 chromosome, Y = Y chromosome. (a, b) Kambiwa neotropica (X1X2X3X4Y), plates of the first meiotic division consisting of 12 bivalents plus a sex chromosome multivalent consisting of four “arms”. Two “arms” are thick (red) and two are thin (orange). a Diakinesis. b Metaphase I. Note cross-shaped morphology of multivalent; (c, d) Pholcophora americana (X1X2Y). c Diakinesis, comprising 13 bivalents and sex chromosomes X1, X2, and Y. Sex chromosomes show end-to-end paring. d Two sister metaphases II separated by a line (n = 15, including chromosomes X1 and X2 + n = 14 including Y microchromosome). Bar = 10 μm. [file 12862_2021_1750_MOESM10_ESM.tif]

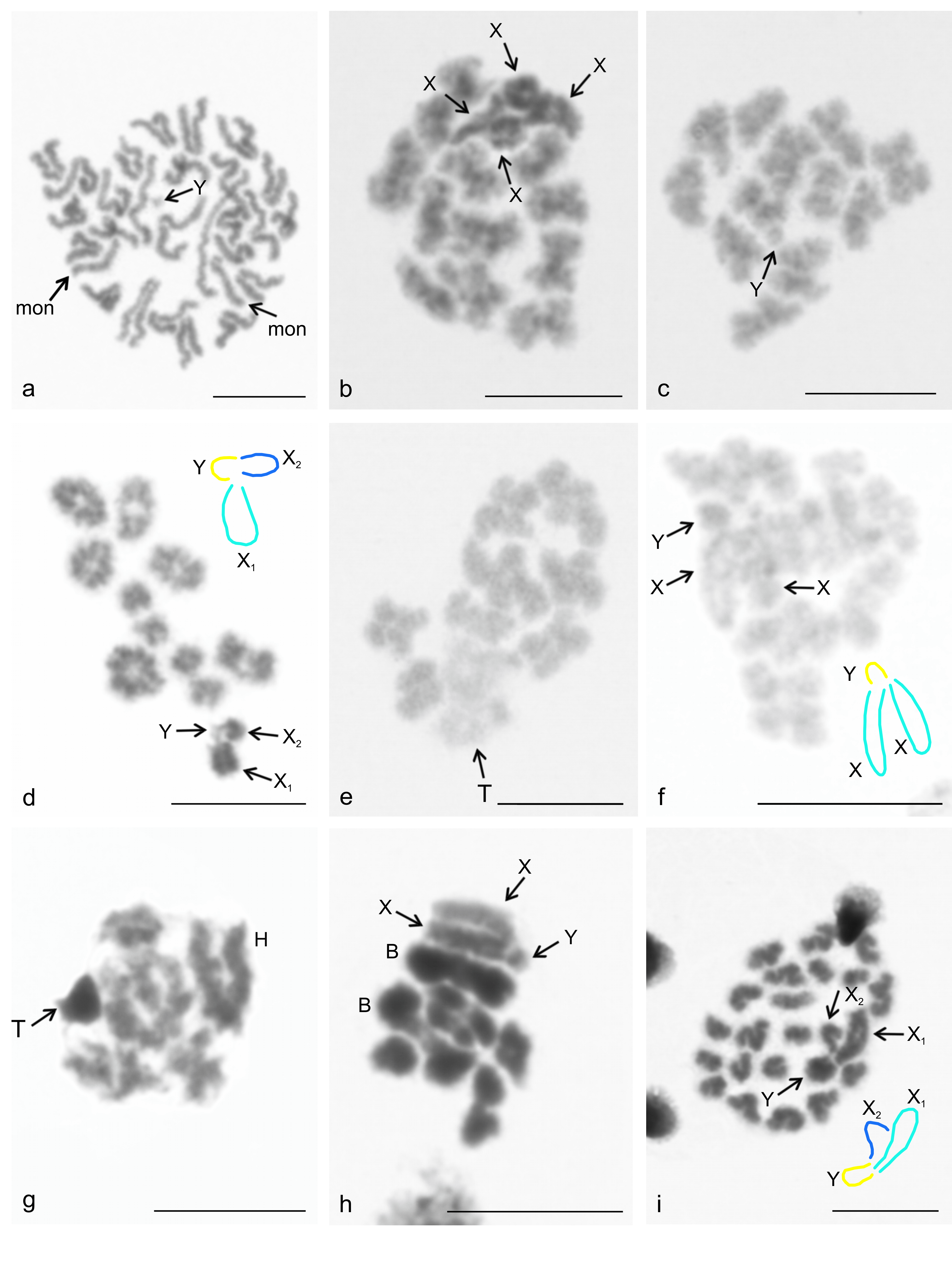

Supplement: Supplementary file 11 — Additional file 11: Fig. S8. Cytogenetics of ninetines (a–c) and pholcines (d–i), male germline. Figures d, f, i contain scheme of sex chromosome trivalent X1X2Y. B = large bivalent, H = large bivalent exhibiting positive heteropycnosis, mon = monoarmed X chromosome, T = sex chromosome trivalent, X = X chromosome, X1 = X1 chromosome, X2 = X2 chromosome, Y = Y chromosome. (a–c) Kambiwa neotropica (X1X2X3X4Y). a Spermatogonial metaphase (2n = 29). Chromosomes are biarmed, except for two monoarmed chromosomes. b Metaphase II, consisting of 12 chromosomes and cluster of four positively heteropycnotic X chromosomes. c Metaphase II, formed by 12 chromosomes and a Y microchromosome; d Aetana kinabalu (X1X2Y), incomplete metaphase I. Sex chromosomes pair by ends of their arms, X chromosomes are positively heteropycnotic; (e, f) Metagonia sp. (X1X2Y), late prophase I. Note low chromosome condensation. e Diplotene. Plate consists of eight bivalents and almost decondensed sex chromosomes. f Diakinesis, note the X1X2Y trivalent, Y chromosome more condensed than X chromosomes; (g, h) Pehrforsskalia conopyga (X1X2Y). g Early diplotene. Note sex chromosomes forming a compact positively heteropycnotic body and large positively heteropycnotic bivalent. h Metaphase I, formed by seven bivalents and sex chromosome trivalent. Two bivalents (B) are much longer than the remaining ones. Note tiny Y chromosome; i Pholcus bamboutos (X1X2Y), transition from metaphase to anaphase I. Note the delayed separation of sex chromosomes. Only one end of the X2 chromosome takes part in pairing. The Y chromosome is positively heteropycnotic. Bar = 10 μm. [file 12862_2021_1750_MOESM11_ESM.tif]

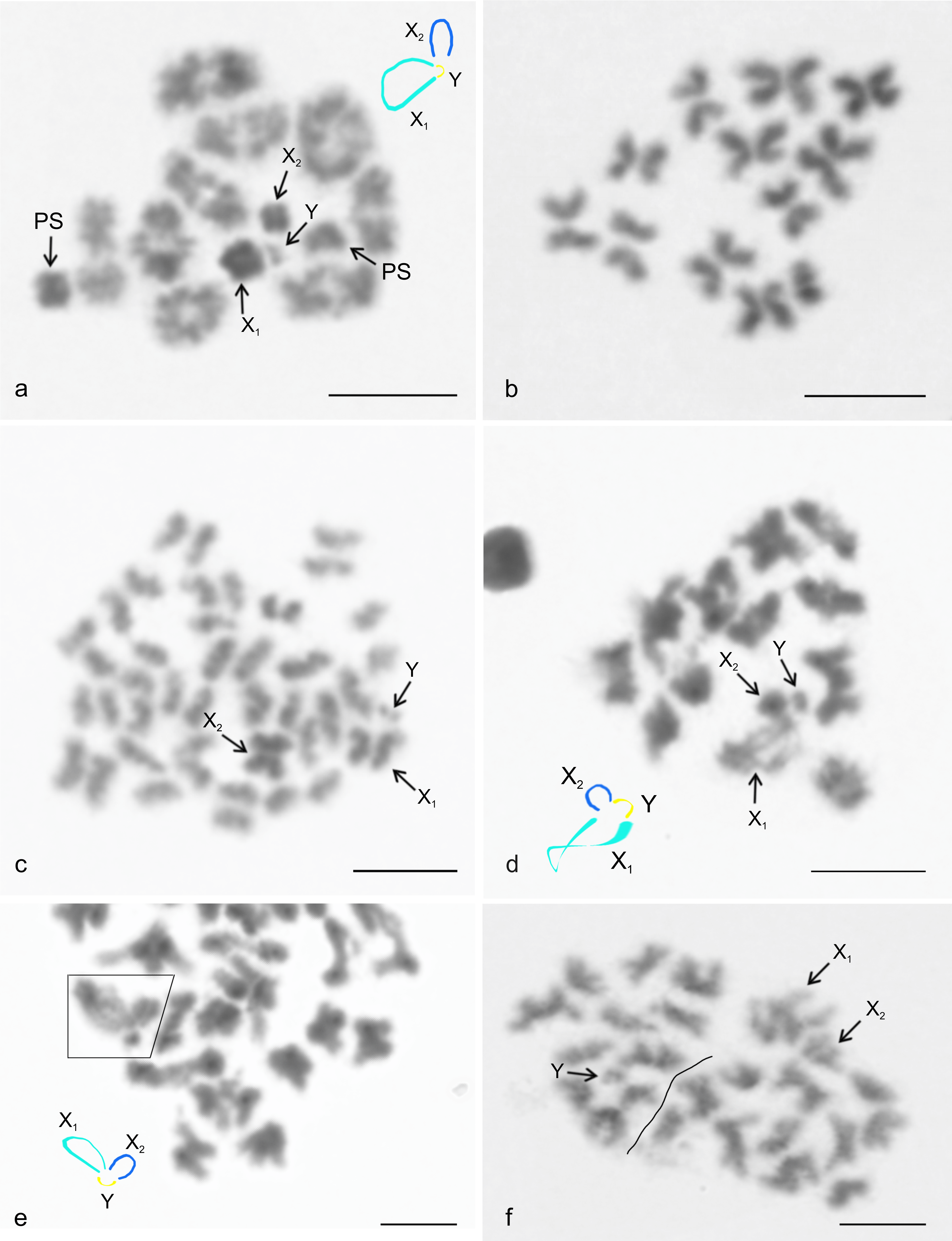

Supplement: Supplementary file 13 — Additional file 13: Fig. S9. Sex chromosomes of pholcines with the X1X2Y system, part I. Stained by Giemsa. Figures a, d, e contain a scheme of the sex chromosome trivalent. X1 = X1 chromosome, X2 = X2 chromosome, Y = Y chromosome, PS = precocious separation of chromosomes of the bivalent. (a–c) Aetana kinabalu. a Metaphase I, comprising 11 bivalents (one bivalent shows a precocious separation of chromosomes) and a sex chromosome trivalent. X chromosomes are positively heteropycnotic. b Metaphase II, containing X chromosomes (n = 12). c Transition metaphase II/anaphase II, fusion of two sister plates. Note X chromosomes exhibiting a delayed separation of chromatids and a Y microchromosome; (d–f) Nipisa deelemanae. d Metaphase I, comprising 11 bivalents and sex chromosome trivalent. e Part of a plate formed by several fused metaphases I, sex chromosome trivalent encircled. f Two sister metaphases II separated by a line (n = 12, including Y microchromosome + n = 13 including metacentric chromosomes X1 and X2). Note the reduction of the X2 chromosome. Bar = 10 μm. [file 12862_2021_1750_MOESM13_ESM.tif]

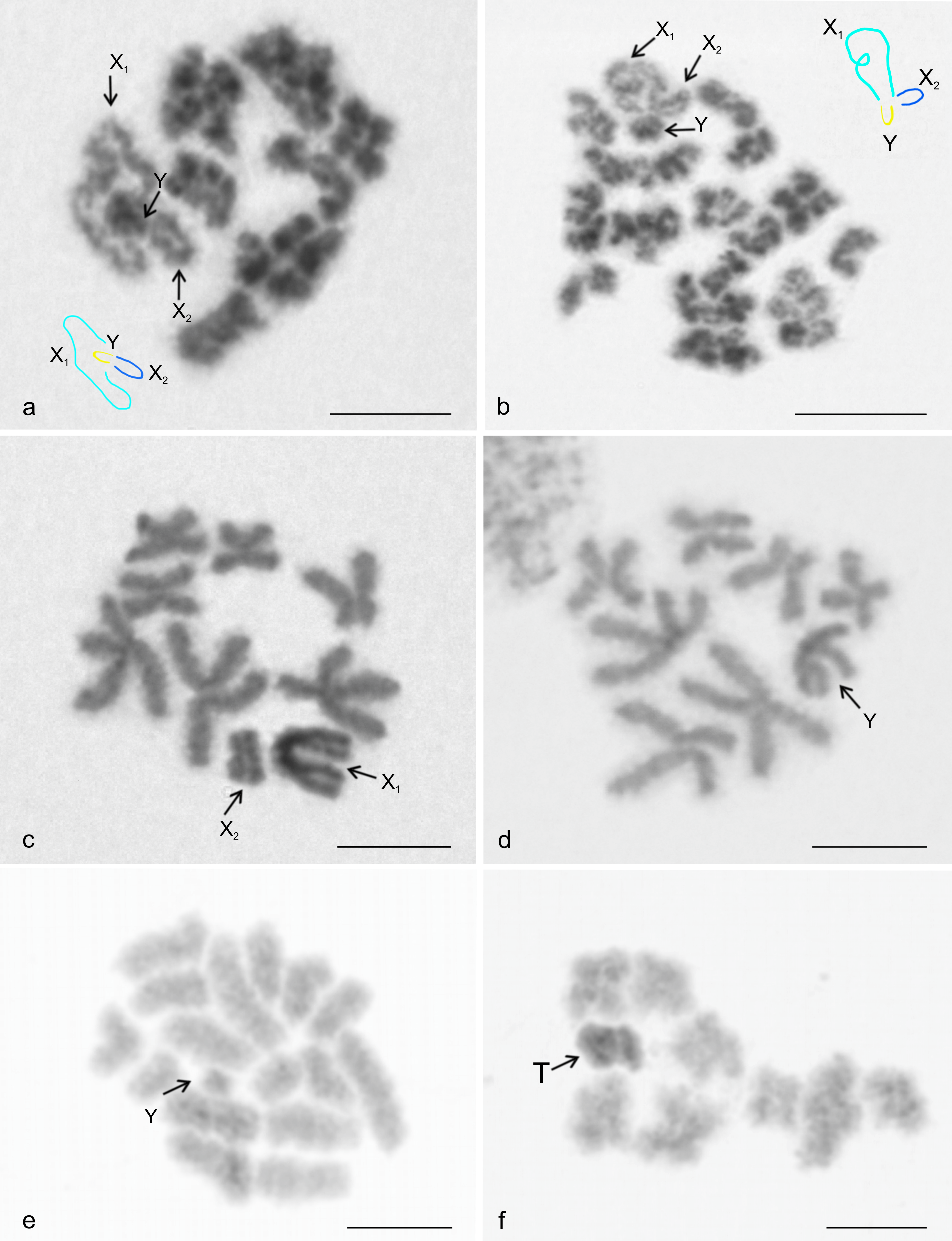

Supplement: Supplementary file 14 — Additional file14: Fig. S10. Sex chromosomes of pholcines with the X1X2Y system, part II. Stained by Giemsa. Figures a, b contain a scheme of the sex chromosome trivalent. T = trivalent, X1 = X1 chromosome, X2 = X2 chromosome, Y = Y chromosome. (a–d) Leptopholcus guineensis. a Early diplotene, consisting of seven bivalents and a sex chromosome trivalent, X chromosomes exhibit a low condensation. b Two fused diplotene. c Metaphase II, including X chromosomes (n = 9). X1 chromosome is positively heteropycnotic. d Metaphase II, including a Y chromosome (n = 8); (e, f) Metagonia sp. e Spermatogonial metaphase, note the Y microchromosome. f Diplotene, note the positively heteropycnotic body formed by the sex chromosomes. Bar = 10 μm. [file 12862_2021_1750_MOESM14_ESM.tif]

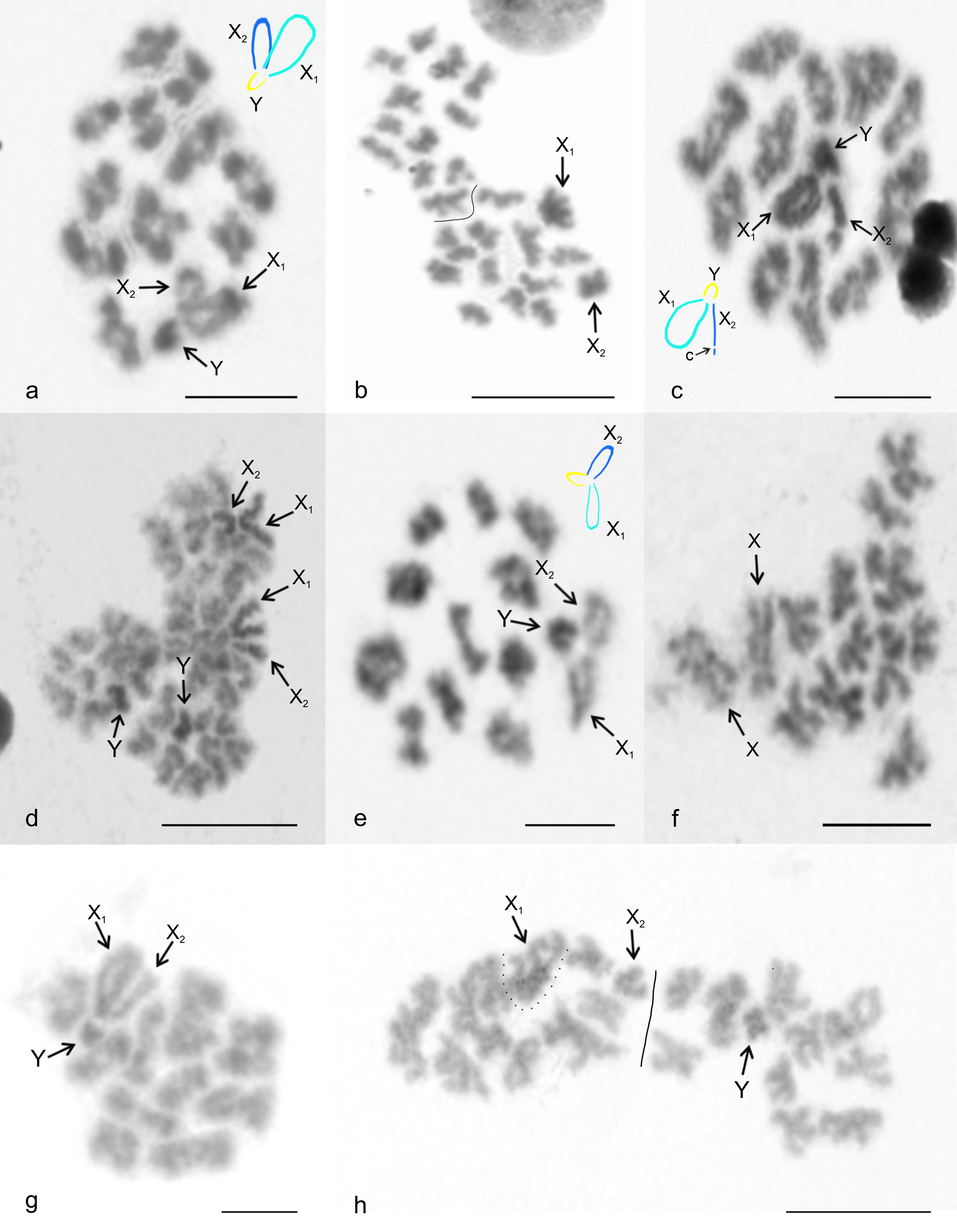

Supplement: Supplementary file 15 — Additional file15: Fig. S11. Sex chromosomes of pholcines with the X1X2Y system, part III. Stained by Giemsa. Figures a, c, e contain a scheme of the sex chromosome trivalent. c = centromere, X1 = X1 chromosome, X2 = X2 chromosome, Y = Y chromosome. (a, b) Muruta tambunan. a Diakinesis, consisting of 11 bivalents and a sex chromosome trivalent. b Two sister metaphases II separated by a line (n = 12, including Y chromosome + n = 13 including chromosomes X1 and X2). Note the metacentric X1 chromosome and submetacentric X2 chromosome on the periphery of the plate. They exhibit positive heteropycnosis; (c, d) Pholcus phalangioides. c Diakinesis, comprising 11 bivalents and a sex chromosome trivalent, which is placed in the middle of the plate and exhibits positive heteropycnosis. Concerning the X2 chromosome, only end of the long arm is involved in pairing. d Anaphase II. Note the positive heteropycnosis of the sex chromosomes. The X chromosomes are associated; (e, f) Spermophora senoculata. e Metaphase I, comprising 11 bivalents and a sex chromosome trivalent. f Metaphase II, X chromosomes are less condensed than the other chromosomes; (g, h) Quamtana hectori. g Metaphase I, composed of 10 bivalents and a sex chromosome trivalent. Concerning the X2 chromosome, only one end is involved in pairing. h Two sister metaphases II separated by a line (n = 11 including Y chromosome + n = 12 including chromosomes X1 and X2). Bar = 10 μm. [file 12862_2021_1750_MOESM15_ESM.tif]

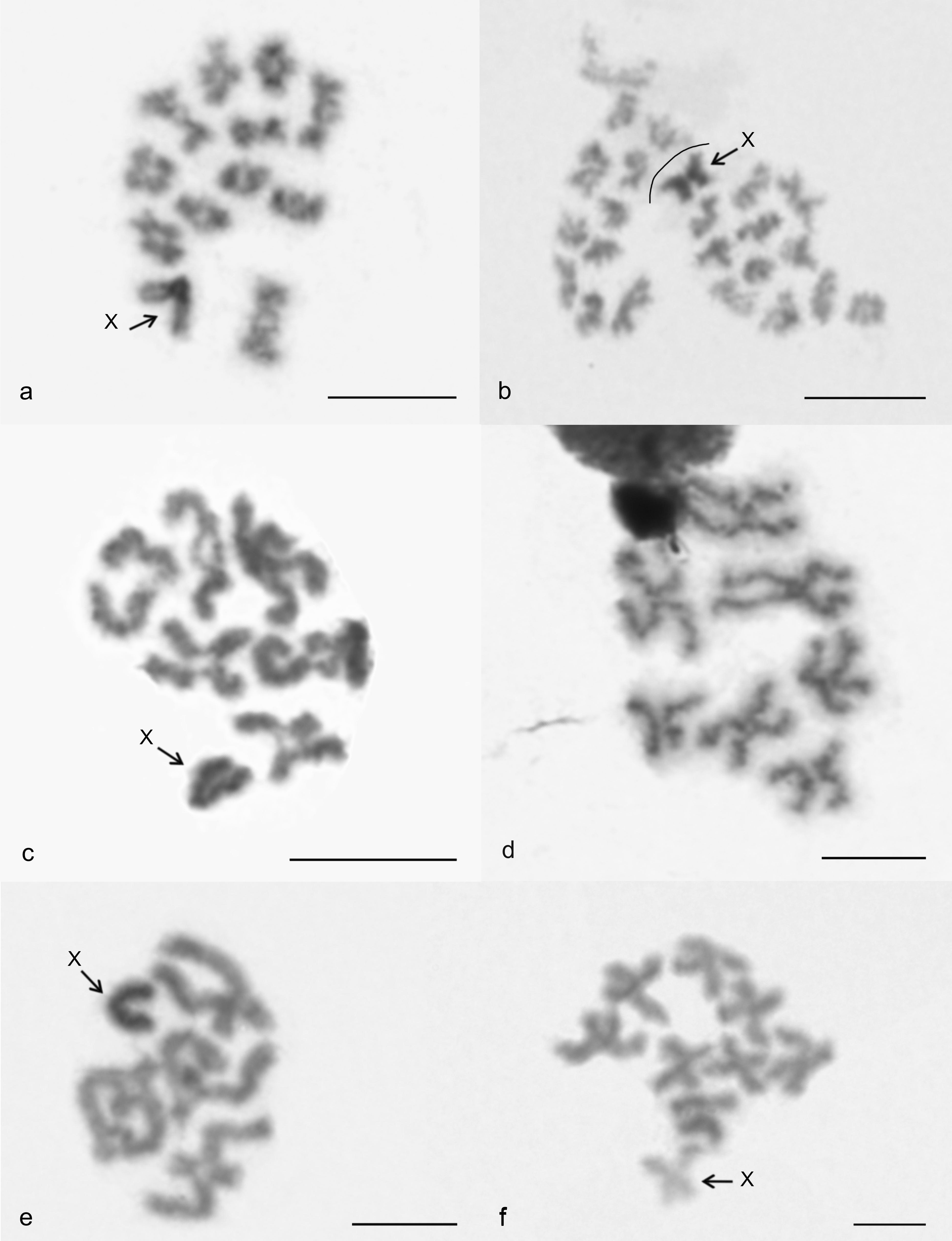

Supplement: Supplementary file 17 — Additional file 17: Fig. S13. Sex chromosomes of pholcines with the X0 system. Stained by Giemsa. X = X chromosome. (a, b) Belisana sabah. a Metaphase I, consisting of 11 bivalents and a peripheral X chromosome. b Two sister metaphases II separated by a line (n = 11 + n = 12, including a positively heteropycnotic X chromosome); (c, d) Cantikus sabah. c Diplotene, comprising six bivalents and a positively heteropycnotic X chromosome placed on the periphery of the plate. d Prometaphase II including the X chromosome (n = 7); (e, f) Micropholcus fauroti. e Diplotene composed of four bivalents and a positively heteropycnotic X chromosome placed on the periphery of the plate. f Plate formed by fusion of two sister metaphases II. It includes a negatively heteropycnotic X chromosome. Bar = 10 μm. [file 12862_2021_1750_MOESM17_ESM.tif]

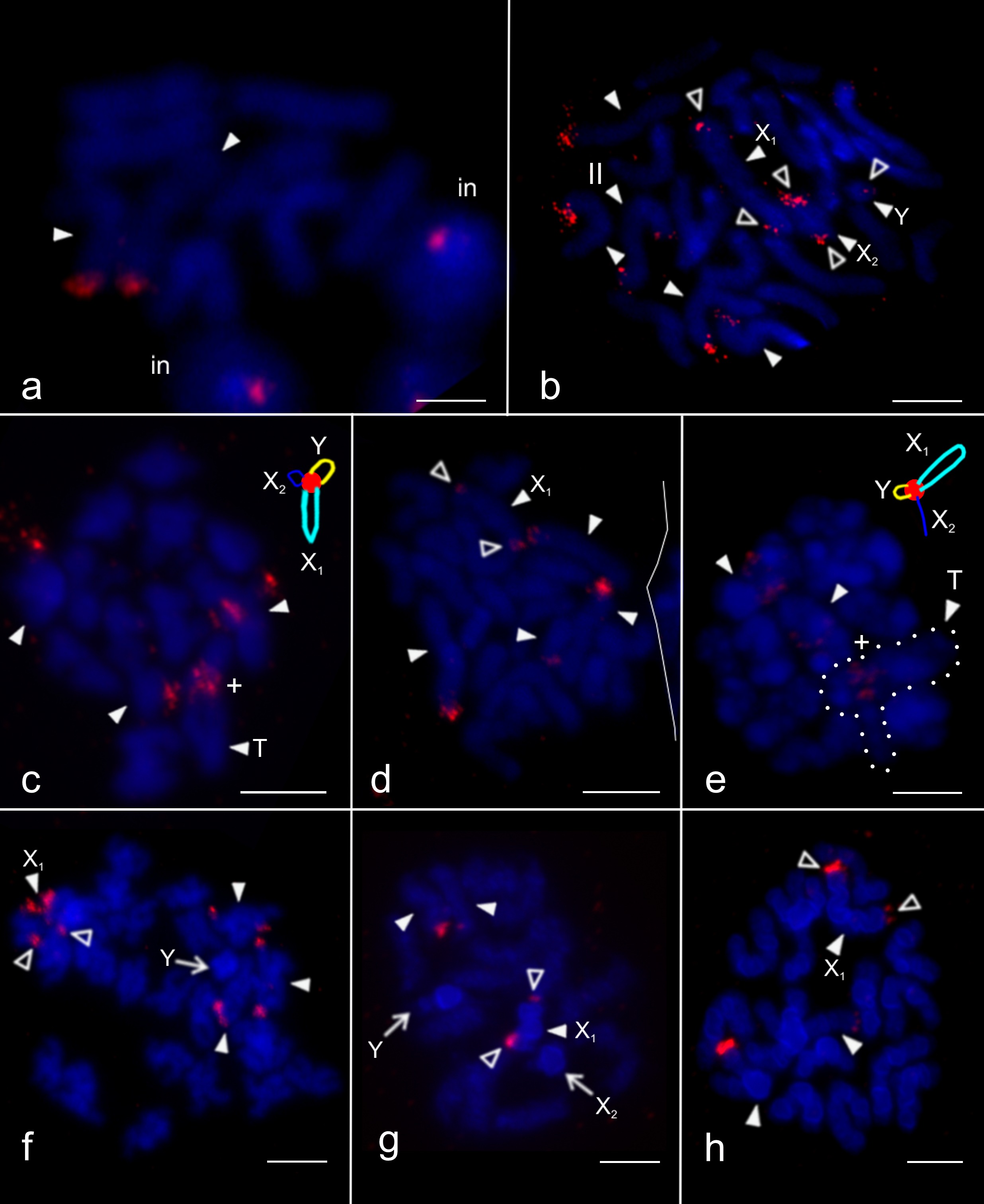

Supplement: Supplementary file 18 — Additional file 18: Fig. S14. Pholcinae, males, detection of NORs (FISH). Figures c, e contain a scheme of the sex chromosome trivalent X1X2Y (red = signal formed by several NORs). Arrowhead = NOR-bearing chromosome (a, b, d, f–h), bivalent (c, e) or trivalent (c, e), open arrowhead = sex chromosome-linked NOR, T = sex chromosome trivalent, X1 = X1 chromosome, X2 = X2 chromosome, Y = Y chromosome, + = signal formed by several NORs. a Micropholcus fauroti (X0), mitotic metaphase. Note association of two homologous chromosomes containing terminal NOR (in – interphase nucleus); (b, c) Nipisa deelemanae (X1X2Y). b Mitotic metaphase. X chromosomes (X1, X2) and another chromosome (II) bear a terminal NOR at both ends. Five other chromosomes, including Y chromosome, involves one terminal NOR only. The sex chromosomes X1 and X2 are associated in parallel in the middle of the plate. b Metaphase I, note the three bivalents bearing a NOR and the sex chromosome trivalent with a signal in the region of chromosome pairing (see scheme); (d–f) Quamtana hectori (X1X2Y). d Mitotic metaphase (separated by a line from another plate). X1 chromosome bears two NORs, each at opposite end of the chromosome. Chromosomes of two pairs also include a terminal NOR; e Metaphase I, two bivalents contain NOR. The sex chromosome trivalent contains a signal in region of chromosome pairing (see scheme). f Plate formed by fused sister metaphases II, chromosomes of NOR-bearing pairs exhibit biarmed morphology. The X1 chromosome is terminated by NOR at both ends. Y chromosome considerably condensed, without signal; (g, h) Q. filmeri (X1X2Y), mitotic plates. The X1 chromosome bears two NORs, each at opposite end of chromosome. Chromosomes of one pair also contain a terminal NOR. g Prophase, sex chromosomes exhibit a more intensive fluorescence than the other chromosomes. h Metaphase. Bar = 5 μm except for c, e, g (10 μm). [file 12862_2021_1750_MOESM18_ESM.tif]

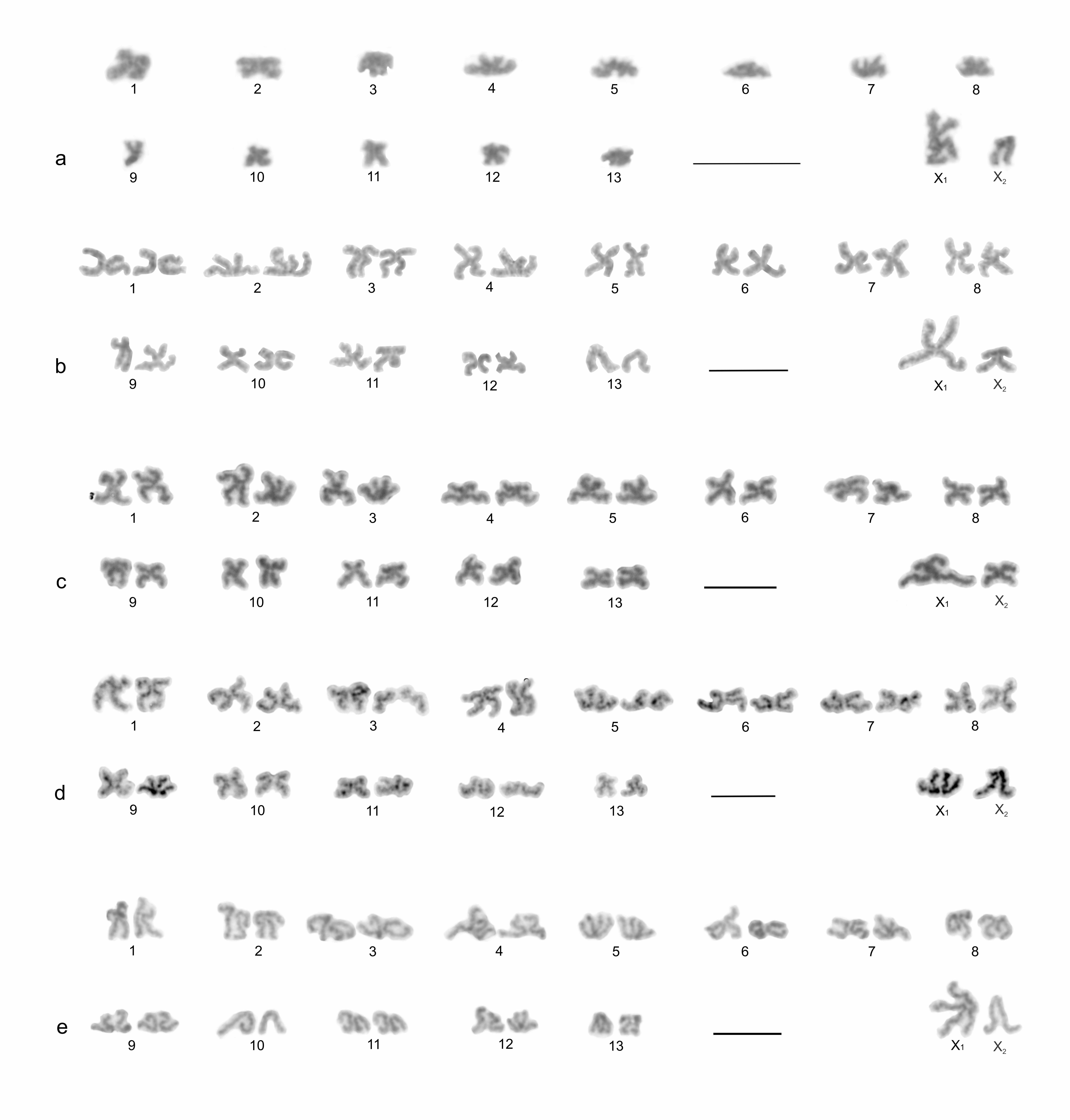

Supplement: Supplementary file 20 — Additional file 20: Fig. S15. Hoplopholcus and Smeringopus (Smeringopinae), male karyotypes, stained by Giemsa. Based on metaphase II (a) or two sister metaphases II (b–e). Autosome pairs decrease gradually in size. The X1 is the longest element of the set (except for d). Karyotypes are predominated by metacentrics. a H. labyrinthi, haploid set, note the two submetacentric chromosomes (nos 5, 9), subtelocentric chromosome (no. 6) and subtelocentric X2 chromosome; b S. atomarius, note one submetacentric (no. 3) and one acrocentric pairs (no. 13), and submetacentric X2; c S. ndumo, note two submetacentric pairs (nos 4, 11); d S. peregrinus, note three submetacentric (nos 1, 4, 6) and one subtelocentric pairs (no. 12), and subtelocentric X2. Sex chromosomes positively heteropycnotic; e Smeringopus sp., note two submetacentric pairs (nos 1, 2), one acrocentric pair (no. 10) and acrocentric X2. Bar = 10 μm. [file 12862_2021_1750_MOESM20_ESM.tif]

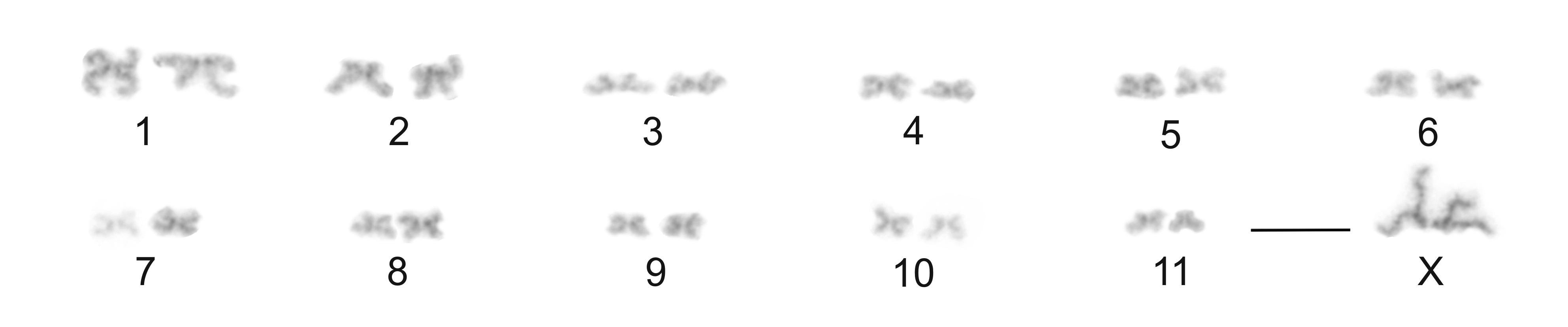

Supplement: Supplementary file 21 — Additional file 21: Fig. S16. Crossopriza lyoni (Smeringopinae), male karyotype, Giemsa staining. Based on two sister metaphases II. Karyotype metacentric, except for submetacentric pairs nos 2 and 11. First two pairs differ from the other ones by large size. The X chromosome is the longest chromosome of the set. It is slightly positively heteropycnotic. Bar = 10 μm. [file 12862_2021_1750_MOESM21_ESM.tif]

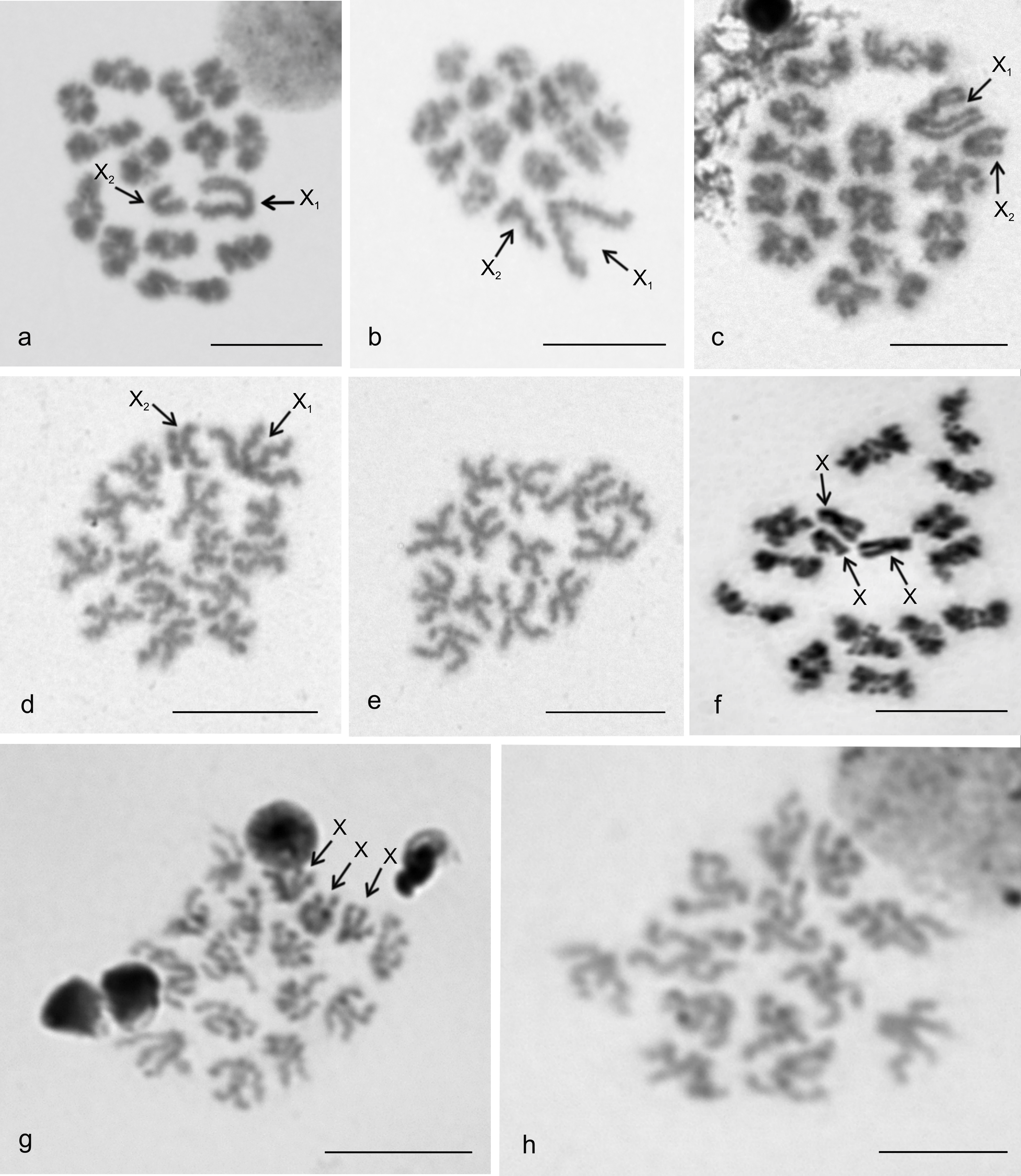

Supplement: Supplementary file 22 — Additional file 22: Fig. S17. Sex chromosomes of smeringopines with multiple X chromosomes. Stained by Giemsa. X = X chromosome, X1 = X1 chromosome, X2 = X2 chromosome. (a, b) Hoplopholcus forskali (X1X20). a Diakinesis, composed of 13 bivalents and two X chromosomes, note the end-to-end association of the X chromosomes. b Telophase I, half plate containing X chromosomes; (c–e) Smeringopus ndumo (X1X20). c Diakinesis, comprising 13 bivalents and two X chromosomes. d Metaphase II, containing X chromosomes (n = 15). e Metaphase II, without the sex chromosomes (n = 13); (f–h) S. pallidus (X1X2X30). f Diakinesis, composed of 13 bivalents and three X chromosomes, sex chromosomes grouped in the middle of the plate. g Metaphase II with X chromosomes (n = 16). X chromosomes are associated at the periphery of the plate. They exhibit a slight positive heteropycnosis. h Metaphase II, without sex chromosomes (n = 13). Bar = 10 μm. [file 12862_2021_1750_MOESM22_ESM.tif]

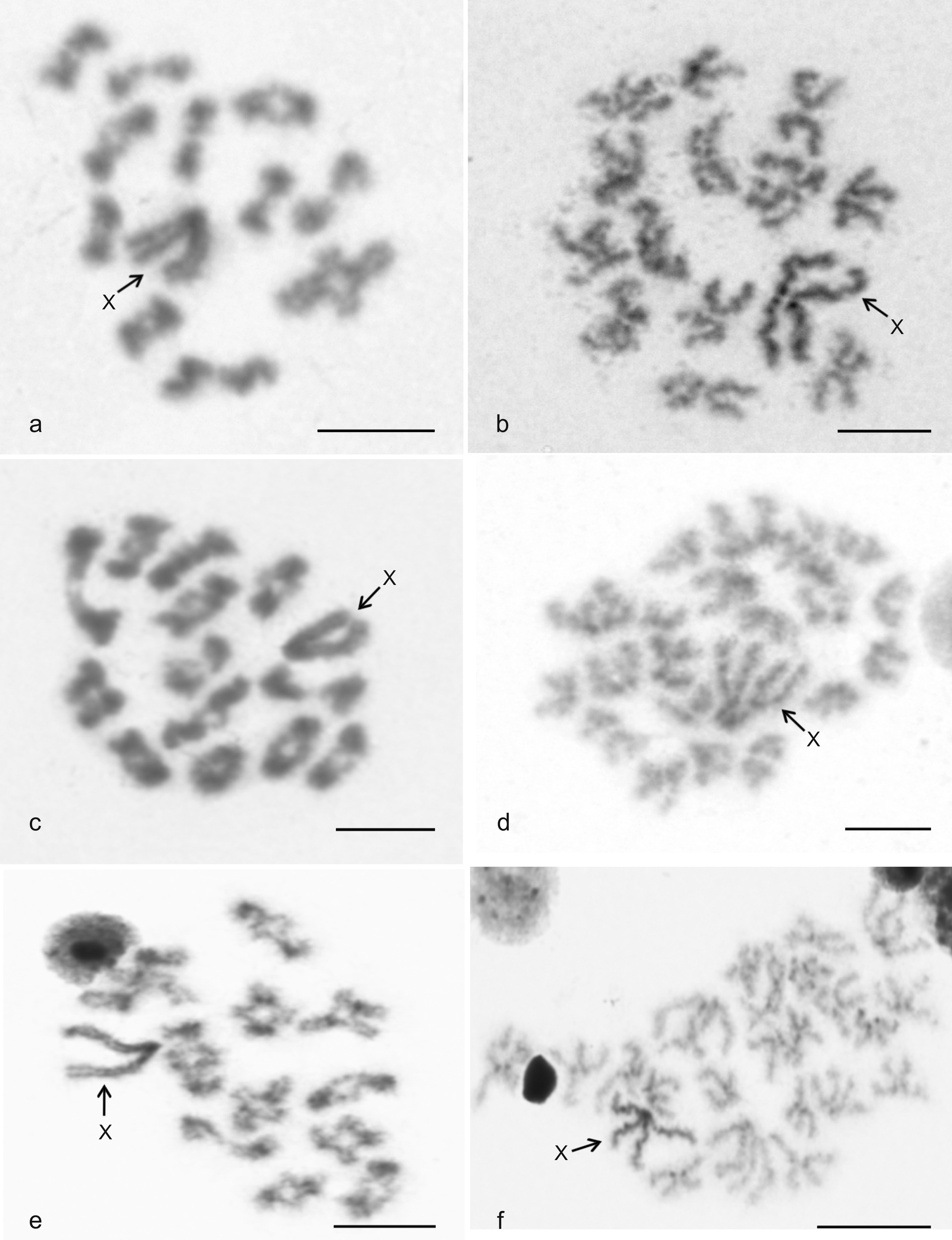

Supplement: Supplementary file 23 — Additional file 23: Fig. S18. Sex chromosomes of smeringopines with the X0 system. Stained by Giemsa. X = X chromosome. (a, b) Crossopriza lyoni. a Metaphase I composed of 11 bivalents and X chromosome. b Anaphase I; (c, d) Holocnemus pluchei. c Metaphase I, consisting of 13 bivalents and an X chromosome. The X chromosome is placed at the periphery of the plate. Note the association of terminal parts of the X chromosome arms. d Metaphase II, including the X chromosome. This element is slightly positively heteropycnotic; (e, f) Stygopholcus skotophilus. e Diplotene, composed of 11 bivalents and an X chromosome. f Plate formed by two fused sister metaphases II, 2n = 23. Note the positively heteropycnotic X chromosome. Bar = 10 μm. [file 12862_2021_1750_MOESM23_ESM.tif]

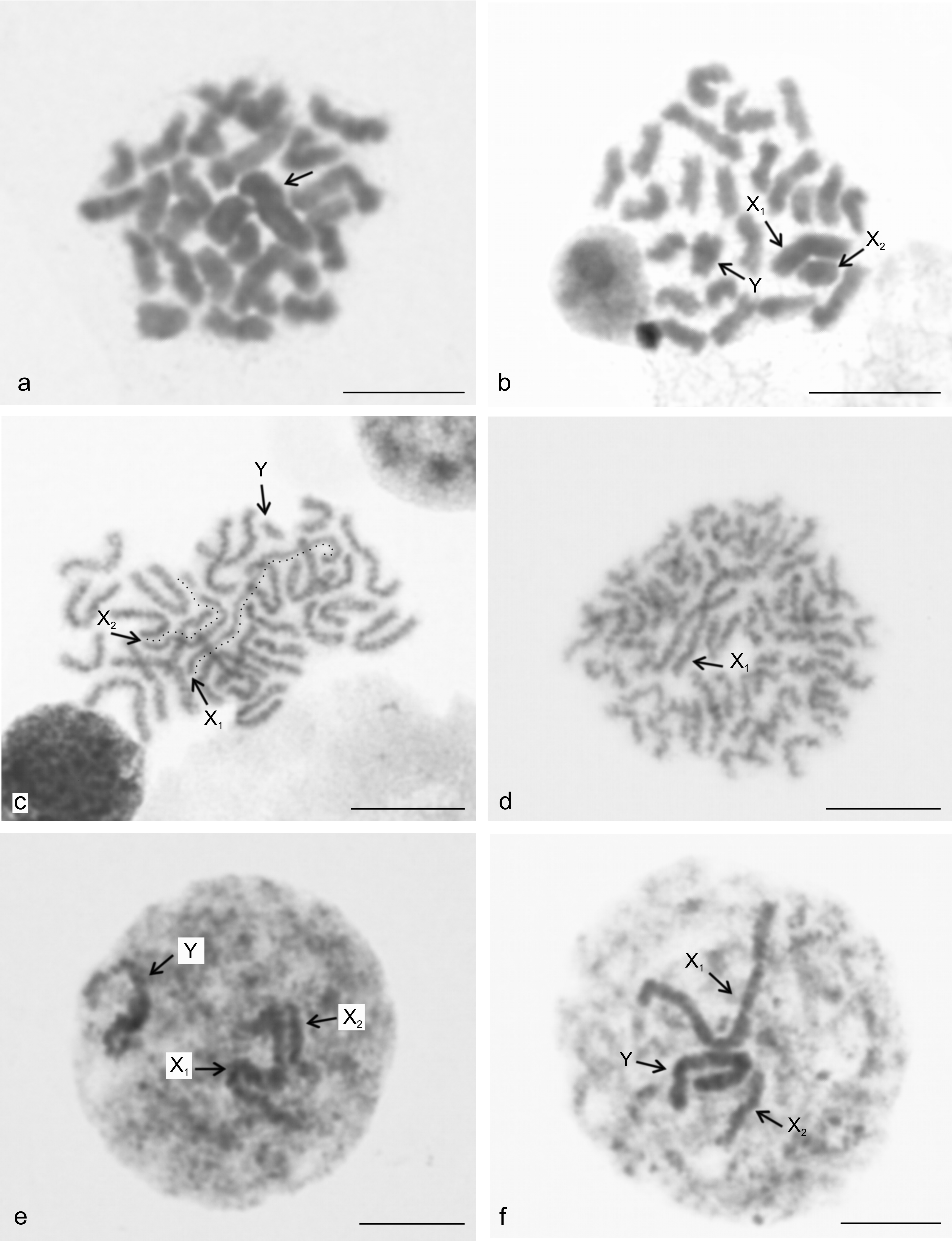

Supplement: Supplementary file 24 — Additional file 24: Fig. S19. Pholcidae, male germline, behaviour of sex chromosomes prior to meiosis. Arrow = sex chromosomes, X1 = X1 chromosome, X2 = X2 chromosome, Y = Y chromosome. (a, b) Muruta tambunan (X1X2Y), mitotic metaphase, chromosomes X1, X2, and Y positively heteropycnotic. Chromosomes X2 and Y are approximately of the same size. a Sex chromosomes grouped in the middle of the plate. b Chromosomes X1 and X2 associated in parallel, Y chromosome released from the association; c Artema nephilit (X1X2Y), early mitotic metaphase, X chromosomes are marked by a dotted line. They are associated in parallel in the middle of the plate. Their condensation is slightly delayed in comparison with the other chromosomes; d Hoplopholcus cecconii (X1X20), transition from mitotic metaphase to anaphase. Chromosome X1 is placed in the middle of the plate; (e, f) Pholcus kindia (X1X2Y). e Premeiotic interphase. X chromosomes pair in parallel in the middle of the nucleus. The Y chromosome does not take part in pairing. f Preleptotene. Sex chromosomes are associated in the middle of the nucleus, the Y chromosome is more condensed than the X chromosomes. Bar = 10 μm. [file 12862_2021_1750_MOESM24_ESM.tif]

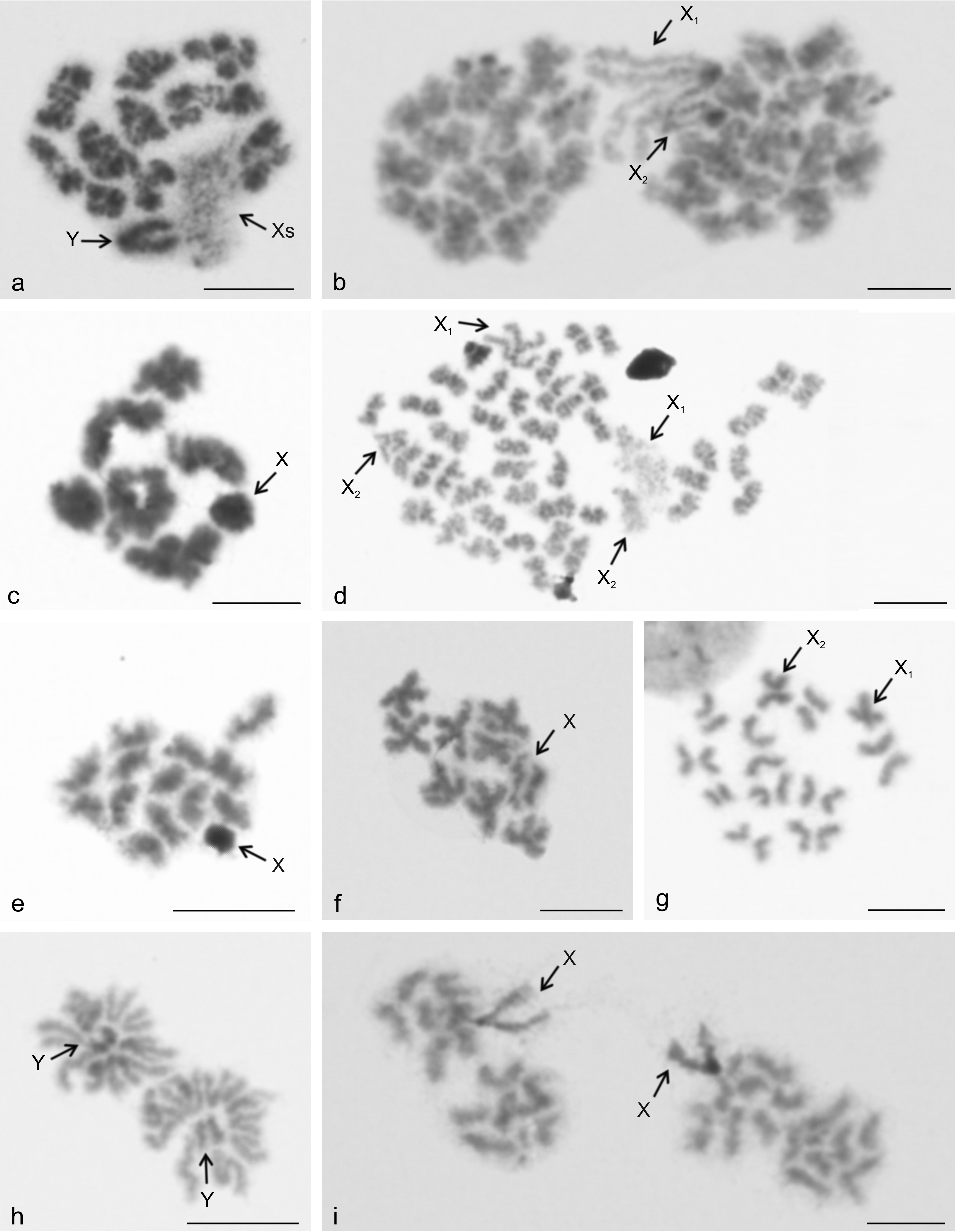

Supplement: Supplementary file 25 — Additional file 25: Fig. S20. Pholcidae, male meiosis, condensation and segregation of sex chromosomes. X = X chromosome, Xs = X chromosomes, X1 = X1 chromosome, X2 = X2 chromosome, Y = Y chromosome. a Pholcus kindia (X1X2Y), early diplotene. Y chromosome highly condensed. In contrast, X chromosomes almost decondensed; b Hoplopholcus cecconii (X1X20), late anaphase I. X chromosomes are arranged in parallel and less condensed than the other chromosomes. Moreover, their segregation and separation of their chromatids are delayed. Centromeres of sex chromosomes are formed by a prominent knob; c Cantikus sabah (X0), diplotene. Sex chromosome forms a highly condensed body; d Hoplopholcus forskali (X1X20), plate formed by fusion of 1) two sister late prometaphases II (left) and 2) two sister early prometaphases II (right). In contrast to autosomes, sex chromosomes differ considerably by condensation in early and late prometaphase II. They are almost decondensed during early prometaphase II (right); e Holocneminus sp. (X0), plate formed by two sister prometaphases II, sex chromosome forms a highly condensed body; f Micropholcus fauroti (X0), two fused sister metaphases II. Sex chromosome shows precocious division; g Aetana kinabalu (X1X2Y), late metaphase II, division of X chromosomes is delayed; h Pholcus sp. (X1X2Y), two half-plates of anaphase II containing positively heteropycnotic Y chromosome in the middle; i Psilochorus simoni (X0), two sister anaphases II. Segregation of X chromosome delayed. This element is slightly positively heteropycnotic at right anaphase II. Bar = 10 μm. [file 12862_2021_1750_MOESM25_ESM.tif]

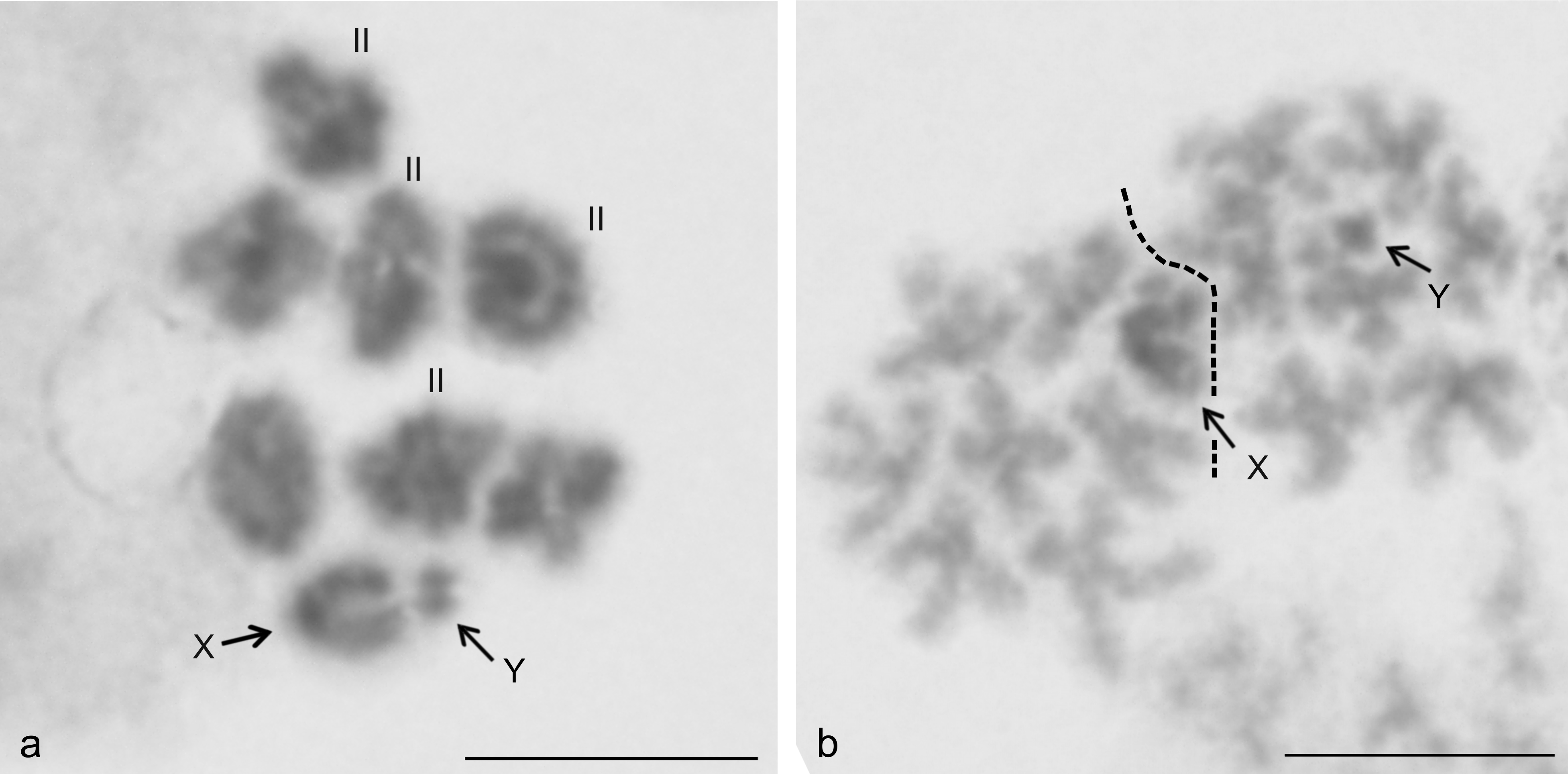

Supplement: Supplementary file 26 — Additional file 26: Fig. S21. Arteminae, Wugigarra sp., male meiosis, behaviour of sex chromosomes. X = X chromosome, Y = Y chromosome, II = bivalent containing two chiasmata. a Metaphase I, three bivalents include two chiasmata. Pairing of metacentric chromosomes X and Y is ensured by ends of their arms. b Two sister metaphases II (separated by dashed line). While X chromosome is placed at the periphery of one plate, Y chromosome is in middle of another plate. Note positive heteropycnosis of sex chromosomes. Bar = 10 μm. [file 12862_2021_1750_MOESM26_ESM.tif]

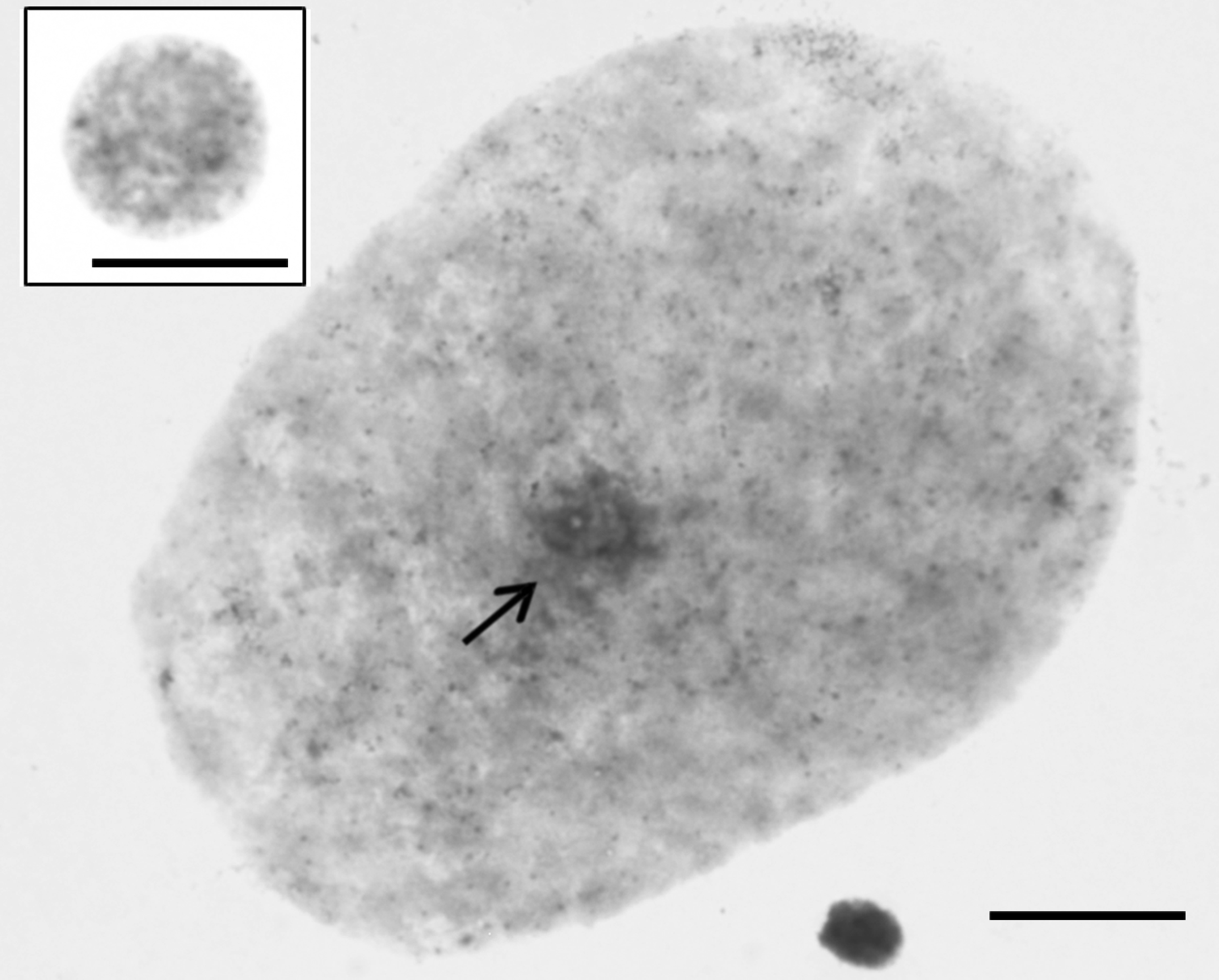

Supplement: Supplementary file 27 — Additional file 27: Fig. S22. Pholcus sp. (X1X2Y), testes, endopolyploid nucleus. Heterochromatic body in the middle of the nucleus is formed by sex chromosomes (arrow). Inset: a standard diploid nucleus. Bar = 5 μm. [file 12862_2021_1750_MOESM27_ESM.tif]
