# Supplementary material for: Evolutionary pattern of karyotypes and meiosis in pholcid spiders (Araneae: Pholcidae): implications for reconstructing chromosome evolution of araneomorph spiders
Source: BMC Ecol Evol. 2021 May 3;21:75. doi: 10.1186/s12862-021-01750-8 (PMC8091558; doi:10.1186/s12862-021-01750-8)
Supplement: Supplementary file 7 — Additional file 7: Table S3. Modisiminae and Ninetinae, summary of male cytogenetic data, including results of other authors. Doubtful data are not included. See database [24] for full list of published data on pholcid karyotypes, including doubtful data. Abbreviations: bi = biarmed, CP = chromosome pair, m = metacentric, n = number of plates evaluated, p = short chromosome arm, q = long chromosome arm, SC = sex chromosome, SCS = sex chromosome system, sm = submetacentric, st = subtelocentric, t = terminal, ? = unknown, *X = data of other authors (X = reference number). [file 12862_2021_1750_MOESM7_ESM.doc]

| **Taxon** | **2n** | **SCS** | **Chromosome pairs:**  **number, morphology** | **Sex chromosome**  **morphology** | **NOR number**  **(CP/SC)** | **NOR-bearing CPs: number,**  **morphology (NOR location)** | **NOR-bearing sex chromosomes:**  **chromosome, morphology (NOR location)** | **Chiasma**  **frequency (n)** |
| --- | --- | --- | --- | --- | --- | --- | --- | --- |
| **Modisiminae** |  |  |  |  |  |  |  |  |
| *Anopsicus* sp. (cf. *iviei*) | 17 | X0 | 6m+2sm | Xm |  |  |  | allmost all biv. 1 chia. (5) |
| *Carapoia lutea**^26^ | 15 | X0 | 7m | Xm |  |  |  | 2.00 (?) |
| *Carapoia* sp.*^25^ | 15 | X0 | 7m | Xm |  |  |  | bivalents with 1 or 2 chia. (?) |
| *Mesabolivar brasiliensis**^27^ | 17 | X0 | 8bi | Xbi |  |  |  | 1.00 (?) |
| *M. cyaneotaeniatus**^27^ | 17 | X0 | 8bi | Xbi |  |  |  | 1.00 (?) |
| *M. spinulosus**^25^ | 17 | X0 | 3m+3sm+2st | Xm |  |  |  | bivalents with 1 or 2 chia. (?) |
| *M. togatus**^25^ | 17 | X0 | 5m+3sm | Xm |  |  |  | bivalents with 1 or 2 chia. (?) |
| *Modisimus* cf. *elongatus* | 17 | X0 | 5m+3sm | Xm |  |  |  | 1.19 (10) |
| *Psilochorus californiae* | 17 | X0 | 5m+2sm+1st | Xm | 1/2 | 1 sm (q, t) | X, m (1NOR p, t + 1NOR q, t) |  |
| *P. pallidulus* | 17 | X0 | 8m | Xm | 1/2 | 1 m (?) | X, m (1NOR p, t + 1NOR q, t) | 1.03 (10) |
| *P. simoni* | 17 | X0 | 6m+2sm | Xm |  |  |  | 1.03 (10) |
| **Ninetinae** |  |  |  |  |  |  |  |  |
| *Kambiwa neotropica* | 29 | X_1_X_2_X_3_X_4_Y | 12m | X_1_m+X_2_m+Xst+  X_4_st+Y? | 3/1 | 3 m (t) | X?, st (p, t) | 1.00 (5) |
| *Pholcophora americana* | 29 | X_1_X_2_Y | 12m+1sm | X_1_m+X_2_m+Ym | 2/0 | 2 bi (probably t) |  | 1.02 (10) |

**Table S3** Modisiminae and Ninetinae, summary of male cytogenetic data, including results of other authors. Doubtful data are not included. See database [24] for full list of published data on pholcid karyotypes, including doubtful data. Abbreviations: bi = biarmed, CP = chromosome pair, m = metacentric, n = number of plates evaluated, p = short chromosome arm, q = long chromosome arm, SC = sex chromosome, SCS = sex chromosome system, sm = submetacentric, st = subtelocentric, t = terminal, ? = unknown, *^X^ = data of other authors (^X^ = reference number).
