# Supplementary material for: Evolutionary pattern of karyotypes and meiosis in pholcid spiders (Araneae: Pholcidae): implications for reconstructing chromosome evolution of araneomorph spiders
Source: BMC Ecol Evol. 2021 May 3;21:75. doi: 10.1186/s12862-021-01750-8 (PMC8091558; doi:10.1186/s12862-021-01750-8)
Supplement: Supplementary file 12 — Additional file 12: Table S4. Pholcinae, summary of male cytogenetic data, including results of other authors. Doubtful data are not included. See database [24] for full list of published data on pholcid karyotypes, including doubtful data. Abbreviations: a = acrocentric, bi = biarmed, CP = chromosome pair, m = metacentric, n = number of plates evaluated, p = short chromosome arm, q = long chromosome arm, SC = sex chromosome, SCS = sex chromosome system, sm = submetacentric, ® = revision of data of other authors, st = subtelocentric, t = terminal, ? = unknown, *X = data of other authors (X = reference number). [file 12862_2021_1750_MOESM12_ESM.doc]

| **Taxon** | **2n** | **SCS** | **Chromosome pairs:**  **number, morphology** | **Sex chromosome**  **morphology** | **NOR number**  **(CP/SC)** | **NOR-bearing CPs: number,**  **morphology (NOR location)** | **NOR-bearing sex chromosomes:**  **chromosome, morphology (NOR location)** | **Chiasma**  **frequency (n)** |
| --- | --- | --- | --- | --- | --- | --- | --- | --- |
| **Pholcinae** |  |  |  |  |  |  |  |  |
| *Aetana kinabalu* | 23 | X_1_X_2_Y | 9m+1a | X_1_sm+X_2_m+Y? | 2/1 | 2 m (probably t) | X_1_, sm (t) | 1.00 (10) |
| *Belisana sabah* | 23 | X0 | 10m+1sm | Xm | 5/2 | 5 bi (t) | X, m (1NOR p, t + 1NOR q, t) | 1.00 (10) |
| *Cantikus sabah* | 13 | X0 | 6m | Xm | 1/0 | 1 m (q, t) |  | 1.05 (10) |
| *Leptopholcus guineensis* | 17 | X_1_X_2_Y | 4m+3sm | X_1_m+X_2_sm+Ysm |  |  |  | 1.03 (5) |
| *Metagonia* sp. (Br09-4) | 19 | X_1_X_2_Y |  |  |  |  |  | 1.00 (5) |
| *Micropholcus fauroti* ® | 9 | X0 | 4m | Xm | 1/0 | 1 m (t) |  | 1.08 (10) |
| *M. piaui**^25^ | 9 | X0 | 4m | Xm |  |  |  | 1.00 (?) |
| *M. ubajara**^25^ | 9 | X0 | 3m+1sm | Xm |  |  |  | bivalents with 1 or 2 chia. (?) |
| *Muruta tambunan* | 25 | X_1_X_2_Y |  | X_1_m+X_2_sm+Y? | 1/3 | 1 (t) | X_1_, m (1 NOR p, t + 1NOR q, t);  X_2_, sm (t) | 1.00 (10) |
| *Nipisa deelemanae* | 25 | X_1_X_2_Y |  | X_1_m+X_2_m+Y? | 4/5 | 2 probably bi (t);  1 bi (1NOR p, t + 1NOR q, t) | X_1_, m (1 NOR p, t + 1NOR q, t);  X_2_, m (1NOR p, t + 1NOR q, t);  Y, ? (t) | 1.07 (10) |
| *Pehrforsskalia conopyga* | 17 | X_1_X_2_Y | two CPs prominent |  |  |  |  | allmost all biv. 1 chia. (2) |
| *Pholcus bamboutos* | 23 | X_1_X_2_Y | most bi | X_1_m+X_2_m+Ym |  |  |  |  |
| *P. kindia* | 23 | X_1_X_2_Y | 8m+1sm+1st | X_1_m+X_2_m+Ym |  |  |  | 1.06 (10) |
| *P. opilionoides* | 25 | X_1_X_2_Y | 6m+5sm | X_1_m+X_2_a+Ym |  |  |  | 1.09 (10) |
| *P. pagbilao* | 23 | X_1_X_2_Y | 7m+3sm | X_1_m+X_2_a+Ysm | 5/0 | 3 bi (t);  1 bi (1NOR p, t + 1NOR q, t) |  | 1.01 (10) |
| *P. phalangioides* | 25 | X_1_X_2_Y | 9m+2sm | X_1_m+X_2_sm+Ym | 3/3 | 3 bi (t) | X_1_, m (1 NOR p, t + 1NOR q, t);  X_2_, sm (q, t) | 1.01 (40) |
| *Pholcus* sp. | 25 | X_1_X_2_Y | 7m+3sm+1a | X_1_m+X_2_st+Ym |  |  |  | 1.13 (10) |
| *Quamtana filmeri* | 17 | X_1_X_2_Y | 4m+1sm+2st | X_1_m+X_2_st+Ym | 1/2 | 1, ? (t) | X_1_, m (1 NOR p, t + 1NOR q, t) | 1.11 (10) |
| *Q. hectori* | 23 | X_1_X_2_Y | 10m | X_1_m+X_2_a+Ym | 2/2 | 2 m (t) | X_1_, m (1 NOR p, t + 1NOR q, t) | 1.00 (3) |
| *Spermophora senoculata* ® | 25 | X_1_X_2_Y | 11m | X_1_m+X_2_m+Ym | 3/0 | 3 m (?) |  | 1.01 (20) |

**Table S4** Pholcinae, summary of male cytogenetic data, including results of other authors. Doubtful data are not included. See database [24] for full list of published data on pholcid karyotypes, including doubtful data. Abbreviations: a = acrocentric, bi = biarmed, CP = chromosome pair, m = metacentric, n = number of plates evaluated, p = short chromosome arm, q = long chromosome arm, SC = sex chromosome, SCS = sex chromosome system, sm = submetacentric, ® = revision of data of other authors, st = subtelocentric, t = terminal, ? = unknown, *^X^ = data of other authors (^X^ = reference number).
