# Supplementary material for: Evolutionary pattern of karyotypes and meiosis in pholcid spiders (Araneae: Pholcidae): implications for reconstructing chromosome evolution of araneomorph spiders
Source: BMC Ecol Evol. 2021 May 3;21:75. doi: 10.1186/s12862-021-01750-8 (PMC8091558; doi:10.1186/s12862-021-01750-8)
Supplement: Supplementary file 19 — Additional file 19: Table S5. Smeringopinae, summary of male cytogenetic data, including results of other authors. Doubtful data are not included. See database [24] for full list of published data on pholcid karyotypes including doubtful data. Abbreviations: a = acrocentric, bi = biarmed, CP = chromosome pair, m = metacentric, n = number of plates evaluated, p = short chromosome arm, q = long chromosome arm, SC = sex chromosome, SCS = sex chromosome system, sm = submetacentric, ® = revision of data of other authors, st = subtelocentric, t = terminal, ? = unknown, *X = data of other authors (X = reference number). [file 12862_2021_1750_MOESM19_ESM.doc]

| **Taxon** | **2n** | **SCS** | **Chromosome pairs:**  **number, morphology** | **Sex chromosome**  **morphology** | **NOR number**  **(CP/SC)** | **NOR-bearing CPs: number,**  **morphology (NOR location)** | **NOR-bearing sex chromosomes:**  **chromosome, morphology (NOR location)** | **Chiasma**  **frequency (n)** |
| --- | --- | --- | --- | --- | --- | --- | --- | --- |
| **Smeringopinae** |  |  |  |  |  |  |  |  |
| *Crossopriza lyoni* ® | 23 | X0 | 9m+2sm | Xm |  | 1 bi (p,t); 1 bi (q,t)*^28^ | X, m (t)*^28^ | 1.00 (10) |
| *Crossopriza* sp. | 23 | X0 | 7m+4sm | Xm |  |  |  | 1.00 (10) |
| *Holocnemus caudatus* | 23 | X0 | 8m+3sm | Xm |  |  |  | 1.00 (50) |
| *H. hispanicus* | 23 | X0 | 7m+3sm+1st | Xm | 1/1 | 1 m (t) | X, m (t) | 1.00 (10) |
| *H. pluchei* ® | 27 | X0 | 9m+3sm+1st | Xm | 2/0 | 2 probably bi (t?) |  | 1.00 (5) |
| *Hoplopholcus cecconii* | 28 | X_1_X_2_0 | 12m+1sm | X_1_m+X_2_st |  |  |  | 1.00 (4) |
| *H. forskali* | 28 | X_1_X_2_0 | 12m+1st | X_1_m+X_2_st | 1/0 | 1 m (t) |  | 1.00 (10) |
| *H. labyrinthi* | 28 | X_1_X_2_0 | 10m+2sm+1st | X_1_m+X_2_st | 1/0 | 1 probably bi (t) |  | 1.01 (10) |
| *Smeringopus atomarius* | 28 | X_1_X_2_0 | 11m+1sm+1a | X_1_m+X_2_sm | 2/0 | 2 probably bi (?) |  | 1.00 (5) |
| *S. cylindrogaster* | 26 | X_1_X_2_0 | 10m+1sm+1a | X_1_m+X_2_a |  |  |  | 1.02 (5) |
| *S. ndumo* | 28 | X_1_X_2_0 | 11m+2sm | X_1_m+X_2_m |  |  |  | 1.01 (10) |
| *S. pallidus* | 28 | X_1_X_2_X_3_0 | 10m+3sm | X_1_m+X_2_m+X_3_m | 2/0 | 1 m (q, t); 1 sm (p, t) |  | 1.00 (10) |
| *S. peregrinus* | 28 | X_1_X_2_0 | 9m+3sm+1st | X_1_m+X_2_st |  |  |  | 1.00 (3) |
| *S. similis* | 28 | X_1_X_2_0 | 10m+2sm+1st | X_1_m+X_2_m |  |  |  | 1.02 (10) |
| *Smeringopus* sp. | 28 | X_1_X_2_0 | 10m+2sm +1a | X_1_m+X_2_a | 4/0 | 4 bi (t) |  | 1.00 (2) |
| *Stygopholcus skotophilus* | 28 | X0 | 7m+3sm+1st | Xm | 1/0 | 1 bi (t) |  | 1.05 (10) |

**Table S5** Smeringopinae, summary of male cytogenetic data, including results of other authors. Doubtful data are not included. See database [24] for full list of published data on pholcid karyotypes, including doubtful data. Abbreviations: a = acrocentric, bi = biarmed, CP = chromosome pair, m = metacentric, n = number of plates evaluated, p = short chromosome arm, q = long chromosome arm, SC = sex chromosome, SCS = sex chromosome system, sm = submetacentric, ® = revision of data of other authors, st = subtelocentric, t = terminal, ? = unknown, *^X^ = data of other authors (^X^ = reference number).
