# Supplementary material for: Evolutionary pattern of karyotypes and meiosis in pholcid spiders (Araneae: Pholcidae): implications for reconstructing chromosome evolution of araneomorph spiders
Source: BMC Ecol Evol. 2021 May 3;21:75. doi: 10.1186/s12862-021-01750-8 (PMC8091558; doi:10.1186/s12862-021-01750-8)
Supplement: Supplementary file 28 — Additional file 28: Appendix S1. Evolution of particular chromosomes of the pholcid X1X2Y system. [file 12862_2021_1750_MOESM28_ESM.doc]

**Evolution of particular chromosomes of the pholcid X_1_X_2_Y system**

**Evolution of the X_1_ chromosome**

The morphology of the pholcid X_1_ chromosome is conservative. This chromosome is usually the largest chromosome of the karyotype. It is always metacentric, except for *Aetana*, which exhibits a submetacentric X_1_ chromosome. Even though the *Aetana* X_1_ chromosome has undergone such a change in morphology, its size is similar to that found in another early-diverging member of the subfamily Pholcinae, *Spermophora* (Additional file 1: Table S1). This pattern suggests an origin of the submetacentric X_1_ chromosome by pericentric inversion.

Unlike morphology, the X_1_ size varies greatly, namely from 8.6% (*Artema atlanta*) to 16.3 (*Leptopholcus guineensis*) and 17.1% of TCL (*Pholcus kindia*). We assume that the X_1_ chromosome of the latter two species could expand by integration of material from CPs, which is indicated by the reduction in the number of chromosomal pairs in these species when compared to related taxa. In *Leptopholcus*, the reduction of NCPs is accompanied by an expansion of three chromosomal pairs, plus the X_1_, and Y chromosomes, which indicates integration of material from CPs into these elements. Concerning *P. kindia*, only the size of the X_1_ and Y chromosomes is increased, which indicates insertion of fragments from CPs into these chromosomes only. Another source of enlargement of X_1_ in this species could be the X_2_ chromosome, which is reduced.

**Evolution of the X_2_ chromosome**

The most dynamic element of the pholcid X_1_X_2_Y system is the X_2_ chromosome. Its size ranged considerably from 2.7 % (*Pholcus pagbilao*) to 9.4% of TCL (*Pholcophora americana*). In contrast to the X_1_ chromosome, this element has often undergone reduction during evolution. Mapping of our data on the molecular tree indicates two reductions of the X_2_ chromosome, namely in 1) the ancestor of *Artema* (X_2_ size ˂ 6.6% of TCL), and 2) the ancestor of a pholcine clade including the genera *Leptopholcus*, *Pholcus*, and *Quamtana* (X_2_ size ˂ 7% of TCL). Reduction of the X_2_ chromosome continued during the evolution of pholcines (Additional file 31: Fig. S25). We suppose that the reduction of the X_2_ chromosome has been accompanied by the integration of its fragments into the X_1_ chromosome. In agreement with this hypothesis, average relative size of the X_1_ chromosome is higher in pholcines with a reduced X_2_ than in pholcines without reduction of this element (*Aetana*, *Spermophora*). Integrations of X chromosome fragments into the Y chromosome are improbable. The chromosomes X and Y of pholcids do not recombine, which suggests their considerable structural difference. The specific genetic content of X chromosome fragments integrated into the Y chromosome would most probably result in a collapse of the sexual phenotype. Although reduced X_2_ chromosomes retained their metacentric morphology in several pholcids (*Nipisa*, some *Pholcus* species), they are more often changed to submetacentric or even monoarmed elements, namely in *Artema atlanta* (Arteminae), *Leptopholcus*, *Muruta*, *Quamtana*, and most *Pholcus* species (Pholcinae).

An increase of the X_2_ chromosome was much less common than the reduction of this element during pholcid evolution. It is only indicated in *Quamtana filmeri*. Notably, this species also exhibits a considerable reduction of NCPs. Therefore, the increase of its X_2_ chromosome could be caused by material derived from CPs. Integration of autosome segments into the sex chromosomes is relatively common during karyotype evolution. It has been found, for example, in beetles, mammals, and dicotyledons [84, 85, 86].

**Evolution of Y chromosome**

The morphology of the pholcid Y chromosome is conservative. Although it was impossible to determine its morphology in several species, it was metacentric in other pholcids except for *Leptopholcus guineensis* and *Pholcus pagbilao*, which have a submetacentric Y chromosome. Unlike morphology, the size of the Y chromosome ranges considerably, from 1.2 % (*Artema nephilit*) to 11.7 % of TCL (*Pholcus kindia*). Representatives of *Artema* (Arteminae), *Pholcophora* (Ninetinae), *Aetana*, and *Spermophora* (Pholcinae) possess a Y microchromosome (size ˂ 3% of TCL). Although the relative size of the Y chromosome of *Nipisa* was not determined, this pholcid probably possesses a Y microchromosome as well. The size of the Y chromosome has increased considerably in an ancestor of the clade including the genera *Leptopholcus*, *Pholcus*, *Muruta*, *Nipisa*, and *Quamtana* (Pholcinae). The size of the Y chromosome in this lineage ranges from 4.8 % (*Pholcus opilionoides*) to 11.7 % of TCL (*P. kindia*). The Y chromosome of *Nipisa* was probably reduced secondarily. Concerning other haplogynes with the X_1_X_2_Y system, an increase of the Y chromosome has only been reported in a pacullid [14]. Although an increase of the Y chromosome during evolution is an uncommon process, is has also been reported in some other organisms. In general, there are two fundamental mechanisms of enlargement of the Y chromosome, i.e. expansion of repetitive sequences and integration of autosome material (e.g. [87, 88]). Concerning pholcids, the first mechanism is suggested by a predomination of constitutive heterochromatin on the Y chromosome of *P. phalangioides* [9]. The second mechanism is indicated by an increase in the Y chromosome in taxa whose NCPs are reduced in comparison with close relatives (*Leptopholcus*, *Pholcus* species with 2n♂ = 23, *Quamtana filmeri*). Following the integration into the Y chromosome, the fragments could undergo fast degeneration, due to the absence of recombination and the invasion of repetitive sequences from constitutive heterochromatin. On the other hand, translocation of regions from CPs on the Y chromosome could decelerate its degeneration and increase its evolutionary stability.
