# Supplementary material for: Evolutionary pattern of karyotypes and meiosis in pholcid spiders (Araneae: Pholcidae): implications for reconstructing chromosome evolution of araneomorph spiders
Source: BMC Ecol Evol. 2021 May 3;21:75. doi: 10.1186/s12862-021-01750-8 (PMC8091558; doi:10.1186/s12862-021-01750-8)
Supplement: Supplementary file 32 — Additional file 32: Table S6. Species studied, their instar, sex, collecting data and depositories. Abbreviations: AM = Australian Museum, Sydney, Australia (specimen KS 128687), Co. = county, Hwy = highway, Isl. = island, m = male, Mts. = mountains, N = north, N.P. = national park, NW = northwest, S = south, SL = specimens lost or discarded, sm = subadult male, W = west, ZFMK = Zoological Research Museum Alexander Koenig. [file 12862_2021_1750_MOESM32_ESM.doc]

| **Taxon** | **Individuals** | **Locality** | **Latitude** | **Longitude** | **Deposition** |
| --- | --- | --- | --- | --- | --- |
| **Arteminae** |  |  |  |  |  |
| *Artema atlanta* | 1m | South Africa, Mpumalanga, Barberton 1 | -25.6030 | 30.9749 | ZFMK |
| *Artema nephilit* | 2m | Israel, Southern District, shore of southern Dead Sea, Arubotayim cave | 31.1016 | 35.3900 | ZFMK |
| *Chisosa diluta* | 2m | USA, Texas, Big Bend N.P., Tuff Canyon | 29.500 | -103.880 | ZFMK |
| *Holocneminus* sp. (Phi9) | 1sm | Philippines, Negros Oriental Prov., Negros Isl., Twin Lakes N.P. | 9.3665 | 123.1815 | ZFMK |
| *Physocyclus dugesi* | 1m | Mexico, Morelos State, Anenecuilco near Cuautla | 18.7830 | -98.9903 | ZFMK |
| *Wugigarra* sp. | 3m | Australia, Queensland, D´Aguilar N.P., Mount Glorious, Lepidozamia road | -27.2971 | 152.7530 | AM |
| **Modisiminae** |  |  |  |  |  |
| *Anopsicus* sp. (cf. *iviei*) | 1m | Mexico, Veracruz State, Los Tuxtlas Mts., Tropical Biology Station of UNAM | 18.5833 | -95.1083 | ZFMK |
| *Modisimus* cf. *elongatus* | 1m | Cuba, Pinar del Rio, Viñales, "site 1" | 22.6220 | -83.7370 | ZFMK |
| *Psilochorus californiae* | 2m | USA, California, Riverside Co., Hwy 243, Banning, 2.9 km S of Black Mountain | 33.7910 | -116.7455 | ZFMK |
| *Psilochorus pallidulus* | 2m | USA, Texas, Big Bend N.P., Tuff Canyon | 29.500 | -103.880 | ZFMK |
| *Psilochorus simoni* | 3m | Germany, Bonn, house | 50.6870 | 7.1450 | SL |
| **Ninetinae** |  |  |  |  |  |
| *Kambiwa neotropica* | 2m | Brazil, Rio Grande do Norte, near Felipe Guerra, Lajedo do Arapuá | -5.5292 | -37.6143 | ZFMK |
| *Pholcophora americana* | 1m | Canada, British Columbia, lower Ashnola River valley, ~12 km west of Keremeos | 49.2165 | -119.9678 | ZFMK |
|  | 1m | USA, Colorado, Lookout Mountain near Golden | 39.7329 | -105.2391 | ZFMK |
|  | 2m | USA, NW Montana, Sanders Co., near Trout Creek, Hwy 200 | 47.8166 | -115.5659 | ZFMK |
|  | 4m | USA, Washington, Whitman Co., top of Steptoe Butte, near State Hwy 195 | 47.0326 | -117.2968 | ZFMK |
| **Pholcinae** |  |  |  |  |  |
| *Aetana kinabalu* | 2m | Malaysia, Borneo, Sarawak, Bario, forest along river W of town | 3.7360 | 115.4400 | ZFMK |
| *Belisana sabah* | 3m | Malaysia, Borneo, Sabah, Sepilok, Rainforest Discovery Centre, forest along Pitta Trail | 5.8765 | 117.9395 | ZFMK |
| *Cantikus sabah* | 2m | Malaysia, Borneo, Sabah, Sepilok, Rainforest Discovery Centre, forest along Pitta Trail | 5.8765 | 117.9395 | SL |
| *Leptopholcus guineensis* | 1m | Guinea, Basse-Guinée, near Kindia | 10.0133 | -12.8100 | ZFMK |
| *Metagonia* sp. (Br09-4) | 2m | Brazil, Rio de Janeiro, near Santa Maria Madalena | -21.9833 | -41.9567 | ZFMK |

| **Taxon** | **Individuals** | | **Locality** | | **Latitude** | | **Longitude** | | **Deposition** | |  |
| --- | --- | --- | --- | --- | --- | --- | --- | --- | --- | --- | --- |
| *Micropholcus fauroti* | 2m | | Brazil, Rio Grande do Norte, Apodi | | -5.6600 | | -37.8000 | | ZFMK | |  |
|  | 1m | | Cape Verde, Boa Vista Isl., Iberostar hotel | | 16.177 | | -22.918 | | ZFMK | |  |
|  | 1m | | South Africa, KwaZulu-Natal, Ndumo Game Reserve, Nyamiti road | | -26.9022 | | 32.2666 | | ZFMK | |  |
| *Muruta tambunan* | 2m | | Malaysia, Borneo, Sabah, Mt. Kinabalu, forest along Silau Silau Trail | | 6.0135 | | 116.5400 | | ZFMK | |  |
| *Nipisa deelemanae* | 3m | | Malaysia, Borneo, Sabah, Sepilok, Rainforest Discovery Centre, forest along Pitta Trail | | 5.8765 | | 117.9395 | | ZFMK | |  |
| *Pehrforsskalia conopyga* | 1m | | Israel, Northern District, kibbutz Degania Alef | | 32.7040 | | 35.5800 | | ZFMK | |  |
| *Pholcus bamboutos* | 1m | | Cameroon, West Region, Bamboutos district, near Mbouda | | 5.6812 | | 10.2151 | | ZFMK | |  |
| *Pholcus kindia* | 1m | | Guinea, Basse-Guinée, near Kindia | | 10.0133 | | -12.8100 | | ZFMK | |  |
| *Pholcus opilionoides* | 2m | | Czech Republic, Heřmánky near Jakubčovice n. Odrou, Hanlův lom quarry | | 49.7042 | | 17.7646 | | SL | |  |
|  | 2m | | Czech Republic, Žleby | | 49.8910 | | 15.4720 | | SL | |  |
| *Pholcus pagbilao* | 1m | | Philippines, Cebu Isl., Moalboal, Busay Cave | | 9.9160 | | 123.4370 | | ZFMK | |  |
| *Pholcus phalangioides* | 12m, 11 sm | | Czech Republic, České Budějovice | | 48.9772 | | 14.4471 | | SL | |  |
|  | 5sm | | Czech Republic, Ústí nad Labem | | 50.6652 | | 14.0037 | | SL | |  |
|  | 19m | | Czech Republic, Prague | | 50.0716 | | 14.4241 | | SL | |  |
|  | 4m | | Czech Republic, Pardubice | | 50.0414 | | 15.7769 | | SL | |  |
|  | 5m | | Czech Republic, Žilina near Nový Jičín | | 49.5855 | | 18.0333 | | SL | |  |
| *Pholcus* sp. | 1m | | Kazakhstan, 10km N of Kapchagay, Itzhon Plateau, left river side of Ili river | | 43.9577 | | 77.0439 | | SL | |  |
| *Quamtana filmeri* | 2m | | South Africa, KwaZulu-Natal, Drakensberg A | | -28.6909 | | 28.9414 | | ZFMK | |  |
|  | 2m | | South Africa, KwaZulu-Natal, Drakensberg A1 | | -28.6909 | | 28.9414 | | ZFMK | |  |
|  | 1m | | South Africa, KwaZulu-Natal, Drakensberg B | | -28.6861 | | 28.9278 | | ZFMK | |  |
| *Quamtana hectori* | 6m | | South Africa, KwaZulu-Natal, Pongola Game Reserve | | -27.3601 | | 31.9848 | | ZFMK | |  |
|  | 2m | | South Africa, KwaZulu-Natal, Nyamiti road | | -26.9022 | | 32.2666 | | ZFMK | |  |
|  | 1m | | South Africa, Mpumalanga, Barberton 3 | | -25.6770 | | 31.1392 | | ZFMK | |  |
| *Spermophora senoculata* | 14m | | Portugal, Mitra near Évora | | 38.5291 | | -8.0168 | | SL | |  |
|  | 10m | | Republic of North Macedonia, Sokolarci, xerophyte pasture | | 41.9073 | | 22.2763 | | ZFMK | |  |
| **Smeringopinae** |  | |  | |  | |  | |  | |  |
| *Crossopriza lyoni* | 2m | | Vietnam, breeding | |  | |  | | ZFMK | |  |
| *Crossopriza* sp. | 1m | | Morocco, Larache, archaeological site of Lixus | | 35.1996 | | -6.1089 | | ZFMK | |  |
| *Holocnemus caudatus* | 3m, 2 sm | | Portugal, Mitra near Évora | | 38.5291 | | -8.0168 | | ZFMK | |  |
| *Holocnemus hispanicus* | 3m | | Spain, unspecified port at southern coast | | unknown | | unknown | | ZFMK | |  |
| **Taxon** | | **Individuals** | | **Locality** | | **Latitude** | | **Longitude** | | **Deposition** | |
| *Holocnemus pluchei* | | 2m | | USA, Colorado, Yarnell (breeding of Spiderpharm company) | |  | |  | | ZFMK | |
|  | | 4m | | Croatia, Čijovo Isl., Slatine village | | 43.4995 | | 16.3350 | | ZFMK | |
|  | | 3m | | France, Corse, Capitello, Prunelli-di-Fiumorbo, confluence of Granona and Prunelli rivers | | 42.0103 | | 9.3254 | | ZFMK | |
|  | | 3m | | Italy, Catania | | 37.4931 | | 15.0693 | | ZFMK | |
|  | | 1m | | Cyprus, Agios Georgios Pegeia near Akamas | | 34.9012 | | 32.3248 | | ZFMK | |
|  | | 1m | | Jordan, Wadi Rum, synanthropic (desert camp) | | 29.4814 | | 35.4093 | | ZFMK | |
| *Hoplopholcus cecconii* | | 2m | | Israel, Mt. Meron, Nature Reserve, close to Field School | | 33.0132 | | 35.3891 | | ZFMK | |
| *Hoplopholcus forskali* | | 2m | | Hungary, Balatonalmadi | | 47.0252 | | 18.0042 | | ZFMK | |
|  | | 1m | | Hungary, Vác | | 47.7977 | | 19.1402 | | ZFMK | |
| *Hoplopholcus labyrinthi* | | 2m | | Greece, Crete, Melidoni, Melidoni Cave | | 35.3848 | | 24.7302 | | ZFMK | |
| *Smeringopus atomarius* | | 1m | | Namibia, 45 km NW of Betta | | -25.1511 | | 16.1062 | | ZFMK | |
|  | | 1m | | Namibia, northern part of Namib-Naukluft | | -23.0208 | | 14.8524 | | ZFMK | |
| *Smeringopus cylindrogaster* | | 1m | | Cameroon, Littoral Region, Loum | | 4.7267 | | 9.7083 | | ZFMK | |
| *Smeringopus ndumo* | | 2m | | South Africa, KwaZulu-Natal, Ndumo Game Reserve, Crocodile Farm | | -26.9094 | | 32.3139 | | ZFMK | |
| *Smeringopus pallidus* | | 2m  2m | | Philippines, Mindanao Isl., Salumay, Mt. Malambo (locality Ph 6)  Cape Verde, Boa Vista Isl., Iberostar hotel | | 7.4971  16.177 | | 125.2697  -22.918 | | ZFMK  ZFMK | |
| *Smeringopus peregrinus* | | 1m | | Madagascar, Toamasina province, Alaotra lake, Andreba village, camp | | -17.6302 | | 48.5049 | | SL | |
| *Smeringopus similis* | | 1m | | Namibia, Otavi town, camp | | -19.6334 | | 17.3361 | | ZFMK | |
| *Smeringopus* sp. | | 2m | | South Africa, Northern Cape, close to Vioolsdrift Border Control | | -28.7036 | | 17.5951 | | SL | |
| *Stygopholcus skotophilus* | | 1m | | Federation of Bosnia and Herzegovina, near Trebinje, St. Pauľs cave | | 42.7081 | | 18.3502 | | ZFMK | |

**Additional file 32 (doc): Table S6** Species studied, their instar, sex, collecting data and depositories. Abbreviations: AM = Australian Museum, Sydney, Australia (specimen KS 128687), Co. = county, Hwy = highway, Isl. = island, m = male, Mts. = mountains, N = north, N.P. = national park, NW = northwest, S = south, SL = specimens lost or discarded, sm = subadult male, W = west, ZFMK = Zoological Research Museum Alexander Koenig.
